# A Series of Trigonal Arylimido Iron Complexes and the Influence of Oxidation State and Steric Demand on Reactivity

**DOI:** 10.1021/acs.inorgchem.5c03148

**Published:** 2025-11-07

**Authors:** Andres Gonzalez, Sascha Reith, Alexander Reckziegel, C. Gunnar Werncke

**Affiliations:** † 9377 Philipps-University Marburg, Hans-Meerwein-Straße 4, Marburg D-35032, Germany; ‡ 9180 Leipzig University, Johannisallee 29, Leipzig D-04103, Germany

## Abstract

We describe the reaction between the linear iron­(I/II) silylamides [FeL_2_]^0,–^ (L = N­{Dipp}­SiMe_3_) and aryl azides N_3_R with different steric demand (R = Ph, 2-Me-C_6_H_4_ (Tol), 2,4-Me_2_-C_6_H_3_ (Xyl), 2,6-^*i*^Pr_2_-C_6_H_3_ (Dipp), 2,4,6-^*i*^Pr_3_-C_6_H_2_ (Tripp)). Obtained trigonal anionic high-spin iron­(II) imidyl complexes exhibit long Fe–N bonds of approximately 1.76 Å. In case of the largest substituent (R = Tripp) a slow SiMe_3_-shift from the ancillary ligand to the imido nitrogen is observed. In contrast, for the smallest azide (PhN_3_) the tetrazenido complex [Fe­(η^2^-κN:^1^ κN^4^-N_4_Ph_2_)­L_2_] is obtained. Isolable imido complexes react nucleophilic toward benzaldehyde and mesityl isocyanate under cycloaddition to metallacycles. In case of the neutral precursor [FeL_2_] imido iron species were only obtained for R = Tol or Xyl, with Fe–N bonds of approximately 1.74 Å. Based on computational analysis these compounds exhibit isoenergetic quintet and triplet states with shared electronic structures of an iron­(IV) imide, iron­(III) imidyl and iron­(II) nitrene. For the smallest aromatic azide PhN_3_, the reaction with FeL_2_ results directly in C–H amination of the ancillary ligands. For sterically more encumbered neutral imido complexes, intramolecular C–H amination requires heating or irradiation. The observed absence for intramolecular C–H amination of anionic imido complexes is computationally rationalized.

## Graphical abstract

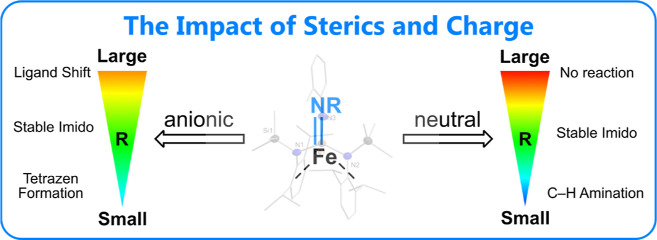

## Introduction

3d-Metal imido complexes are key intermediates in the catalytic amination of unfunctionalized C–H bonds via formal nitrene insertion.^1–7^ These imido metal complexes are classically described as a metal bound dianionic imide NR^2–^, as it is the case for early transition metal complexes.^8^ When going to later 3d-metals the number of d-electrons increases. It leads to population of antibonding π-orbitals and thus weakening of the imido metal bond. Furthermore, the imido–metal interaction becomes more covalent so that an imidyl [NR]^•–^ or a metal nitrene [NR]^0^ resonance structure is increasingly relevant.^9–11^ These particular electronic structures are used to rationalize H atom abstraction (HAA) and/or nitrene transfer capabilities. In this context, authenticated 3d-metal bound imidyls^12–19^ and especially nitrenes^20,21^ are still scarce due to their intrinsic high reactivity. As such the factors that contribute to their C–H activation reactivity (e.g., oxidation state, complex geometry, charge or spin state) are not fully understood and furthermore difficult to disentangle from each other. Looking at iron, most isolated imido compounds are low-spin iron imide complexes. These exhibit limited reactivity, which facilitated their isolation.^9,22–28^ The seminal example of an imidyl iron type complex constitutes a high-spin iron dipyrromethene based complex from Betley in 2011.^18^ A few other examples were reported since then, including contributions from our group.^11,18,29–32^ The situation for iron nitrenes is ambiguous: Some neutral trigonal iron imidos have been reported by Lin, Albrecht and our group, which exhibit a rather covalent bond. Computational analysis on the CASSCF level showed the relevance of an iron nitrene character with contributions from iron imidyl and iron imide resonance structures.^15,33–35^ Of these imidyl and nitrene complexes the trigonal [Fe­(NMes)­(N­{Dipp}­SiMe_3_)_2_]^−,0^ reported by us was informative as it allowed to obtain first insights into the impact of charge and oxidation state onto the reactivity. The neutral compound reacted slightly electrophilic, whereas the reduced, anionic form behaved nucleophilic toward CS_2_.^15^

Before this background, as well as the very limited number of iron imido complexes in higher spin states in general, we now present a series of anionic and neutral high-spin imido iron complexes bearing aryl substituents with different steric profiles–especially in the ortho- and para positions. Through this, the steric boundaries within this low-coordinate ligand framework are obtained and intrinsic reactivity patterns, namely oxidation state dependent tetrazenido formation, SiMe_3_-shift, intramolecular C–H amination, as well as reaction with carbonyl group containing substrates are revealed.

## Results and Discussion

Anionic imido complexes were obtained from the reaction of a precooled solution (−30 °C) of the linear iron­(I) complex K­{m}­[FeL_2_] (m = crypt.222 or 18c6, L = N­(Dipp)­SiMe_3_, Dipp = 2,6-^*i*^Pr_2_-C_6_H_3_)^15,36^ with a variety of aromatic organo azides RN_3_ at – 30 C in a mixture of Et_2_O/THF (3:1) (Scheme 1; Figure 1, left and center left). Isolation of the crystalline imido complexes of the type K­{m}­[Fe­(NR)­L_2_] (R = 2-Me-C_6_H_4_ = Tol (**1**), 2,6-Me_2_-C_6_H_3_ = Xyl (**2**), 2,6-^*i*^Pr_2_-C_6_H_3_ = Dipp (**3**)) was accomplished by layering the reaction solutions with *n*-pentane. For **4** (R = 2,4,6-^*i*^Pr_3_-C_6_H_2_ = Tripp) crystals suitable for X-ray diffraction analysis could only be grown from a *n*-pentane layered saturated 1,2-difluorobenzene solution. Prolonged standing of **4** provided further crystals of K­{crypt.222}­[Fe­(NDipp)-(N­{Tripp}­SiMe_3_)­L], **5**, in which one of the SiMe_3_ units shifted from the ancillary silylamide to the NTripp imido function. This behavior points to a high nucleophilicity of the imido nitrogen and aligns with observations made for anionic nickel or cobalt imido complexes.^16,37^ With PhN_3_, the tetrazenido complex K­{18c6}­[Fe­(η^2^-κN:^1^ κN^4^-N_4_Ph_2_)­L_2_], **6**, was obtained (Figure 1, center right). **6** likely resulted from initial formation of K­{m}­[Fe­(NPh)­L_2_] and subsequent reaction with a second equivalent of PhN_3_. The tetrazenide formation was faster than the initial imido formation, as the presence of the latter could not be evidenced via stoichiometric variations or in situ ^1^H NMR spectroscopic studies.

**Scheme 1:**
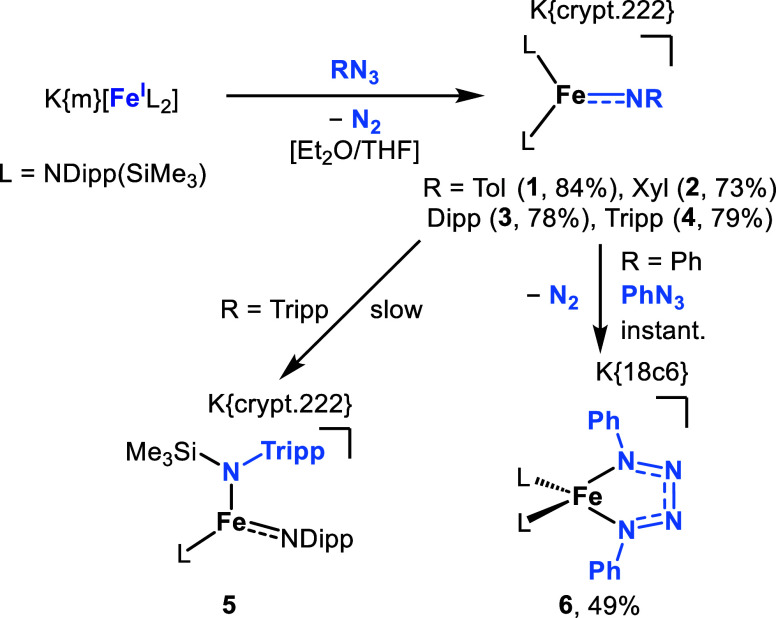
Synthesis of Anionic Imido Iron Complexes and Subsequent Bond Transformations

**Figure 1:**
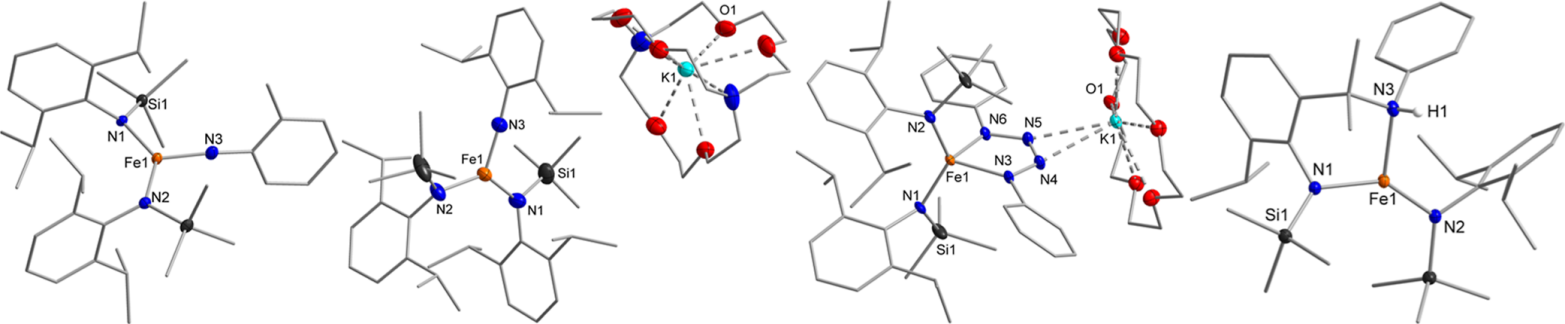
Exemplary molecular structures of imido complexes (left, shown for **1** without the K­{crypt.222} counterion), complex **3** with counterion (center left), the tetrazenido complex **6** (center right) and nitrene insertion products (right, shown for **9**). Relevant bond lengths and angles are found in Table 1 (Hydrogen atoms are generally hidden for clarity).

The neutral iron imido complexes were obtained by the reaction of the azides RN_3_ with a precooled solution (−30 °C) of [FeL_2_] in *n*-pentane (Scheme 2) for R = Tol (**7**) and Xyl (**8**), adding to previously reported [Fe­(NMes)­L_2_].^15^ For the larger organo azides N_3_Dipp or N_3_Tripp no reaction was observed. Using PhN_3_, [Fe­(κ^2^-N-N­(SiMe_3_)-2-(CH_3_)_2_CNH­{Ph}-6-^*i*^Pr-phenyl)­L], **9**, was isolated in which one of the ^*i*^Pr groups was aminated (Figure 1, right). It presumably results from initial formation of the imido species [Fe­(NPh)­L_2_] that subsequently undergoes nitrene insertion into a C–H bond of the ^*i*^Pr group of the ancillary Dipp-functions. Intramolecular amination of an ancillary ligand was already observed for the reaction of [Fe­(N­{SiMe_3_}_2_)_2_] or [Fe­(N­{Dipp}­CMe_3_)_2_] with aliphatic^33–35,38^ but not with aromatic azides. The rapid formation of **9** led us to consider if the slightly more encumbered **7** (Tol) and **8** (Xyl) could likewise undergo C–H amination.

**Scheme 2:**
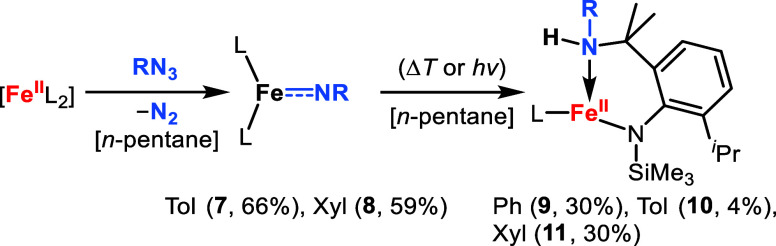
Synthesis of Neutral Imido Iron Complexes and Subsequent Intramolecular C–H Amination^a^

a Deviating reaction conditions for C–H amination of **10** and **11**: *n*-heptane, 80 °C, 2 h or *n*-pentane, 254 nm, 16 h.

Both compounds were heated for 2 h at 80 °C in *n*-heptane. Crystallization from a concentrated solution of hexamethyldisiloxane (HMDSO) resulted in the isolation of the respective intramolecular insertion products **10** (Tol, 4%) and **11** (Xyl, 30%). The low to moderate yields is attributed to the extremely high solubility of these compounds. We also examined the photochemical transformation of **7** and **8** in solution, but irradiation with 395 nm in *n*-pentane yielded no noticeable transformation. Only by using a Hg-lamp (254 nm) some crystalline **10** and **11** were obtained, however in very low yields (<5%). All isolated imido complexes feature in the solid state a trigonal iron center with very similar Fe–N_imido_ (1.746–1.777 Å), N3–C_Ar_ (1.332–1.355 Å) and Fe1–N3–C_Ar_ (170.9–176.1°) bond lengths and angles (Table 1). Compared to the anionic complexes, the Fe–N_imido_ bond lengths in the neutral complexes are slightly contracted (Δ*d* ≈ – 0.03 Å), with the effect being more pronounced for the ancillary Fe–N_amido_ bonds (Δ*d* ≈ – 0.05 Å). It reveals an overall marginal impact of the charge and formal oxidation state on these central structural metrics. The small variations on the Fe–N–Aryl bond angles in the imido complexes reveal further a minimal effect of the substitution pattern in 2- and/or 6-positions and thus it implies a preference for a rather linear [FeNR] axis for all aromatic imido complexes. This behavior can be attributed to electronic stabilization via delocalization of spin and electron density from the imido iron unit over the aromatic ring for both anionic and neutral aromatic complexes, as proposed for other imidyl type complexes.^13,31,38,39^ It is interesting to note that for the 2-methyl substituted complexes **1** and **7**, a structural disorder is observed in solid state. It slightly affects the imido iron unit with regards to the Fe–N bond length and bond angle, and is absent for 2,6-substituted compounds. It implicated that the imido metal bond can be easily distorted. The molecular structure of the insertion products **9**, **10** and **11** exhibit more obtuse N1–Fe1–N2 (138.9–149.1°) angles than those found in the imido complexes (Table 1), which is unsurprising given the chelating nature of the aminated amide ligand. The Fe1–N3 bond lengths amount to 2.120–2.149 Å, which is in line with neutral amine or organo azide adducts of iron­(II).^33,35,38^ Interestingly, the ancillary Fe–N_amido_ bonds (1.901–1.928 Å) are only marginally longer than in the imido complexes, which implies a comparable oxidation state and charge of iron in the neutral imido complexes and in the insertion products. All imido (and subsequent) complexes show distinct paramagnetic features in solution. The magnetic measurements using the Evans method were performed on all neutral and anionic imido species (Table 1). The anionic compounds exhibit values between 4.94 (**4**) and 5.68 μ_Β_ (**3**). These are lower than the expected value for a *S* = 5/2 system and might reflect the participation of low-lying intermediate spin state (vide infra). The neutral complexes **6** (μ_eff_ = 4.83 μ_Β_) and **7** (μ_eff_ = 4.82 μ_Β_) exhibit magnetic moments that are in agreement with a high-spin system (*S* = 2, μ_S.O._ = 4.90 μ_Β_). Likewise, the magnetic susceptibilities of the amination products are consistent with high-spin iron­(II) (μ_eff_ = 4.95 μ_B_ (**9**), 4.77 μ_B_ (**10**) and 4.75 μ_B_ (**11**)).

**Table 1:** Important Structural and Electronic Parameters of Mentioned Iron Imido Complexes^a^

R	**compound**	Fe1–N1 (Å)	Fe1–N2 (Å)	Fe1–N3 (Å)	N1–Fe1–N2 (°)	**Fe1–N3–C**_**Ar**_ (°)	**N3–C**_**Ar**_ (Å)	**μ**_**eff**_ (μ_B_)
anionic imides K{crypt.222}[Fe(NR)L_2_]								
Tol	**1**	1.966(3)	1.947(3)	1.765(2)	135.6(2)	175.5(2)	1.355(13)	5.43
				1.775(8)		172.1(3)		
Xyl	**2**	1.947(8)	1.947(8)	1.767(8)	135.2(3)	174.8(7)	1.338(7)	5.62
Dipp	**3**	1.949(2)	1.953(2)	1.777(2)	131.9(9)	173.2(1)	1.341(2)	5.68
Tripp	**4**	1.953(2)	1.972(1)	1.775(2)	128.9(6)	172.4(2)	1.343(2)	4.94
neutral imides [Fe(NR)L_2_]								
Tol	7	1.899(2)	1.894(2)	1.747(2)	133.8(7)	170.9(2)	1.337(3)	4.83
		1.898(2)	1.898(2)	1.746(2)	131.7(7)	172.1(2)	1.334(3)	
Xyl	8	1.896(2)	1.904(2)	1.749(2)	132.6(6)	176.1(2)	1.332(2)	4.82
C–H insertion products								
Ph	**9**	1.908(1)	1.907(1)	2.137(2)	140.1(5)	110.3(8)	1.455(2)	4.95
Tol	**10**	1.922(2)	1.901(2)	2.115(2)	138.9(6)	113.7(2)	1.457(3)	4.77
		1.928(2)	1.921(2)	2.147(2)	149.1(8)	111.2(2)	1.459(3)	
Xyl	**11**	1.915(2)	1.911(2)	2.1202(2)	141.6(8)	110.7(2)	1.456(3)	4.75
		1.924(2)	1.911(2)	2.149(2)	147.3(8)	112.01(14)	1.464(3)	

a Two values for bond/angle represent disorders in the crystal structure (**1**) or two molecules in the unit cell with different bonding parameters (**7**, **10** and **11**).

The tetrazenido complex **6** features a four-coordinate iron center in a distorted tetrahedral geometry, with a principal N1–Fe1–N2 angle value of 128.99(90)° and a N3–Fe1–N6 angle to the tetrazenido unit amounting to 76.29(9)°. The Fe–N_amido_ bond lengths (1.944(2) Å, 1.946(3) Å) are on the lower end, but still in line with the herein reported anionic imido complexes (**1**–**4**). The N–N bond lengths in the tetrazenido unit of **6** exhibit values of 1.357(3) (N3–N4), 1.276(3) (N4–N5) and 1.372(3) Å (N5–N6). Given the known redox-innocence of the tetrazene ligand, comparable bond parameters were observed for either the dianionic^40^ or the radical monoanionic form.^41^ **6** exhibits a magnetic moment of μ_eff_ = 4.96 μ_B_, which is too low for a simple iron­(III) system with a dianionic, diamagnetic tetrazen ligand. As such possible resonance structures of either an iron­(I) tetrazene or an (anti)­ferrom. Coupled iron­(II)/tetrazenido radical anion might be in play (vide infra).

## Computational Analysis

Computational analyses were performed to gather insights into the electronic situation of the imido complexes, the intramolecular amination process and the electronic structure of the tetrazenido complex **6**.

### DFT Analysis of [Fe­(NPh)­L_2_]^−,0^

Geometry optimizations was performed on the elusive [Fe­(NPh)­L_2_]^0,–^, [Fe­(NXyl)­L_2_]^0,–^ and [Fe­(NTripp)­L_2_]^0,–^ by regarding the quartet/sextet state for the anionic and triplet/quintet state for the neutral complexes using the PBE,^42^ PBE0,^43^ and TPSSh^44,45^ functional. Depending on the used functional either the high or the intermediate spin state are predicted as local energetic minima. However, it showed that for all cases only the computed high-spin state geometries align well with the experimentally obtained structural features (see Table S1–S6), while the intermediate spin states are characterized by substantially shorter Fe–N bond lengths. The PBE0 intermediate spin state geometries of the anionic complexes showed some peculiarities, as here the computed Fe–N_imido_ bond lengths were found at unrealistically long 1.8 Å. These discrepancies point to problems for these DFT functionals. No meaningful effect of the steric substitutions was found with regards to the central Fe–NR bond length and Fe–N–R bond angle for the high-spin states. Only for the quartet state a more linear [FeNR] unit is observed for increasingly encumbered imido substituents. Given the overall similar structural metrics, we will focus on the prototypical [Fe­(NPh)­L_2_]^0,–^ for further discussions. The Löwdin population analysis of the sextet state of [Fe­(NPh)­L_2_]^−^ gives a spin population of +3.75 au for Fe and +0.54 au for the imido nitrogen. This would align with a formulation for [Fe­(NPh)­L_2_]^−^ as ferromagnetically coupled iron­(II) imidyl. Löwdin spin densities of the quintet state of [Fe­(NPh)­L_2_]^0^ was computed for Fe to +3.47 au and for N to +0.10 au which hints to a high-spin iron­(II) nitrene character. However, given the fractional spin densities obtained from the DFT methods a more complicated electronic situation might be in play.

### CASSCF/NEVPT2 Analysis of [Fe­(NPh)­L_2_]^−,0^

As such we turned to multireference methods (complete active space self-consistent field (CASSCF) and N-electron valence perturbation theory (NEVPT2))^46,47^ using the TPSSh optimized geometries of the respective high-spin state. As active space the five d-orbitals, the three p_Nimido_ orbitals belonging to the π- and σ-interaction as well as the HOMO/LUMO pair of the aromatic substituent were chosen, giving CAS­(12,10) for [Fe­(NPh)­L_2_]^0^ and CAS­(13,10) for [Fe­(NPh)­L_2_]^−^. For [Fe­(NPh)­L_2_]^0^ the computed NEVPT2 vertical excitation energies showed a clear preference for the high-spin state (Δ*E*_q→t_ = +81.0 kJ/mol). In the main configuration (*c* = 0.66) of the quintet state (Figure 2, left) the π_*xz*_-interaction, which is in-plane with the aromatic substituent itself, is N-centered (Fe:N 0.16:0.64). It is paired with a half-filled Fe-centered antibonding combination (Fe:N 0.87:0.08). In contrast, the perpendicular π_*xy*_/π_xy_*-interaction is very covalent (Fe:N 0.50:0.30 and Fe:N 0.46:0.33) and is occupied by two electrons in the bonding combination. The next two relevant configurations relate to a single (*c* = 0.18) and double (*c* = 0.11) excitation within this orbital manifold. Given the covalency of this π-interaction it eludes a precise description within the imide/imidyl/nitrene trichotomy (Figure 2, right). As such [Fe­(NPh)­L_2_]^0^ is thus better described as {FeNR}^8^ along the Enemark-Feltham notation, while the multiconfigurational character explains the nonfractional DFT results. For [Fe­(NPh)­L_2_]^−^, NEVPT2 predicts isoenergetic quartet and sextet ground states (Δ*E*_sext→quar_ = +1.9 kJ/mol). In the sextet state (Figure 3, left), the HOMO contains an N-centered π-interaction (N:Fe 55:18) which is aligned with the aromatic π-system. The lowest SOMO contains the perpendicular, N-centered π*-interaction (N:Fe 54:30). Together with the six electrons in five d-centered orbitals, it results overall in an iron­(II) imidyl character. In the quartet state of [Fe­(NPh)­L_2_]^−^ the lowest-lying π_*yz*_-interaction is also N-centered (Figure 3, right). The perpendicular π_*xz*_-interaction represents the HOMO, and is correlated with the LUMO (π_xz_*). This π_*xz*_/π_xz_* combination is covalent (Fe:N 50:40 (π), 50:36 (π*)) with the following configurations (*c* = 0.22, 0.17) belonging to the 1e^–^/2e^–^ excitations within this orbital manifold. As such the quartet state of [Fe­(NPh)­L_2_]^−^ is subject to the same imide/imidyl/nitrene interplay as the quintet state of [Fe­(NPh)­L_2_]^0^, and is described accordingly as {FeNR}.^9^ The energetical similarity of the sextet and the quartet state might explain the experimentally determined solution state magnetic susceptibilities that were slightly lower than those expected for a pure sextet. Comparing the orbital situation of [Fe­(NPh)­L_2_]^−,0^ an intrinsic difference between the neutral and anionic imido complex is observed which relates to the orbital containing the N-centered unpaired spin density.In the case of the neutral complex, the N_p_-orbital with mixed imidyl/nitrene/imide character is aligned with the π-system of the aromatic ring. In contrast, the corresponding orbital in the anionic complex is in-plane with the aromatic ring itself with no stabilization via the ring’s π-system. It points toward an intrinsic property of these negatively charged complexes and implies that here partial delocalization of charge and not spin density over the aromatic ring is preferred.

**Figure 2:**
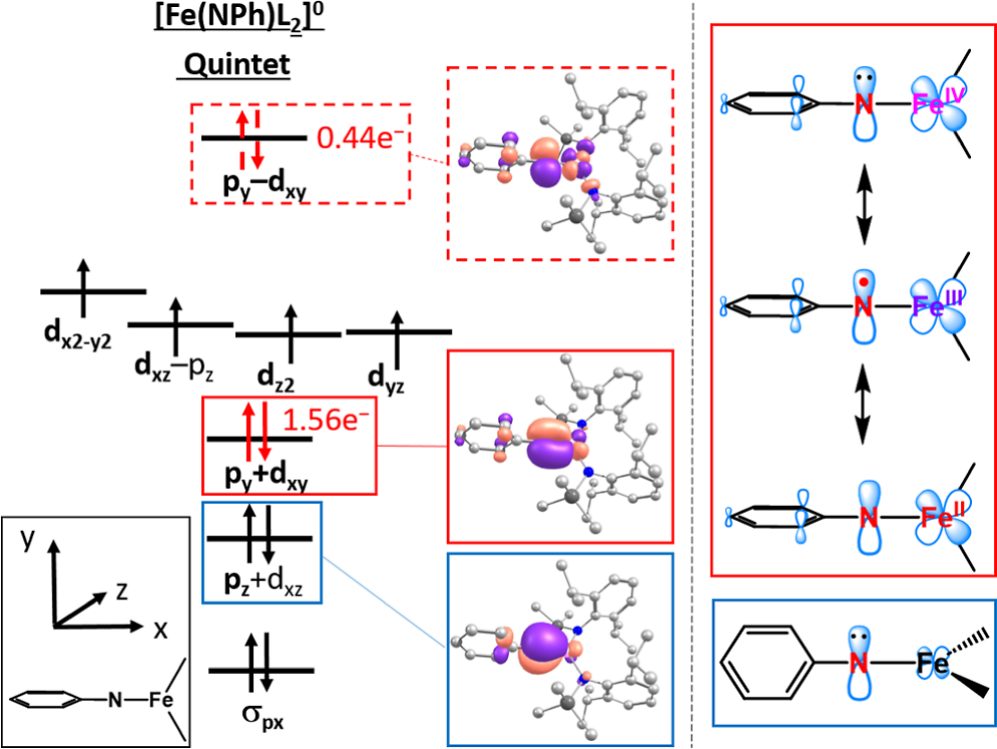
Left: qualitative orbital scheme obtained from single-references CASSCF calculation (CAS­(12,10)) of the quintet state of [Fe­(NPh)­L_2_)]^0^ with important N-centered MO’s. The aromatic HOMO/LUMO pair is omitted for clarity. Right: depiction and relative orientation of the N-centered imido iron interplay.

**Figure 3:**
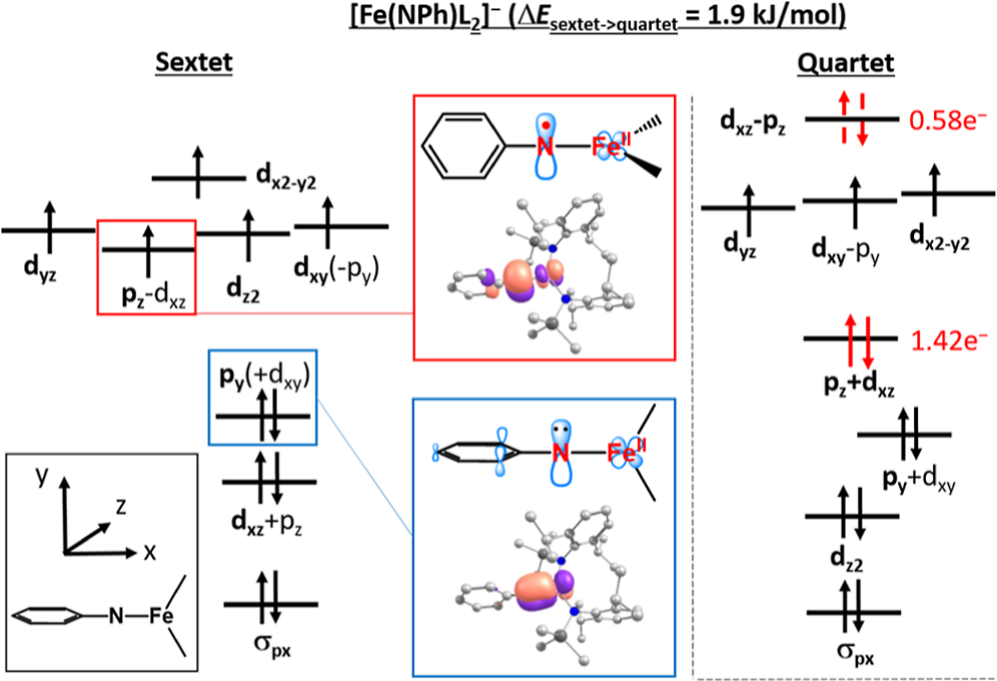
Left: qualitative orbital scheme obtained from single-references CASSCF calculation (CAS­(13,10)) of the sextet state of [Fe­(NPh)­L_2_)]^−^ with important N-centered MO. The aromatic HOMO/LUMO pair is omitted for clarity. Right: qualitative orbital scheme of the quartet state.

### DFT Analysis of the Intramolecular C–H Amination

Next, we wanted to address the observed differences in the intramolecular C–H amination, respectively its absence, for the [Fe­(NPh)­L_2_]^−,0^ system. For that, the possible reaction pathways and intermediates were explored using the PBE functional for geometry optimizations with the single point energies being obtained using the TPSSh functional under inclusion of solvent correction (Et_2_O). Quintet/triplet states are considered for [Fe­(NPh)­L_2_]^0^ and quartet/sextet states for [Fe­(NPh)­L_2_]^−^. For both systems, the amination starts with an initial H atom abstraction, for which the highest barrier is found (Figure 4). Hereby, the transition state is energetically disfavored by 20–40 kJ/mol the anionic complex in comparison to the neutral one ([Fe­(NPh)­L_2_]^0^: 96 (quintet), 102 (triplet) kJ/mol; [Fe­(NPh)­L_2_]^−^: 117 (sextet), 138 (quartet) kJ/mol. We have also assed the impact of steric hindrance in 2,6-position, which gave energy barriers for the HAA process that is energetically further disfavored by 20–40 kJ/mol.

**Figure 4:**
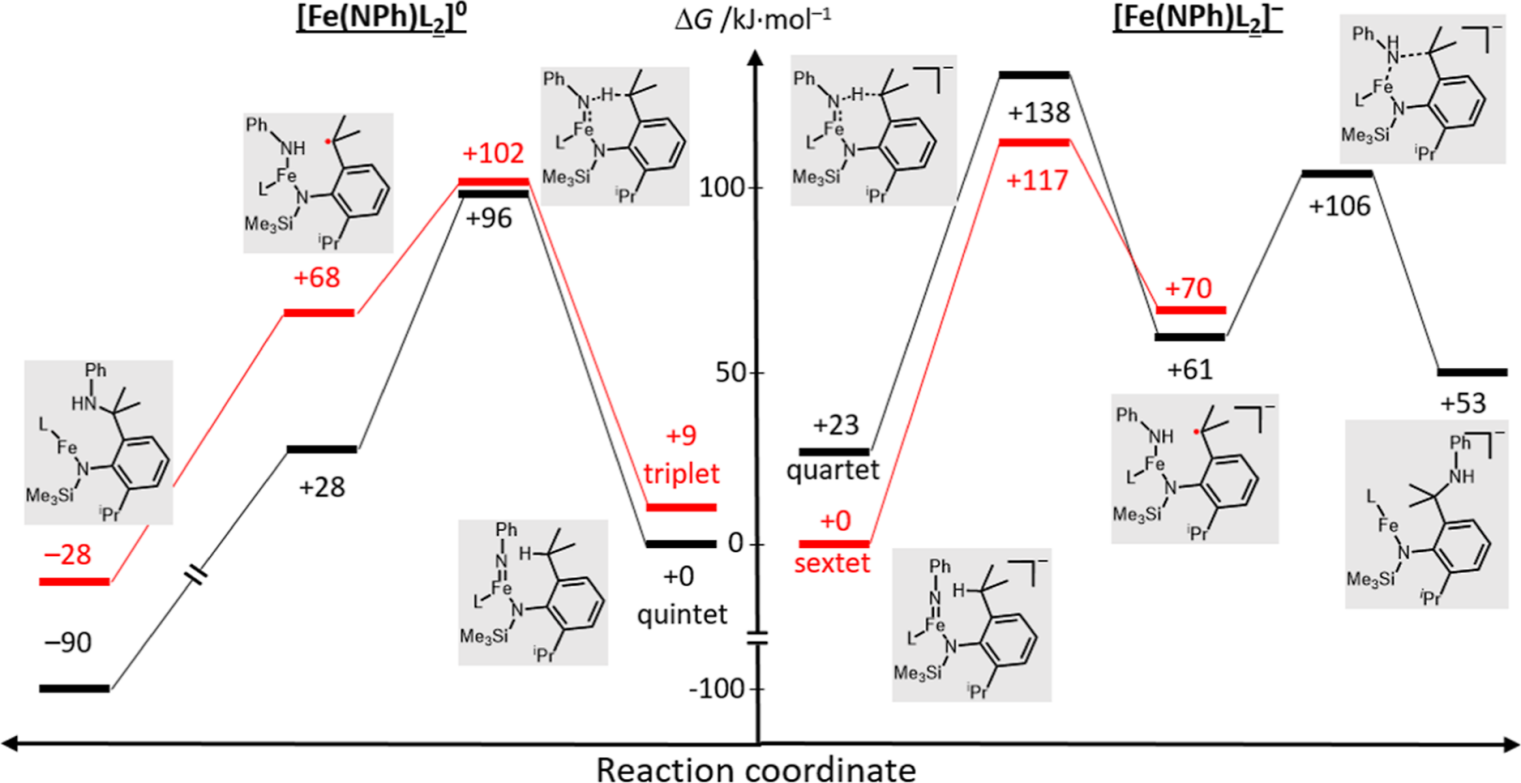
Reaction profile of the intramolecular C–H amination of [Fe­(NPh)­L_2_]^0^ (left) and [Fe­(NPh)­L_2_]^−^ (right) (TPSSh, ZORA-def2-TZVPP, CPCM­(Et_2_O)).

The subsequent C–N bond formation by the formed iron organoradical intermediate of the [Fe­(NPh)­L_2_]^0^ system proceeds without a considerable barrier for the neutral system on the high-spin energy surface. It yielded the amination product in an exergonic manner with regards to [Fe­(NPh)­L_2_]^0^. In contrast, for the anionic system a reasonable transition state is found for the C–N recombination in the intermediate but not the high-spin state. Reaction progression would thus require a spin crossover to the quartet state, where C–N formation is also not barrierless. Furthermore, the subsequent iron­(I) insertion product is endergonic (with regard to the starting imido complex) that reflects on the instability of the oxidation state + I for iron. Overall, it shows that the anionic imido complex is penalized concerning the C–H amination, not only with regards to the initial HAA process but also the overall product formation.

### DFT/CASSCF Analysis of the Tetrazene Complex 6

Finally, we computed the tetrazene complex **6**, with the goal to delineate possible intricacies of the iron/tetrazene interplay.^40,48^ Geometry optimization using DFT methods (PBE, PBE0 and TPSSh functionals) gave congruent features with the experimental solid-state structure only for the sextet state. Löwdin spin densities in the sextet state (given for the PBE functional) imply an iron­(II) ion (3.77 au) with a ferrom. Coupled diazene radical anion (0.68 au equally distributed over the four N atoms). For additional insights we turned to CASSCF/NEVTP2 analysis employing the sextet geometry (PBE functional). The five d-orbitals, the σ-donor as well as the antibonding π*-orbital of the N_4_ backbone of the tetrazene ligand were chosen as the active space (CAS­(9,7)). The inclusion of HOMO/LUMO combinations of the N_4_-backbone did not change the CASSCF transition energies and were therefore excluded due to computational cost. Interestingly, NEVPT2 calculations predicted isoenergetic quartet and sextet ground states (Δ*E*_sext→quar_ = +2.9 kJ/mol), despite the DFT derived sextet state geometry. The main configuration of the quartet state (*c* = 0.49) contains a doubly filled d_*x*2–*y*2_ orbital and a covalent d_*xz*_+π* interaction. This is followed by three singly occupied d_*yz*_, d_z2_ and d_*yx*_ orbitals as well as the empty antibonding d_*xz*_-π* combination. The next two configurations (*c* = 0.27, 0.19) belong to the 1e^–^/2e^–^-excitations within the d_*xz*_ ± π* orbital manifold. As such the electronic structure is described as a valence tautomer between an intermediate-spin iron­(III) with a tetrazene dianion, a high-spin iron­(II) antiferromagn. Coupled to a tetrazene radical anion and a dominant high-spin iron­(I) tetrazene. The isoenergetic sextet exhibits two states that are best described as ferromagnetically coupled iron­(II) tetrazenido radical anions (see Supporting Information). The overall electronic ambiguity might explain the observed magnetic moment of **6**, with a value that is placed between a theoretical quartet and sextet state (see Figure 5).

**Figure 5:**
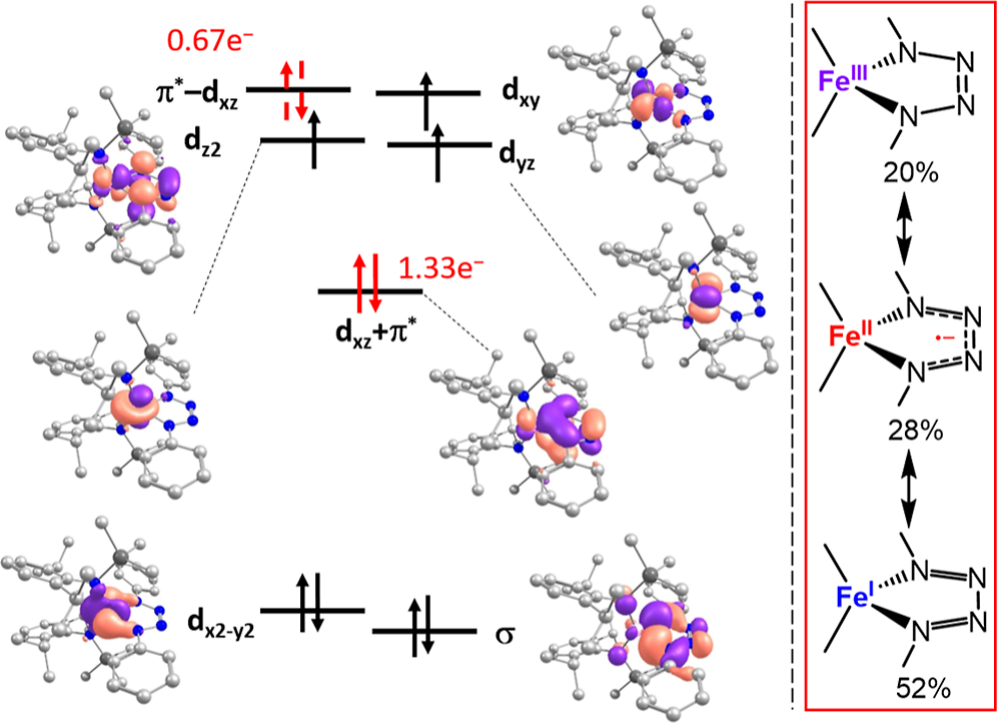
Qualitative orbital scheme obtained from single-references CASSCF calculation (CAS­(9,7)) of the quartet state of [Fe­(N_4_Ph_2_)­L_2_)]^−^ with important N-centered MO’s (left, isovalue of 0.04) and depiction of relevant tetrazene iron resonance structures (right).

## Reactivity with Electro/Nucleophiles

Finally, we wanted to expand on the behavior of obtained imido complexes toward external substrates. Here it showed that reaction of the neutral **7** or **8** with the H atom donor 1,4-cyclohexadiene, PEt_3_ or styrene gave only sluggish reactions, while not interaction with electrophiles (CS_2_, benzaldehyde or Mes-NCO) was observed. Therefore, the reduction of steric encumbrance with regards to [Fe­{NMes}­L_2_] proved unfruitful and is likely perturbed by the competing intramolecular C–H amination. As such we turned to the interaction of the anionic imido iron complexes with electrophiles, given previously reactions from us and Smith with regards to the insertion of CS_2_ or RNCNR into the Fe–NR bond.^15,49^ A precooled solution of **1** in Et_2_O/THF was treated with one equivalent of benzaldehyde to give the metallacycle K­{crypt.222}­[Fe­(N­{Tol}­C­{H}­{Ph}­O)­L_2_], **12**, as dark red crystals (79% yield, Scheme 3). The molecular structure of the anion of **12** shows a four-coordinate iron center with a distorted tetrahedral geometry, a 124.8(2)° angle for the principal N1–Fe1–N2 axis and an acute 70.6(2)° angle for the newly formed four-membered heterocycle (Figure 2). Fe–N_amido_ bond lengths (1.962 Å, 1.971 Å) of **12** do not exhibit substantial changes when compared to the imido precursor **1**. As such, the expected contraction of the metal-amido bonds is counteracted by an increase in coordination number. Under analogous conditions, the reaction of **2** with one equivalent of 2,4,6-trimethylphenyl isocyanate (MesNCO) results in K­{crypt.222}­[Fe­(N­{Xyl}­C­{NMes}­O)­L_2_], **13**, as a violet crystalline solid with a 55% yield (Scheme 3). In solid state, **13** contains a four-coordinate iron center with an angle of 124.9(7)° for N1–Fe1–N2 and 67.5(6)° for O1–Fe1–N3 (Figure 6, right). Fe–N bonds to the amido ligands (1.935(2) Å, 1.932(2) Å), are slightly shorter in **13** than those found in the imido species **2**.

**Scheme 3:**
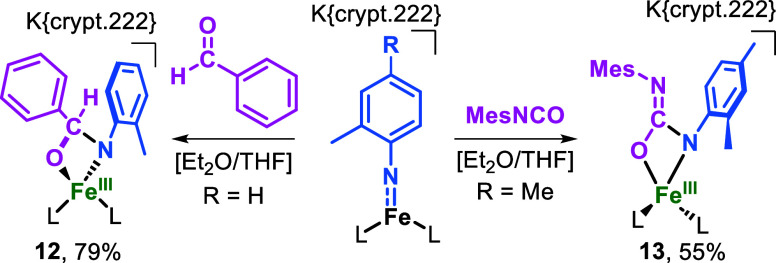
Reaction of **1** with Benzaldehyde, Resulting in the iron­(III) Complex **12** (Above)^a^

a Reaction of **2** with 2,4,6-trimethylphenyl isocyanate, resulting in complex **13**.

**Figure 6:**
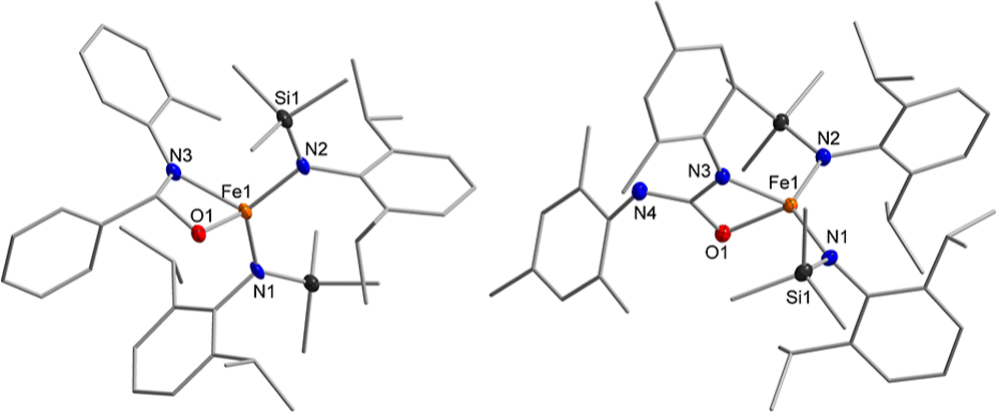
Molecular structure of metallacycles **12** (left) and **13** (right) in the solid state. Ellipsoids are set at 50% probability, hydrogen atoms and cations are hidden for clarity. Selected bond lengths (Å) and angles (°) for **12**: Fe–N1:1.971(3), Fe–N2:1.962(4), Fe–N3:2.011(4), Fe1–O1:1.913(3), N1–Fe1–N2:124.8(2), O1–Fe1–N3:70.6(2). For **13**: Fe–N1:1.935(2), Fe–N2:1.932(2), Fe–N3:2.001(2), Fe1–O1:1.969(2), N1–Fe1–N2:124.9(7), O1–Fe1–N3:67.5(6).

Magnetic susceptibility measurements give for **12** μ_eff_ = 5.89 μ_B_ and for **13** μ_eff_ = 5.96 μ_B_, which aligns with the expectations for a high-spin iron­(III) d^5^ ion (*S* = 5/2). **12** and **13** are the result of a formal [2 + 2] cycloaddition of a carbonyl unit to the Fe–N double bond. This kind of reactivity has been reported for tetra- ^50,51^ and penta-coordinated^52^ titanium imidos, a low-spin cobalt­(II) complex [(Me_3_P)­Co­(NDipp)],^53^ as well as for the reaction of nickel^54^ and chromium^55^ imidos with CO_2_. It speaks for the nucleophilicity of the [Fe = NR] fragment within the anionic complexes. It is tempting to assign this behavior to the above-mentioned N-centered π-interaction, which is orthogonal (angle) to the aromatic ring, and thus more susceptible to an electrophilic attack by the substrate than the in-plane N-centered unpaired spin density.

## Conclusion

In conclusion, we reported a variety of rare trigonal iron aryl imido complexes [Fe­(NR)­L_2_]^−,0^ in high-spin states. They exhibit different steric properties of the imido substituent and are obtained from the reaction of organic azides with the linear iron precursors [FeL_2_]^−,0^. For the anionic complexes, the steric demand ranges from R = Tol to R = Tripp. In the last case, a partial shift of the SiMe_3_ function from an ancillary ligand to the imido function is observed, yielding a new imido metal unit stemming from the coligand. On the other hand, the smallest, prototypical imido iron complex [Fe­(NPh)­L_2_]^−^ was not isolable as it directly reacted with a second organo azide under formation of a tetrazenido complex. The implied nucleophilicity of the anionic imido complexes can be employed for [2 + 2] cycloaddition to a ketone function, as shown for an organo isocyanate or an aldehyde. For neutral imido complexes the steric range was smaller (R = Tol, Xyl, Mes) yet also yielded complexes in higher spin states. The neutral compounds were subject to intramolecular C–H amination that is highly dependent on the steric profile of the imido substituent. No nucleophilic behavior is observed as seen for the anionic complexes. Overall, this work showcased the charge dependent, steric variety of high-spin arylimido complexes, which we currently expand in a systematic fashion to alkyl and other imido substituents. This will potentially allow to navigate between the different (computed) electronic resonance structures and by that invoke different reactivity patterns.

## Experimental Section

### General Procedures

All manipulations were carried out under inert atmosphere of Argon in a glovebox. Solvents were dried by continuous distillation over sodium metal for several days, degassed via three freeze–pump cycles, and stored over 4 Å molecular sieves. ^1^H NMR spectroscopy was conducted using Bruker AV II 300 MHz spectrometers. Elemental analysis measurements were performed using an Elementar vario Micro cube in CHN mode. Infrared spectra were recorded with a Bruker Alpha FT-IR spectrometer. UV/vis spectra were measured using an Analytik-Jena SPECORD S 600 spectrometer. Solution magnetic susceptibilities were determined by the Evans method.^56,57^ 2.2.2-cryptand (crypt.222), and tetramethylsilane (TMS) were obtained commercially if not noted otherwise used as received. K­{18c6}­[FeL_2_] (L = N­(Dipp)­SiMe_3_),^36^ K­{crypt.222}­[FeL_2_],^15^ N_3_Ph,^58^ N_3_Tol,^58^ N_3_Xyl,^58^ N_3_Dipp,^58^ N_3_Tripp,^16^ were prepared according to literature procedures. Liquid azides and TMS were degassed before entering the glovebox. 18c6 was sublimed prior to use. Attention: Organic azides are potentially hazardous and should be handled with care and only in small quantities to avoid explosions!

#### Synthesis of K­{crypt.222}­[Fe­(NTol)­L_2_] (1)

150 mg (0.154 mmol, 1.0 equiv) K­{crypt.222}­[FeL_2_] were dissolved in 3 mL of a Et_2_O/THF mixture at – 30 °C (3:1). Addition of 20.5 mg (0.154 mmol, 1.0 equiv) TolN_3_ at – 30 °C leads to an instantaneous color change from red-brown to dark green with concomitant gas evolution. The solution was stirred for 30 s at room temperature, and stored at – 30 °C. After 1 day, the supernatant was decanted off, the resulting dark green crystals were washed with 2 × 3 mL *n*-pentane. Drying the crystalline solid under reduced pressure yields 135 mg (0.125 mmol, 81%) K­{crypt.222}­[Fe­(NTol)­L_2_]. ^**1**^ **H NMR** (300 MHz, 300 K, thf-*d*_8_, ppm): δ = 51.00 (s, 2H), 12.89 (br s, 9H, – Si­(CH_3_)_3_), 9.50 (br s, 1H, Ar–H), 7.15 (br s, 1H, Ar–H) 5.57 (s, 1H, Ar–H), 3.53 (s, 24H, crypt.222), 2.51 (s, 12H, crypt.222), −48.80 (br s, 1H, Ar–H). **Magnetic susceptibility:** (500 MHz, 300 K, THF-*d*_8_ + 1% TMS) μ_eff_ = 5.43 μ_B_. (μ_s.o._ (*S* = 2.5) = 5.92 μ_B_)_._ **IR:** (ATR, cm^–1^): *v* = 2952­(m), 2884 (m), 1606 (w), 1585 (w), 1487 (m), 1476 (m), 1458 (m), 1445 (m), 1422 (m), 1353 (m), 1313 (m), 1296 (m), 1235 (s), 1191 (m), 1132 (m) 1102 (vs), 1040 (s), 949 (s), 931 (s), 905 (s), 829 (vs), 777 (s), 745 (m), 666 (m), 620 (w), 570 (w), 537 (w), 435 (w). **Elemental analysis:** Calcd (%) for C_55_H_95_FeKN_5_O_6_Si_2_ (1073.51 g mol^–1^): C: 61.54, H: 8.92, N: 6.52; found: C: 61.84, H: 8.76, N: 6.69.

#### Synthesis of K­{crypt.222}­[Fe­(NXyl)­L_2_] (2)

150 mg (0.154 mmol, 1.0 equiv) K­{crypt.222}­[FeL_2_] were dissolved in 3 mL of a Et_2_O/THF mixture at – 30 °C (3:1). Addition of 22.75 mg (0.154 mmol, 1.0 equiv) XylN_3_ at – 30 °C leads to an instantaneous color change from red-brown to dark green with concomitant gas evolution. The solution was stirred for 30 s at room temperature and stored at – 30 °C. After 1 day, the supernatant was decanted off. The resulting dark green crystals were washed with 2 × 3 mL *n*-pentane. Drying the crystalline solid under reduced pressure yielded 123 mg (0.113 mmol, 73%) K­{crypt.222}­[Fe­(NXyl)­L_2_]. ^**1**^ **H NMR** (300 MHz, 300 K, THF-*d*_8,_ ppm): δ = 61.54 (s) 47.73 (br s, 2H), 29.66 (s), 11.45 (br s, 9H, – Si­(CH_3_)_3_), 10.18 (s, 1H, Ar–H), 7.73 (s, 9H, – Si­(CH_3_)_3_), 5.80 (s, 1H, Ar–H), 5.44 (s, 1H, Ar–H) 3.54 (s, crypt.222), 3.50 (s, 12H, crypt.222), 2.51 (s, crypt.222), −43.78 (s, 1H, Ar–H). **Magnetic susceptibility:** (500 MHz, 300 K, THF-*d*_8_ + 1% TMS) μ_eff_ = 5.62 μ_B_. (μ_s.o._ (*S* = 2.5) = 5.92 μ_B_)_._. **IR** (ATR, cm^–1^): *v* = 2946 (m), 2884 (m), 2813 (m), 1611 (w), 1589 (w), 1479 (m), 1461 (w), 1443 (m) 1422 (s), 1352 (s), 1313 (m), 1295 (m), 1234 (s), 1187 (m), 1132 (m), 1104 (vs), 1073 (s), 948 (s), 932 (m), 903 (s), 830 (vs), 778 (s), 741 (m), 666 (m), 619 (w), 572 (w), 540 (w), 525 (w), 433 (w). **Elemental analysis:** Calcd (%) C_56_H_97_FeKN_5_O_6_Si_2_ (1087.53 g·mol^–1^): C: 61.85, H: 8.99, N: 6.44; found: C: 62.18, H: 8.78, N: 6.45.

#### Synthesis of K­{crypt.222}­[Fe­(NDipp)­L_2_] (3)

100 mg (0.103 mmol, 1.0 equiv) K­{crypt.222}­[FeL_2_] were dissolved in 3 mL of a Et_2_O/THF mixture at – 30 °C (3:1). Addition of 21 mg (0.103 mmol, 1.0 equiv) DippN_3_ at – 30 °C led to an instantaneous color change from red-brown to dark green with concomitant gas evolution. The solution was stirred for 5 min at room temperature, layered with *n*-pentane and stored at – 30 °C. After 1 day, the supernatant was decanted off, leaving yellowish-green crystals, which were washed with 2 × 3 mL *n*-pentane. Drying the crystalline solid under reduced pressure yields 92 mg (0.08 mmol, 78%) K­{crypt.222}­[Fe­(NDipp)­L_2_]. ^**1**^ **H NMR** (300 MHz, 300 K, THF-*d*_8_, ppm): δ = 47.35 (br s, 1H), 27.03 (br s, 6H, ^*i*^Pr), 17.34 (br s, 3H, CH_3_(^*i*^Pr)), 9.44 (br s, 1H, Ar–H), 6.94 (br s), 2.57 (s, 12H, crypt.222), −3.06 (br s, 18H, – Si­(CH_3_)_3_z), −27.96 (s), −42.65 (s). **Magnetic susceptibility**: (500 MHz, 300 K, THF-*d*_8_ + 1% TMS) μ_eff_ = 5.68 μ_B_ (μ_s.o._ (*S* = 2.5) = 5.92 μ_B_)_._ **IR** (ATR, cm^–1^): *v* = 3041 (m), 2950 (m), 2882 (m), 2861 (w), 2815 (m), 1584 (w), 1576 (w), 1477 (m), 1458 (m), 1445 (m), 1422 (m), 1403 (s), 1377 (m), 1354 (m), 1312 (m), 1297 (vs), 1235 (s), 1190 (m), 1132 (m), 1102 (vs), 949 (vs), 931 (s), 901 (s), 880 (m), 829 (vs), 777 (vs), 751 (s), 734 (m), 673 (w), 625 (w), 572 (w), 540 (m), 523 (w), 436 (w). **Elemental analysis:** Calcd (%) C_60_H_105_FeKN_5_O_6_Si_2_ (1143.64 g·mol^–1^) C: 63.01, H: 9.25, N: 6.12; found: C: 63.12, H: 9.19, N: 5.91.

#### Synthesis of K­{crypt.222}­[Fe­(NTripp)­L_2_] (4)

50 mg (0.051 mmol, 1.0 equiv) K­{crypt.222}­[FeL_2_] were dissolved in 3 mL of a Et_2_O/THF mixture at – 30 °C (3:1). Addition of 12.7 mg (0.051 mmol, 1.0 equiv) TrippN_3_ at – 30 °C led to an immediate color change from red-brown to dark green with concomitant gas evolution. The solution was stirred for 5 min at room temperature and the solvent was removed. The residue was taken in 1,2-difluorobenzene, layered with *n*-pentane and stored at – 30 °C. After 1 day, the supernatant was decanted off, leaving dark green crystals, which were washed with 2 × 3 mL *n*-pentane. Drying the crystalline solid under reduced pressure yielded 47.8 mg (0.04 mmol, 79%) K­{crypt.222}­[Fe­(NTripp)­L_2_]. ^**1**^ **H NMR** (300 MHz, 300 K, thf-*d*_8_, ppm): δ = 38.62 (br s, 2H), 14.40 (s, 12H, ^*i*^Pr), 7.12 (*m*), 3.55 (s, 12H, crypt.222), 3.52 (s, 12H, crypt.222), 2.53 (s, 12H, crypt.222), 2.34 (br s, 4H, ^*i*^Pr), −7.23 (s, 18H, – Si­(CH_3_)_3_), −27.98 (s, 1H, Ar–H). **Magnetic susceptibility:** (500 MHz, 300 K, THF-*d*_8_ + 1% TMS) μ_eff_ = 4.94 μ_B_ (μ_s.o._ (*S* = 2.5) = 5.92 μ_B_). **IR** (ATR, cm^–1^): *v* = 3049 (vw), 2957 (m), 2895 (m), 2866 (m), 1589 (w), 1475 (m), 1454 (m), 1420 (m), 1380 (w), 1351 (m), 1307 (w), 1271 (s), 1244 (s), 1228 (s), 1175 (s), 1146 (vw), 1107 (vs), 1081 (m), 1065 (m), 1020 (vs), 993 (s), 976 (m), 959 (s), 929 (w), 899 (w), 835 (m), 780 (s), 754 (vs), 735 (s), 725 (s), 694 (s), 675 (m), 625 (w), 590 (w), 574 (w), 540 (m), 527 (m), 460 (m), 447 (m), 433 (m). **Elemental analysis:** Calcd (%) C_63_H_111_FeKN_5_O_6_Si_2_ (1185.69 g·mol^–1^) accounting for 0.5 molecules of 1,2-difluorobenzene: C: 63.78, H: 9.16, N: 5.63; found: C: 63.56, H: 8.73, N: 5.94.

#### Synthesis of K­{crypt.222}­[Fe­(NDipp)­(N­{Tripp}­SiMe_3_)­L] (5)

80 mg (0.08 mmol, 1.0 equiv) K­{crypt.222}­[FeL_2_] were dissolved in 3 mL of a Et_2_O/THF mature at – 30 °C (3:1). Addition of 21 mg (0.08 mmol, 1.0 equiv) TrippN_3_ at – 30 °C led to an instantaneous color change from red-brown to dark green with concomitant gas evolution. The solution was stirred for 5 min at ambient temperatures, layered with 2 mL *n*-pentane and stored at – 30 °C for 2 weeks leading to the formation of brown crystals. The supernatant was decanted off, leaving brown crystals that were washed with 2 × 3 mL *n*-pentane. Drying of the crystalline solid under reduced pressure yielded K­{crypt.222}­[Fe­(NDipp)­(N­{Tripp}­SiMe_3_)­L] (**5**) The ^1^H NMR spectrum of **5** shows the presence of characteristic signals corresponding to compound **4**, indicating an incomplete transformation. For this reason, further analytical characterization of the sample was not conducted. ^**1**^ **H NMR:** (300 MHz, 300 K, THF-*d*_8_): δ = 44.25 (s), 29.35 (br s), 28.44 (br s), 23.92 (s), 18.31 (s), 14.42 (s), 6.81 (s), 3.40 (s, crypt.222), 2.42 (s, crypt.222), −2.82 (s), −7.23 (s), −28.03 (s), −43.66 (s).

#### Synthesis of K­{18c6}­[Fe­(η^2^-κN^1^:κN^4^–N_4_Ph_2_)­L_2_] (6)

50 mg (0.058 mmol, 1.0 equiv) K­{18c6}­[FeL_2_] was dissolved in 3 mL of a Et_2_O/THF mixture at – 30 °C (3:1). Addition to 15.3 mg (0.127 mmol, 2.2 equiv) PhN_3_ at – 30 °C led to an instantaneous color change from bronze to dark red. The solution was stirred for 3 min, layered with 2 mL *n*-pentane and stored at – 30 °C. After several days the supernatant was removed using a Pasteur pipet, leaving dark red crystals, that were washed with 3 × 3 mL *n*-pentane. Drying of the crystalline solid under reduced pressure yielded 24 mg (0.023 mmol, 49%) K­{18c6}­[Fe­(η^2^-κN:^1^ κN^4^-N_4_Ph_2_)­L_2_]. ^**1**^ **H NMR** (300 MHz, 300 K, THF-*d*_8_): δ = 77.15 (s), 68.41 (s), 11.32 (s), 5.85 (s), 3.74 (s, 18c6), −4.22 (s), −56.56 (s). **Magnetic susceptibility:** (500 MHz, 300 K, THF-*d*_8_ + 1% TMS) μ_eff_ = 4.96 μ_B_. (μ_s.o._ (*S* = 2) = 4.90 μ_B_). **IR** (ATR, cm^–1^): *v* = 3051 (w), 2898 (m), 2866 (w), 2073 (w), 2006 (w), 1588 (m), 1475 (m), 1419 (m), 1380 (m), 1351 (m), 1307 (m), 1271 (m), 1244 (m), 1227 (m), 1175 (m), 1147 (m), 1107 (vs), 1080 (w), 1019 (w), 993 (m), 959 (m), 928 (m), 886 (m), 867 (m), 834 (vs), 780 (m), 754 (m), 734 (m), 725 (m), 694­(m), 674 (m), 540 (w), 528 (w), 445 (w) m–1. **Elemental analysis:** Calcd (%) for C_54_H_86_FeKN_6_O_6_Si_2_ (1066.4 g mol^–1^) accounting for one THF molecule: C: 61.19, H: 8.32, N: 7.39; found: C: 61.22, H: 8.40, N: 7.15.

#### Synthesis of [Fe­(NTol)­L_2_] (7)

100 mg [FeL_2_] (0.181 mmol, 1 equiv) were dissolved in *n*-pentane and cooled down to −30 °C. N_3_Tolyl (24.1 mg, 0.181 mmol, 1 equiv) was added to the cold solution, resulting in a rapid color change from bright orange to dark brown with strong gas evolution. The reaction mixture was then allowed to release gas for 3 min at room temperature. After concentrating the solution to a minimal amount of solvent, it was stored at – 30 °C over 48 h, resulting in dark orange/brown crystals. Drying the crystalline solid under reduced pressure yields 79 mg (0.120 mmol, 66%) [Fe­(NTol)­L_2_]. ^1^H NMR spectroscopy measurements result in a large number of signals throughout the diamagnetic and paramagnetic regions. **Magnetic susceptibility:** (500 MHz, 300 K, C_6_D_6_ + 1% TMS) μ_eff_ = 4.83 μ_B_. (μ_s.o._ (*S* = 2) = 4.89 μ_B_)_._ **IR:** (ATR, cm^–1^): *v* = 3048 (w), 2953 (s), 2865 (m), 1586 (w), 1570 (w) 1453 (m), 1423 (m), 1382 (w), 1360 (w), 1310 (m), 1286 (w), 1241 (s), 1182 (s), 1151 (m), 1102 (m), 1050 (m), 1040 (m), 914 (m), 890 (m), 871 (m), 828 (vs), 782 (vs), 761 (vs), 747 (vs), 729 (vs), 702 (m), 676 (s), 625 (m), 552 (vw), 534 (m), 508 (w), 465 (w), 440 (vw), 428 (s). **Elemental analysis:** Calcd (%) for C_37_H_59_FeKN_3_Si_2_ (657.91 g mol^–1^): C: 67.55, H: 9.04, N: 6.39; found: C: 67.55, H: 8.69, N: 6.73.

#### Synthesis of [Fe­(NXyl)­L_2_] (8)

100 mg [FeL_2_] (0.181 mmol, 1 equiv) were dissolved in *n*-pentane and cooled down to −30 °C. N_3_Xyl (26.6 mg, 0.181 mmol, 1 equiv) was added to the cold solution, resulting in a rapid color change from bright orange to dark brown with strong gas evolution. The reaction mixture was then allowed to release gas for 3 min at room temperature. After concentrating the solution to a minimal amount of solvent, it was stored at – 30 °C over 48 h, resulting in dark orange/brown crystals. Drying the crystalline solid under reduced pressure yields 72 mg (0.107 mmol, 59%) [Fe­(NTol)­L_2_]. ^1^H NMR spectroscopy measurements result in a large number of signals throughout the diamagnetic and paramagnetic regions. **Magnetic susceptibility:** (500 MHz, 300 K, C_6_D_6_ + 1% TMS) μ_eff_ = 4.82 μ_B_. (μ_s.o._ (*S* = 2) = 4.89 μ_B_)_._ **IR:** (ATR, cm^–1^): *v* = 3046 (w), 2955, 2865 (m), 1581 (m), 1453 (m), 1424 (m), 1381 (w), 1359 (m), 1241 (s), 1211 (m), 1182 (s), 1147 (m), 1121 (w), 1101 (m), 1050 (w), 1038 (w), 931 (vw), 891 (m), 871 (m), 829 (vs), 783 (vs), 746 (s), 729 (vs), 696 (m), 675 (s), 629 (m), 559 (w), 534 (s), 479 (vw), 431 (s). **Elemental analysis:** Calcd (%) C_38_H_61_FeKN_3_Si_2_ (671.94 g·mol^–1^): C: 67.93, H: 9.15, N: 6.25; found: C: 68.15, H: 9.24, N: 6.40.

#### Synthesis of [Fe­(κ^2^-N-N­(SiMe_3_)-2-(CH_3_)_2_CNH­{Ph}-6-^*i*^Pr-Phenyl)­L] (9)

100 mg [FeL_2_] (0.181 mmol, 1 equiv) were dissolved in *n*-pentane and cooled down to −30 °C. A solution of N_3_Ph (21.55 mg, 0.181 mmol, 1 equiv) in *n*-pentane was added to the solution, resulting in a rapid color change from bright orange to purple with a strong gas evolution. The reaction mixture released gas for 3 min at room temperature. After concentrating the solution to a minimal amount of solvent, it was stored at – 30 °C overnight. This resulted in pale-yellow crystals. Drying the crystalline solid under reduced pressure yields 35 mg (0.054 mmol, 30%) of [Fe­(κ^2^-N-N­(SiMe_3_)-2-(CH_3_)_2_CNH­{Ph}-6-^*i*^Pr-phenyl)­L]. ^1^H NMR spectroscopy measurements result in a large number of signals throughout the diamagnetic and paramagnetic regions. **Magnetic susceptibility:** (500 MHz, 300 K, C_6_D_6_ + 1% TMS) μ_eff_ = 4.95 μ_B_. (μ_s.o._ (*S* = 2) = 4.89 μ_B_)_._ **IR:** (ATR, cm^–1^): *v* = 3269 (vw), 3058 (vw), 2950 (m), 2884 (w), 1586 (w), 1495 (w), 1456 (w), 1416 (s), 1381 (m), 1360 (w), 1313 (m), 1239 (s), 1208 (m), 1192 (m), 1156 (w), 1141 (m), 1112 (m), 1048 (w), 1029 (w), 1015 (w), 1000 (w), 954 (w), 916 (vs), 885 (vs), 829 (vs), 781 (s), 745 (s), 682 (s), 670 (m), 623 (w), 602 (m), 586 (m), 565 (m), 543 (w), 525 (m), 509 (m), 497 (w), 462 (w), 433 (m). **Elemental analysis:** Calcd (%) for C_36_H_57_FeN_3_Si_2_ (643.89 g mol^–1^): C: 67.15, H: 8.92, N: 6.53; found: C: 67.11, H: 8.75, N: 7.01.

#### **Synthesis of [Fe­(κ** ^2^-N-N­(SiMe_3_)-2-(CH_3_)_2_CNH­{Tol}-6-^*i*^Pr-Phenyl)­L] (10)

100 mg [FeL_2_] (0.181 mmol, 1 equiv) were dissolved in *n*-heptane and cooled down to −30 °C. A solution of N_3_Tol (24.1 mg, 0.181 mmol, 1 equiv) in *n*-heptane was added to the solution, resulting in a rapid color change from bright to dark orange with strong gas evolution. The solvent was removed and the residue was taken into *n*-heptane and heated at 80 °C for 2 h. Removing the solvent and dissolving the residue in a concentrated HMDSO solution resulted in pale-yellow crystals after several days at – 30 °C. Drying the crystalline solid under reduced pressure yields 5 mg (0.007 mmol, 4%) of [Fe­(κ^2^-N-N­(SiMe_3_)-2-(CH_3_)_2_CNH­{Tol}-6-^*i*^Pr-phenyl)­L]. ^1^H NMR spectroscopy measurements result in a large number of signals throughout the diamagnetic and paramagnetic regions. **Magnetic susceptibility:** (500 MHz, 300 K, C_6_D_6_ + 1% TMS) μ_eff_ = 4.77 μ_B_. (μ_s.o._ (*S* = 2) = 4.89 μ_B_)_._ **IR:** (ATR, cm^–1^): *v* = 3272 (vw), 3053 (vw), 2952 (m), 2866 (w), 1585 (w), 1489 (w), 1457 (w), 1414 (s), 1382 (m), 1360 (m), 1312 (m), 1237 (s), 1218 (m), 1189 (m), 1158 (m), 1143 (m), 1113 (m), 1102 (m), 1046 (m), 989 (w), 953 (w), 909 (s), 833 (vs), 779 (vs), 742 (s), 713 (m), 665 (m), 622 (w), 599 (w), 541 (w), 522 (m), 500 (w), 468 (w), 429 (m). **Elemental analysis:** Calcd (%) for C_37_H_59_FeN_3_Si_2_ (657.91 g mol^–1^): C: 67.55, H: 9.04, N: 6.39; found: C: 67.82, H: 9.20, N: 6.89.

#### Synthesis of [Fe­(κ^2^-*N*-N­(SiMe_3_)-2-(CH_3_)_2_CNH­{Xyl}-6-^*i*^Pr-Phenyl)­L] (11)

100 mg [FeL_2_] (0.181 mmol, 1 equiv) were dissolved in *n*-pentane and cooled down to −30 °C. A solution of N_3_Xyl (26.6 mg, 0.181 mmol, 1 equiv) in *n*-heptane was added to the solution, resulting in a rapid color change from bright to dark orange with strong gas evolution. The solvent was removed and the residue was taken into *n*-heptane and heated at 80 °C for 2 h. Removing the solvent and dissolving the residue in a concentrated HMDSO solution resulted in pale-yellow crystals after several days at – 30 °C. Drying the crystalline solid under reduced pressure yields 37 mg (0.055 mmol, 30%) of [Fe­(κ^2^-N-N­(SiMe_3_)-2-(CH_3_)_2_CNH­{Xyl}-6-^*i*^Pr-phenyl)­L]. ^1^H NMR spectroscopy measurements result in a large number of signals throughout the diamagnetic and paramagnetic regions. **Magnetic susceptibility:** (500 MHz, 300 K, C_6_D_6_ + 1% TMS) μ_eff_ = 4.75 μ_B_. (μ_s.o._ (*S* = 2) = 4.89 μ_B_)_._ **IR:** (ATR, cm^–1^): *v* = 3056 (vw), 2952 (m), 2867 (w), 1586 (w), 1499 (w), 1457 (w), 1415 (m), 1382 (m), 1359 (m), 1326 (m), 1313 (m), 1242 (s), 1207 (m), 1189 (m), 1157 (w), 1140 (w), 1115 (m), 1041 (w), 1014 (vw), 997 (vw), 910 (vs), 884 (m), 832 (vs), 782 (vs), 744 (s), 669 (m), 625 (w), 603 (w), 576 (w), 567 (w), 538 (m), 528 (m), 506 (w), 434 (m). **Elemental analysis:** Calcd (%) for C_37_H_59_FeN_3_Si_2_ (671.94 g mol^–1^): C: 67.93, H: 9.15, N: 6.25; found: C: 67.82, H: 8.82, N: 6.71.

#### Synthesis of K­{crypt.222}­[Fe­(N­{Tol}­C­{H}­{Ph}­O)­L_2_] (12)

50 mg (0.046 mmol, 1.0 equiv) K­{crypt.222}­[Fe­(NTol)­L_2_] were dissolved in 3 mL of a Et_2_O/THF mixture at – 30 °C (3:1). Addition of 5.5 mg (0.046 mmol, 1.0 equiv) benzaldehyde at – 30 °C leads to a color change from dark green to dark blue over 10 min. After further stirring at room temperature for 10 min, the mixture was filtered, layered with *n*-pentane and stored at – 30 °C overnight. The supernatant was decanted off and the resulting rust-colored crystals were washed with 2 × 3 mL *n*-pentane. Drying the crystalline solid under reduced pressure yields 43 mg (0.036 mmol, 79%) K­{crypt.222}­[Fe­(N­{Tol}­C­{H}­{Ph}­O)­L_2_]. ^**1**^ **H NMR** (300 MHz, 300 K, THF-*d*_8_, ppm): δ = 18.51 (br s), 9.57 (s), 6.98 (br s), 2.53 (s). **Magnetic susceptibility:** (500 MHz, 300 K, THF-*d*_8_ + 1% TMS) μ_eff_ = 5.89 μ_B_. (μ_s.o._ (*S* = 2.5) = 5.92 μ_B_)_._ **IR:** (ATR, cm^–1^): *v* = 3048 (w), 2957 (m), 2884 (m), 2815 (m), 2763 (w), 2693 (vw), 1587 (m), 1478 (m), 1449 (m), 1422 (m), 1379 (w), 1355 (m), 1301 (w), 1254 (m), 1233 (s), 1172 (m), 1132 (s), 1099 (vs), 1052 (m), 1041 (s), 1022 (m), 983 (w), 946 (s), 923 (m), 891 (s), 873 (s), 830 (vs), 777 (vs), 742 (s), 703 (s), 664 (s), 643 (m), 621 (m), 610 (m), 571 (w), 530 (s), 503 (w), 436 (s). **Elemental analysis:** Calcd (%) for C_62_H_101_FeKN_5_O_7_Si_2_ (1179.6 g mol^–1^): C: 63.13, H: 8.63, N: 5.94; found: C: 62.86, H: 8.38, N: 6.35.

#### Synthesis of K­{crypt.222}­[Fe­(N­{Xyl}­C­{NMes}­O)­L_2_] (13)

50 mg (0.051 mmol, 1.0 equiv) K­{crypt.222}­[Fe­(NXyl)­L_2_] were dissolved in 3 mL of a Et_2_O/THF mixture at – 30 °C (3:1). Addition of 8.3 mg (0.051 mmol, 1.0 equiv) MesNCO at – 30 °C leads to a gradual color change from dark green to red-brown over 1 h. After further stirring at room temperature for 1 h, the mixture was filtered, layered with *n*-pentane and stored at – 30 °C. After several days, the supernatant was decanted off and the resulting red-brown crystals were washed with 2 × 3 mL *n*-pentane. Drying the crystalline solid under reduced pressure yields 38 mg (0.028 mmol, 55%) K­{crypt.222}­[FeN­{Xyl}­C­{NMes}­O)­L_2_]. ^**1**^ **H NMR** (300 MHz, 300 K, THF-*d*_8_, ppm): δ = 18.51 (br s), 9.57 (s), 6.98 (br s), 2.53 (s). **Magnetic susceptibility:** (500 MHz, 300 K, THF-*d*_8_ + 1% TMS) μ_eff_ = 5.96 μ_B_. (μ_s.o._ (*S* = 2.5) = 5.92 μ_B_)_._ **IR:** (ATR, cm^–1^): *v* = 3050 (vw), 2958 (m), 2889 (m), 2865 (m), 2818 (w), 1611 (w), 1543 (s), 1477 (m), 1459 (m), 1444 (m), 1424 (m), 1378 (w), 1354 (m), 1294 (m), 1236 (s), 1204 (m), 1179 (m), 1133 (m) 1102 (vs), 1080 (s), 1042 (m), 993 (m), 950 (s), 933 (m), 890 (m), 869 (s), 833 (vs), 780 (vs), 751 (m), 727 (s), 691 (m) 675 (m), 610 (w), 571 (m) 538 (m), 472 (vw), 431 (s). **Elemental analysis:** Calcd (%) for C_66_H_108_FeKN_6_O_7_Si_2_ (1320.9 g mol^–1^): C: 63.48, H: 8.72, N: 6.73; found: C: 63.58, H: 8.63, N: 6.75.

## Computational Section

The calculations were performed with ORCA v. 5.0.4.^59–62^ For visual representation Chemcraft v. 1.8 was used.^63^

### Imido and Amido Complexes

Structure optimization for [Fe­(NPh)­L_2_]^−,0^, [Fe­(NXyl)­L_2_]^−,0^, [Fe­(NTripp)­L_2_]^−,0^, (Tripp = 2,4,6-^*i*^Pr_3_-C_6_H_2_), [Fe­(N_4_Ph_2_)­L_2_]^−^ (**6** ^–^) and the insertion products **9** ^**–,0**^ were performed using the solid-state structures (if available) or constructed using closely resembling isolated complexes. Geometry optimization for the anionic complexes was performed for the sextet and quartet state, and for the neutral complexes for the quintet and triplet state. For the geometry optimization the PBE^42^ functional with the ZORA-def2-SVP basis set (def2-TZVP on Fe) and the D4 ^64^ dispersion was employed for all complexes. The structures of [Fe­(NPh)­L_2_]^−,0^, **9** ^**–,0**^ and **6** ^–^ were further benchmarked using the PBE0 ^43^ and TPSSh^44,45^ functionals. All optimized structures were verified as true minima by the absence (*N* ^imag^ = 0) of negative eigenvalues in the harmonic vibrational frequency analysis using the same options as for the geometry optimizations. Single point calculations were performed for the PBE, PBE0, and TPSSh functionals using the ZORA-def2-TZVPP^65^ basis set for all atoms where scalar relativistic effects were modeled under the Zeroth Order Regular Approximation (ZORA).^66^ The RI approximation with the related auxiliary basis sets (SARC/*J*)^67^ was used to reduce computation time of calculations using the GGA functionals.

CASSCF/NEVPT2 calculations on the imido complexes were carried out at the triple-ζ level of theory (ZORA-def2-TZVPP, SARC/J, autoaux, RIJCOSX).^46,47^ For the imido complexes [Fe­(NPh)­L_2_]^−,0^ the active space was chosen as to include the five d-orbitals, the two imido π-orbitals, the imido σ-orbitaland the two orbitals corresponding to the HOMO/LUMO pair of the aromatic ring, to give an active space of CAS­(13,10) ([Fe­(NPh)­L_2_]^−^) and CAS­(12,10) ([Fe­(NPh)­L_2_]^0^). For **6** ^**–**^ the active space (CAS­(9,7)) contains the five d-orbitals, the antibonding π-orbital of the N_4_-backbone as well as the highest σ-donor orbital of the tetrazenido ligand. The electronic structure analysis in the manuscript relates to calculations without state averaging, whereas state-averaging was applied to determine the energies of vertically excited states.

### Transition States and Reaction Profiles

The transition states of a reaction were first approximated using a relaxed geometry scan along the respective coordinate using the PBE functional and a doublet or a quartet state. The transition state was optimized by calculating the exact Hessian (ZORA-def2-SVP basis set (def2-TZVP on Fe), D4 dispersion correction). For calculation of the energy profile the Gibbs free energy of the transition state, the geometry of all states was obtained by using the PBE functional. Single point energies were obtained using the TPSSh functional (ZORA-def2-TZVPP bass set for all atoms) with solvent correction (CPCM­(Et_2_O)) and adjusted by the entropy contributions from the frequency calculations (PBE functional).

## Supplementary Material



## Notes

^1^H NMR, IR, UV/vis spectra, crystallographic details, coordinates and results from quantum chemical calculations (PDF)
