# Supplementary material for: A Series of Trigonal Arylimido Iron Complexes and the Influence of Oxidation State and Steric Demand on Reactivity
Source: Inorg Chem. 2025 Nov 7;64(46):22604–14. doi: 10.1021/acs.inorgchem.5c03148 (PMC12648643; doi:10.1021/acs.inorgchem.5c03148)
Supplement: Supplementary file 1 [file ic5c03148_si_001.pdf]

Supporting Information

A Series of Trigonal Arylimido Iron Complexes and the Influence of Oxidation State and Steric Demand on Reactivity

Andres Gonzalez,[a] Sascha Reith,[a] Alexander Reckziegel,[a] C. Gunnar Werncke[*,a,b]

a) Philipps-University Marburg, Hans-Meerwein-Straße 4, D-35032 Marburg, Germany

b) Leipzig University, Johannisallee 29, D-04103 Leipzig, Germany

*E-Mail: gunnar.werncke@chemie.uni-leipzig.de

Table of Contents

1. Spectra of Compounds 1–13 .... S1
2. Computational Details S18
3. Details of X-Ray diffraction analysis, refinement and molecular structures.. S48

1. Spectra of Compounds 1–13

Compound K{crypt.222}[Fe(NTol)L$_2$], 1

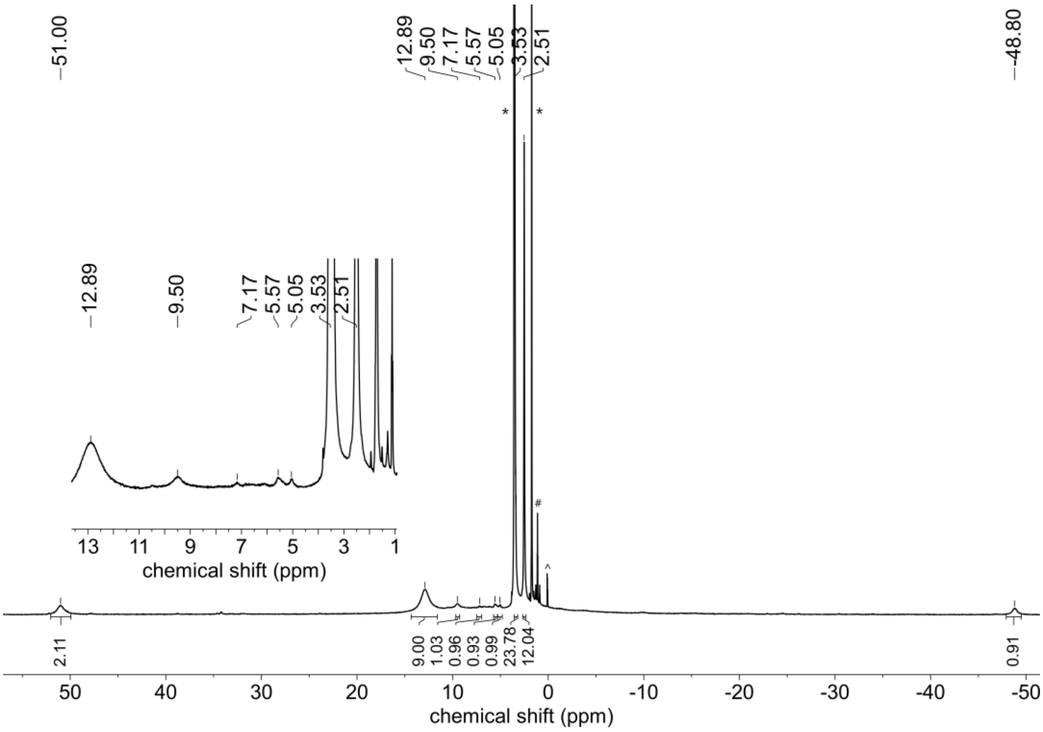

Figure S 1. ^{1}H-NMR spectrum (300 Mhz, 300 K, thf-d$_8$) of K{crypt.222}[Fe(NTol)L$_2$], **1** (* thf-d$_8$, # diethyl ether, ^ decomposition).

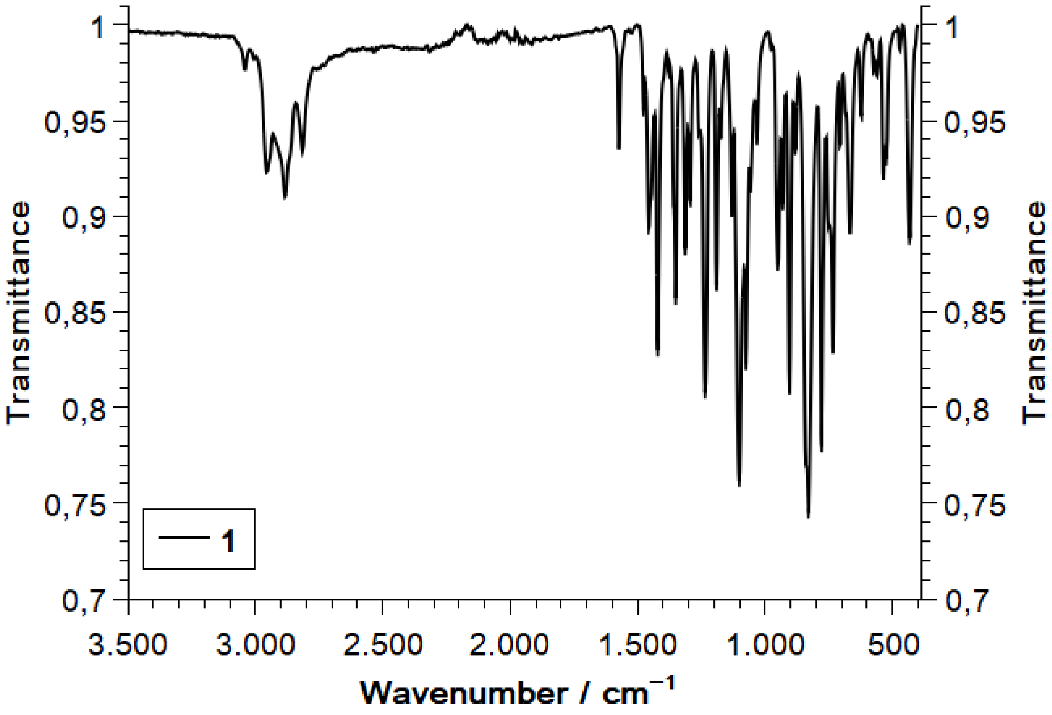

Figure S 2. ATR IR spectrum of solid K{crypt.222}[Fe(NTol)L$_2$], **1**.

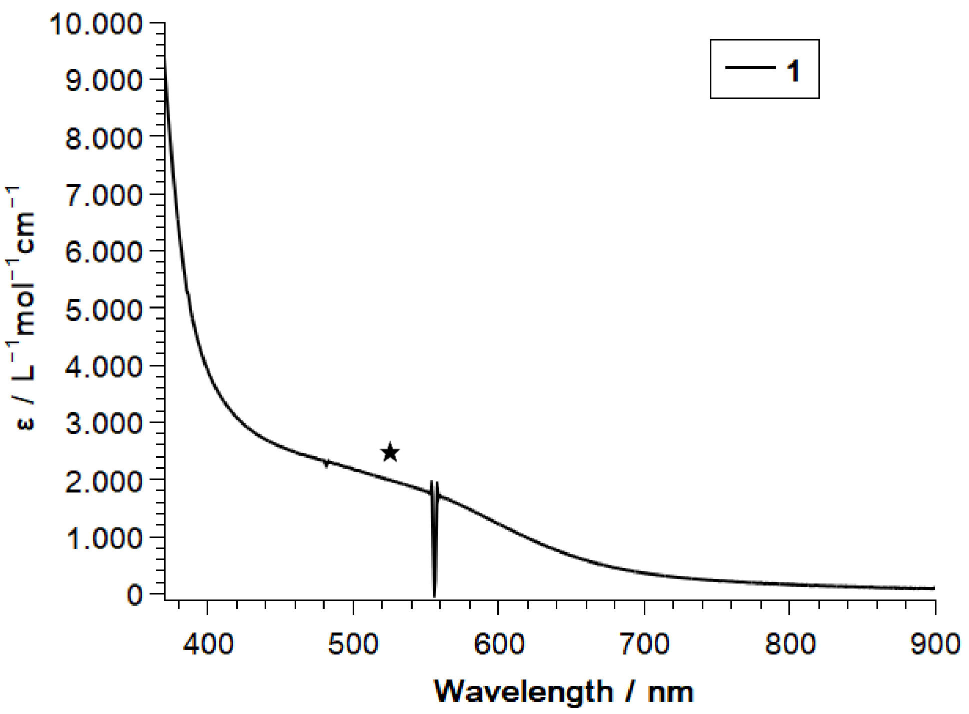

Figure S 3. UV-Vis-NIR spectrum of K{crypt.222}[Fe(NTol)L_2], **1**, in thf. The ★ symbol corresponds to an artifact caused by the UV-Vis device.

Compound K{crypt.222}[Fe(NXyl)L_2], 2

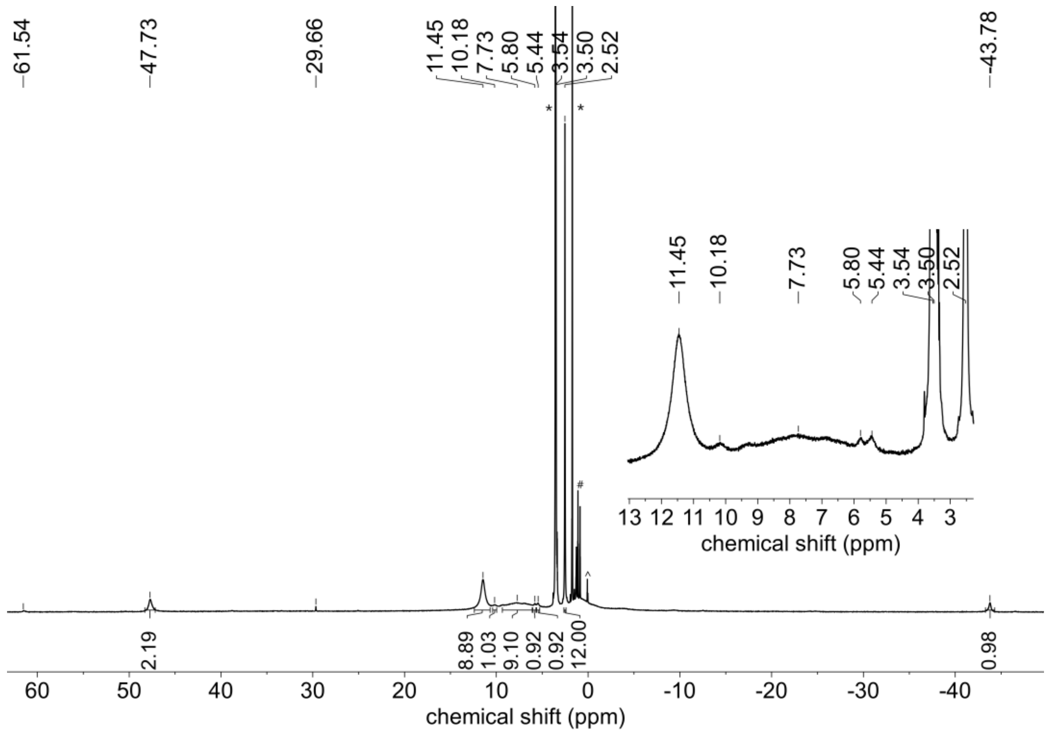

Figure S 4. ^{1}H-NMR spectrum (300 Mhz, 300 K, thf-d_8) of K{crypt.222}[Fe(NXyl)L_2], **2** (* thf-d_8, ° *n*-pentane, # diethyl ether, ^ decomposition).

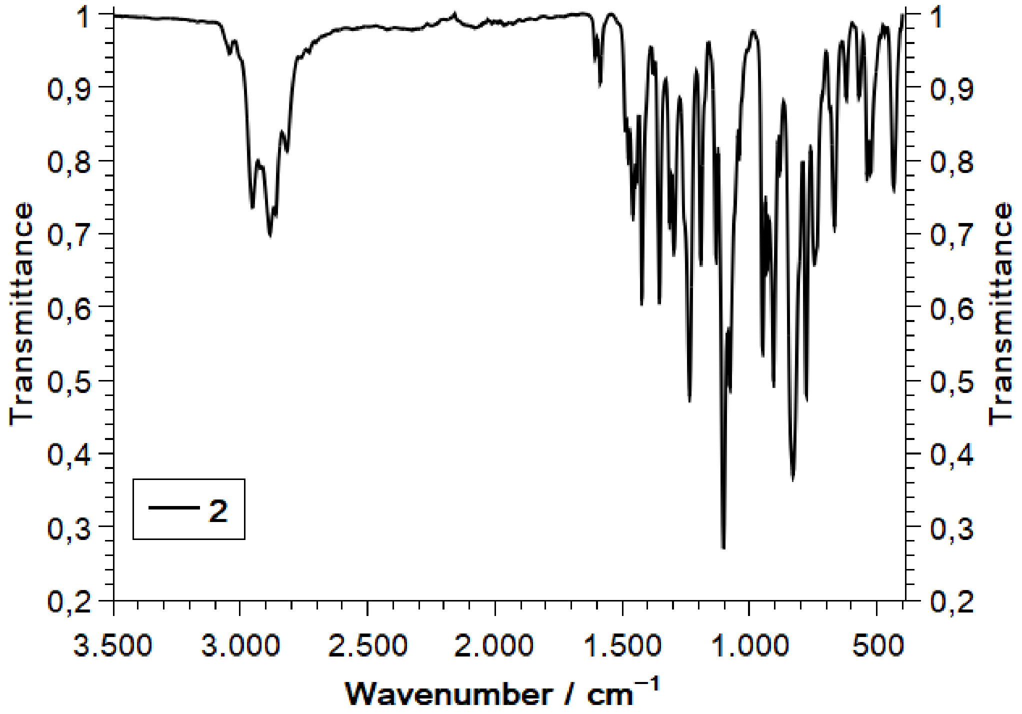

Figure S 5. ATR IR spectrum of solid K{crypt.222}[Fe(NXyl)L_2], **2**.

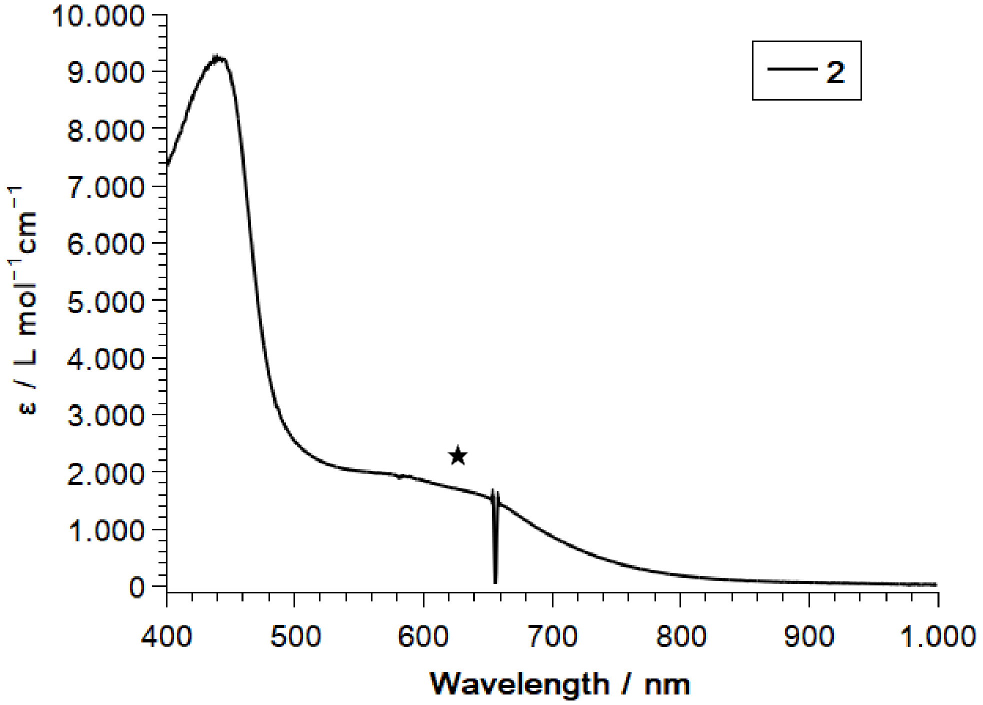

Figure S 6. UV-Vis-NIR spectrum of K{crypt.222}[Fe(NXyl)L_2], **2**, in thf. The ★ symbol corresponds to an artifact caused by the UV-Vis device.

Compound K{crypt.222}[Fe(NDipp)L$_2$], 3

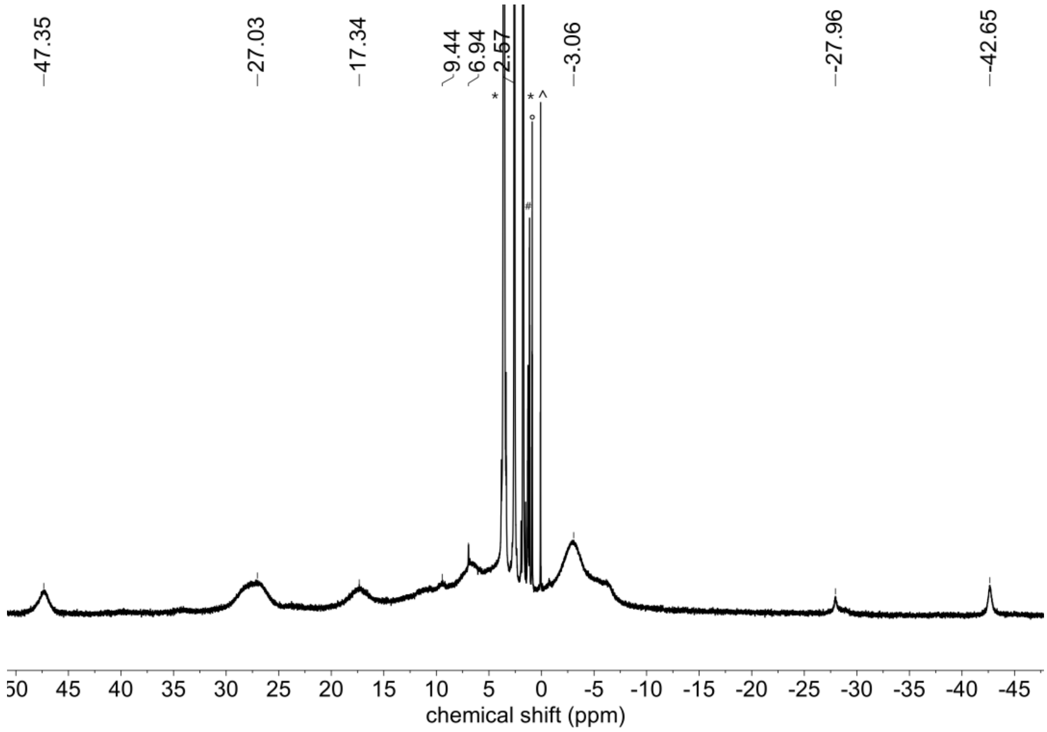

Figure S 7. ^{1}H-NMR spectrum (300 Mhz, 300 K, thf-d$_8$) of K{crypt.222}[Fe(NDipp)L$_2$], **3** (* thf-d$_8$, ° *n*-pentane, # diethyl ether, ^ decomposition).

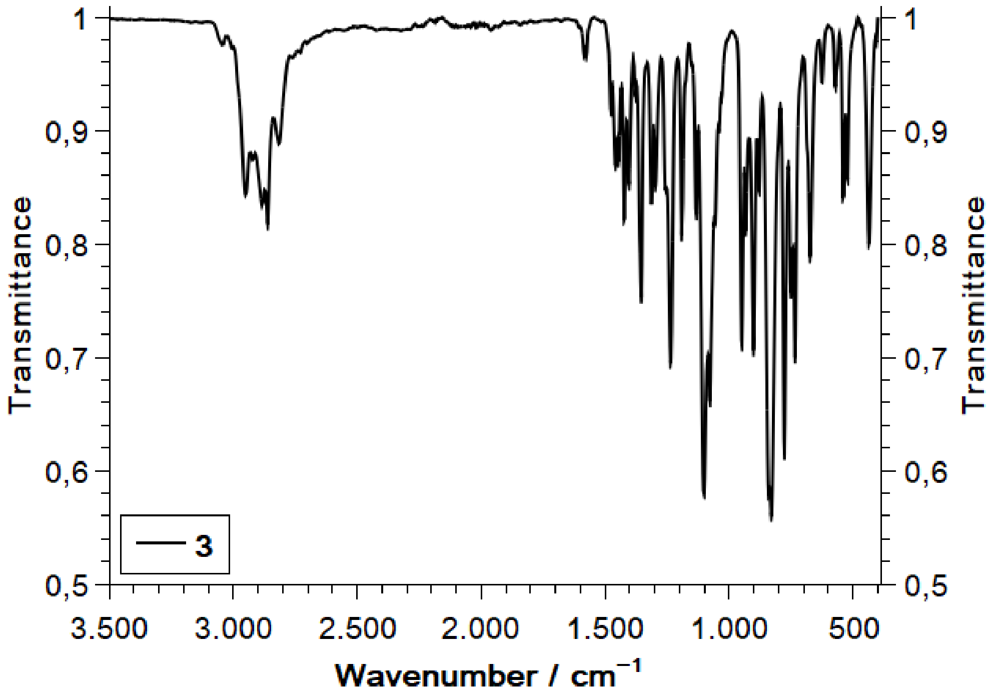

Figure S 8. ATR IR spectrum of solid K{crypt.222}[Fe(NDipp)L$_2$], **3**.

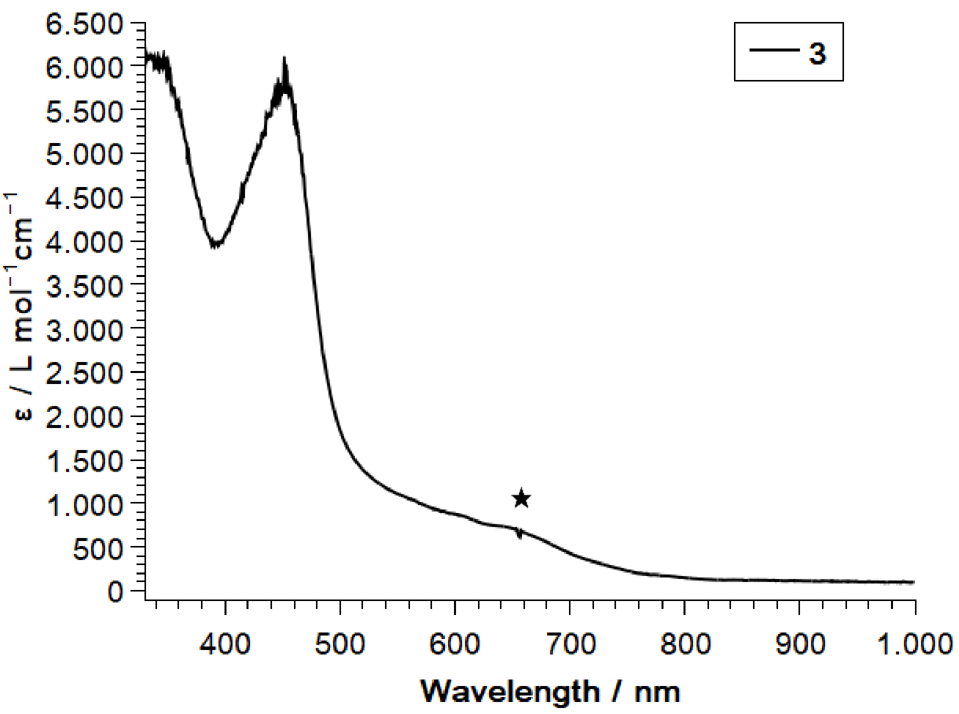

Figure S 9. UV-Vis-NIR spectrum of K{crypt.222}[Fe(NDipp)L_2], **3**, in thf. The ★ symbol corresponds to an artifact caused by the UV-Vis device.

Compound **K{crypt.222}[Fe(NDipp)L_2], 4**

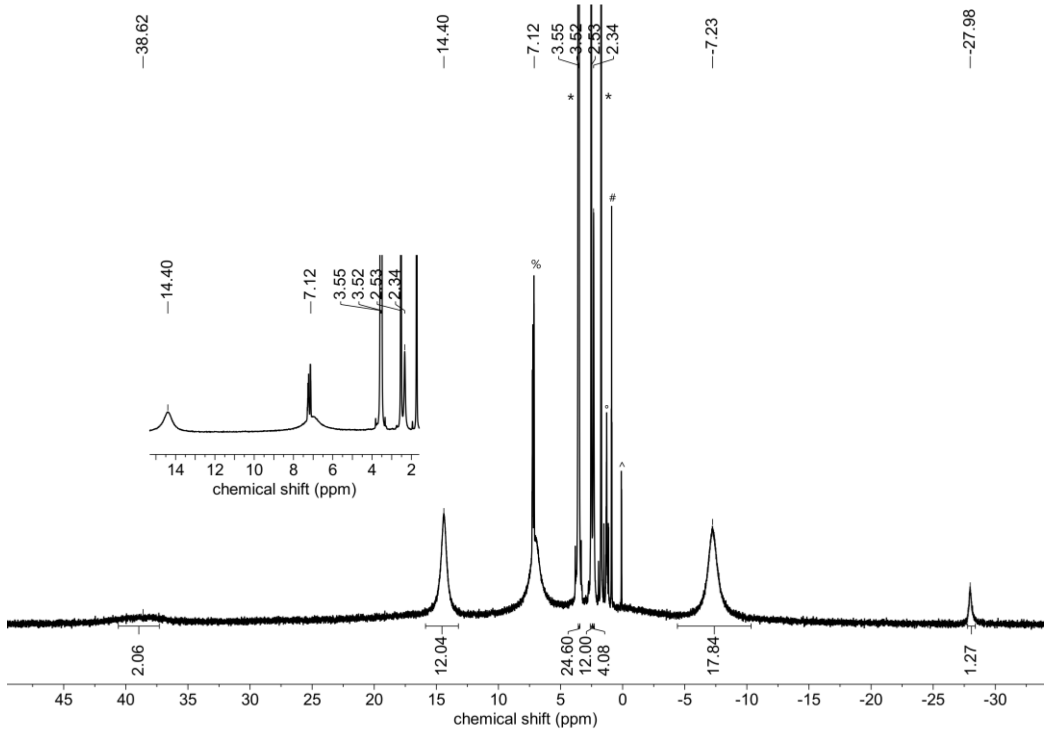

Figure S 10. ^{1}H-NMR spectrum (300 Mhz, 300 K, thf-d_8) of K{crypt.222}[Fe(NTripp)L_2], **4** (* thf-d_8, ° *n*-pentane, # diethyl ether, % 1,2-difluorobenzene, ^ decomposition).

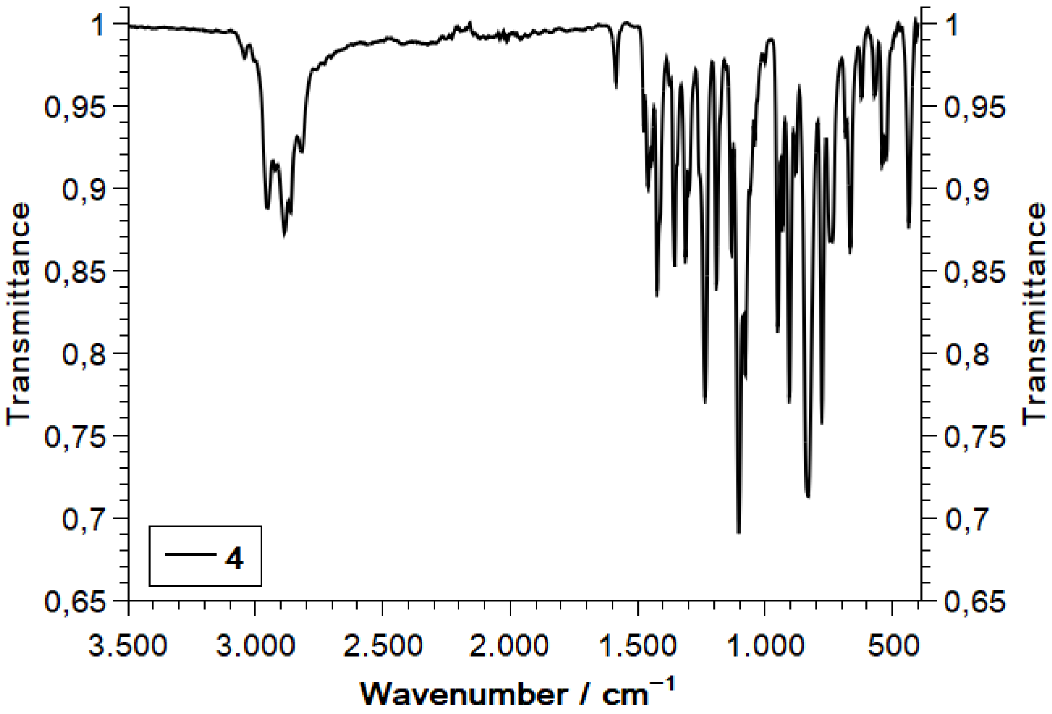

Figure S 11. ATR IR spectrum of solid K{crypt.222}[Fe(NTripp)L_2], **4**.

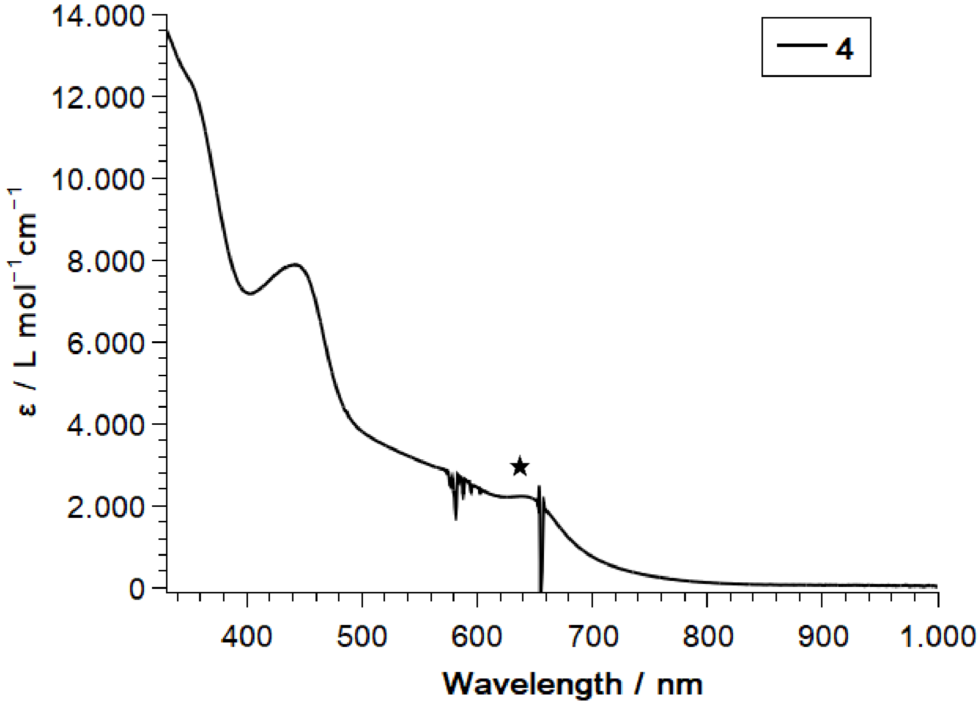

Figure S 12. UV-Vis-NIR spectrum of K{crypt.222}[Fe(NTripp)L_2], **4**, in thf. The ★ symbol corresponds to an artifact caused by the UV-Vis device.

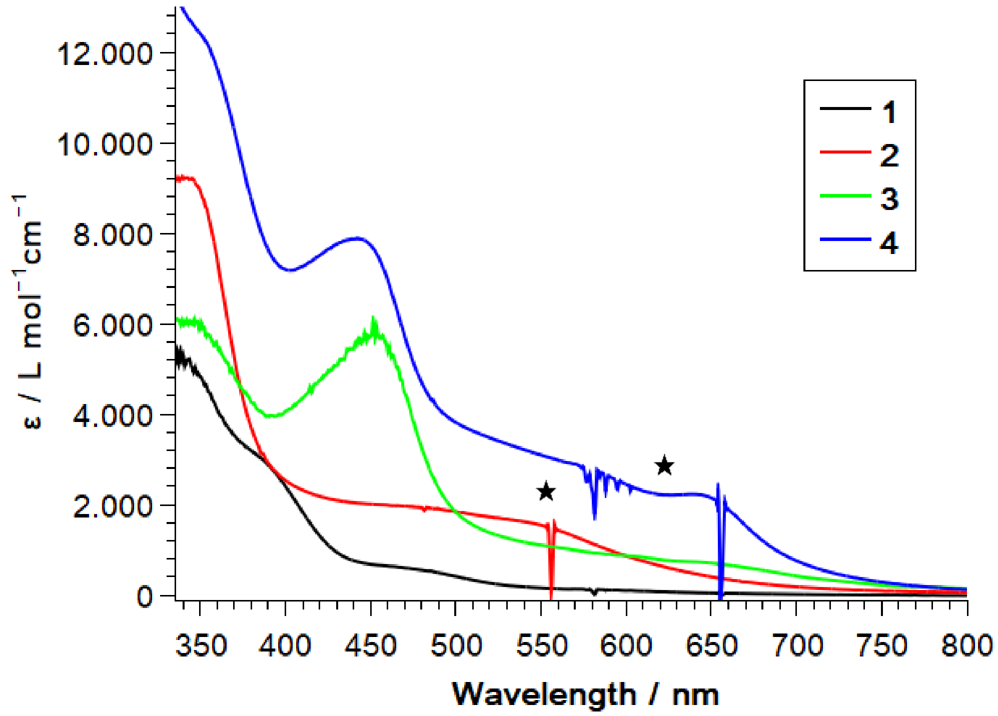

Figure S 13. Overlay of the UV-Vis-NIR spectra of complexes **1 – 4**, measured in thf. The ★ symbol corresponds to an artifact caused by the UV-Vis device.

Compound K{crypt.222}[Fe(NDipp)(N{Tripp}SiMe₃)L], 5

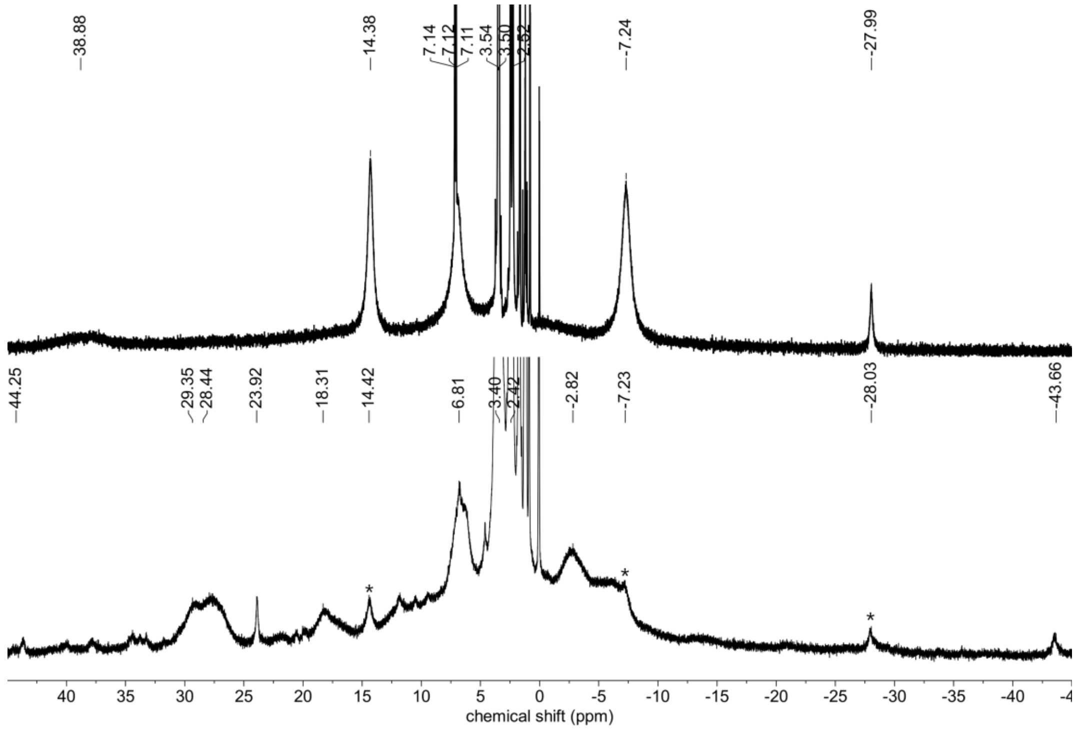

Figure S 14. ^{1}H-NMR spectrum (300 Mhz, 300 K, thf-d_8) Above: Compound **4**. Below: K{crypt.222}[Fe(NDipp)(N{Tripp}$SiMe_3$)L], **5**, marked (*) signals are assigned to compound **4**.

Compound K{18c6}[Fe(η²-κN¹:κN⁴-N_4Ph_2)L_2], 6

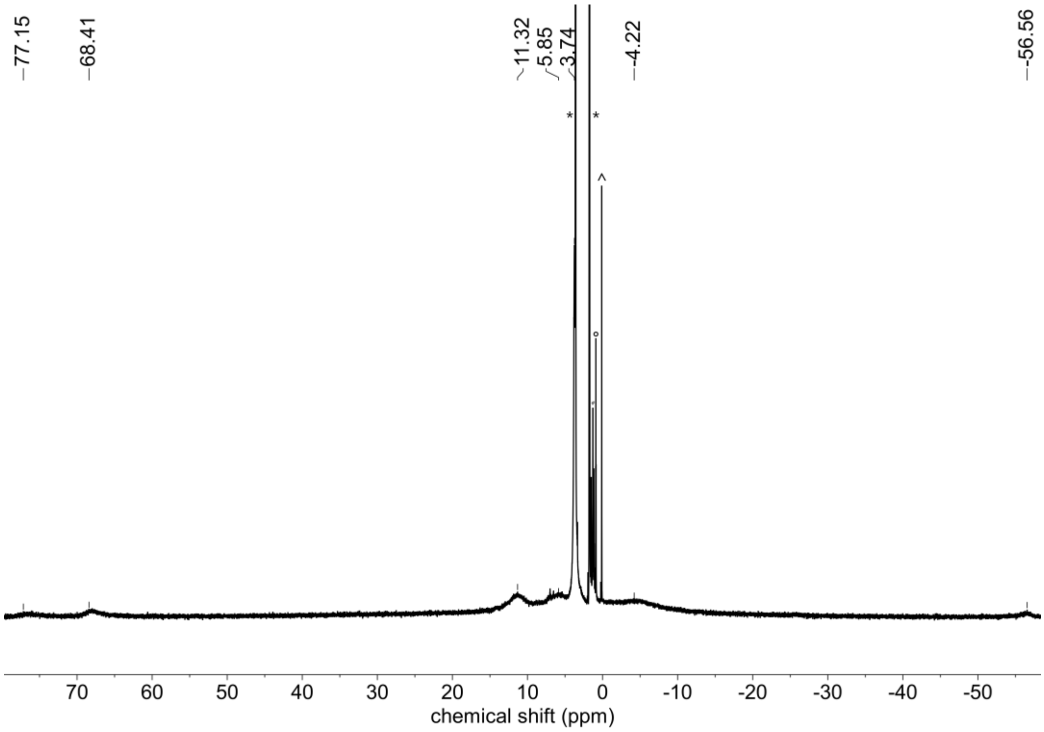

Figure S 15. ^{1}H-NMR spectrum (300 Mhz, 300 K, thf-d_8) of K{18c6}[Fe(η²-κN¹:κN⁴-N_4Ph_2)L_2], **6** (* thf-d_8, ° *n*-pentane, # diethyl ether, ^ decomposition).

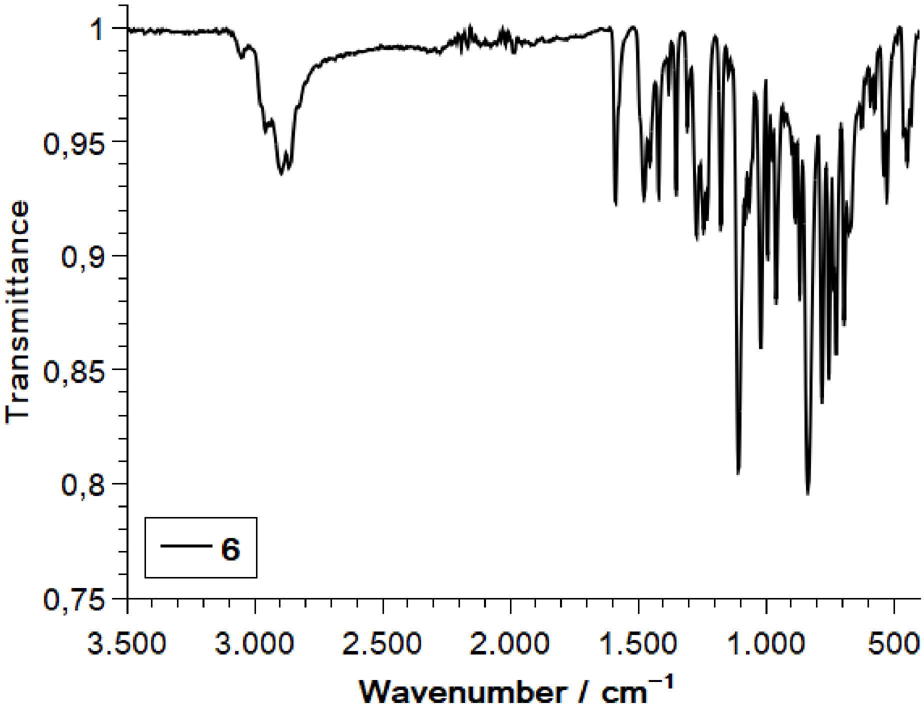

Figure S 16. ATR IR spectrum of solid K{18c6}[Fe(η²-κN¹:κN⁴-N_4Ph_2)L_2], **6**.

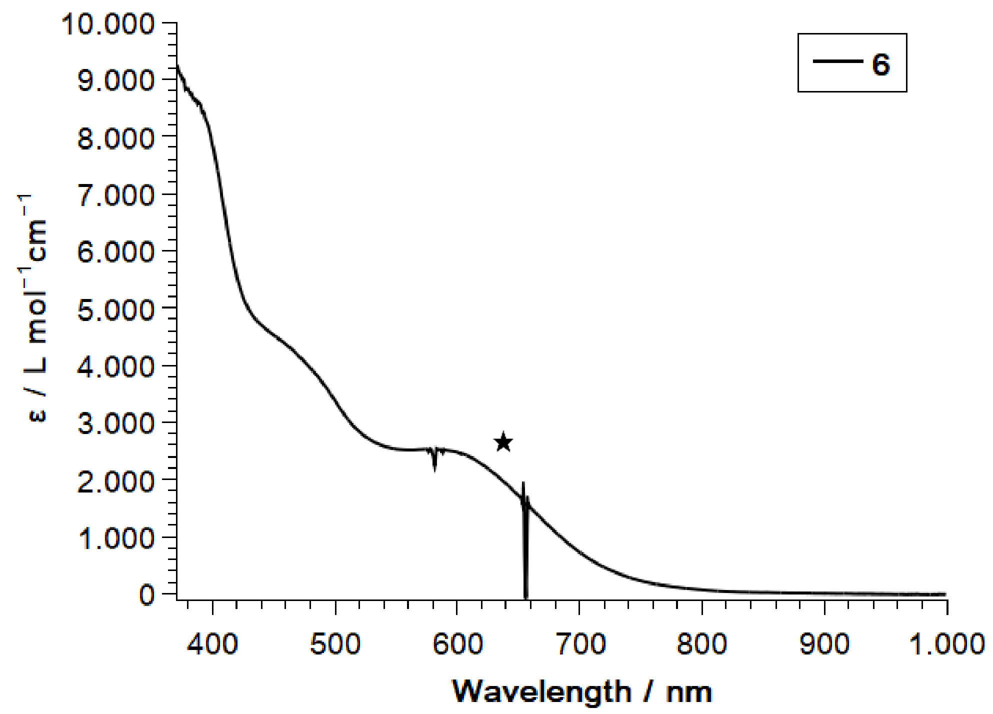

Figure S 17. UV-Vis-NIR spectrum of K{18c6}[Fe(η^2-κN^1:κN^4-N_4Ph_2)L_2], **6**, in thf. The ★ symbol corresponds to an artifact caused by the UV-Vis device.

Compound [Fe(NTol)L_2], 7

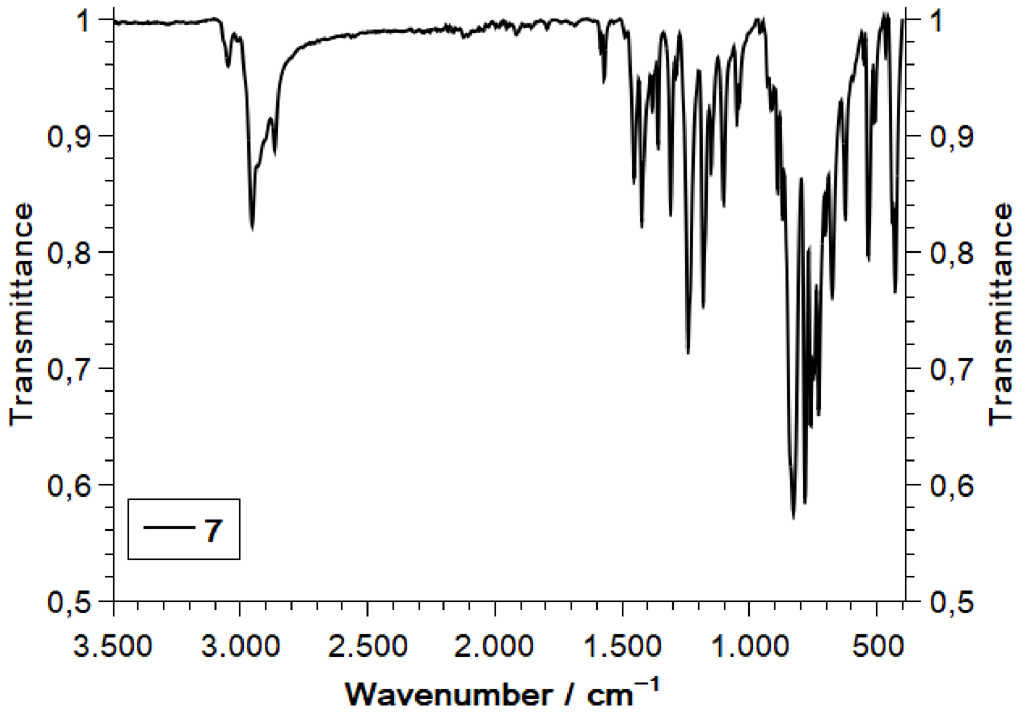

Figure S 18. ATR IR spectrum of solid [Fe(NTol)L_2], **7**.

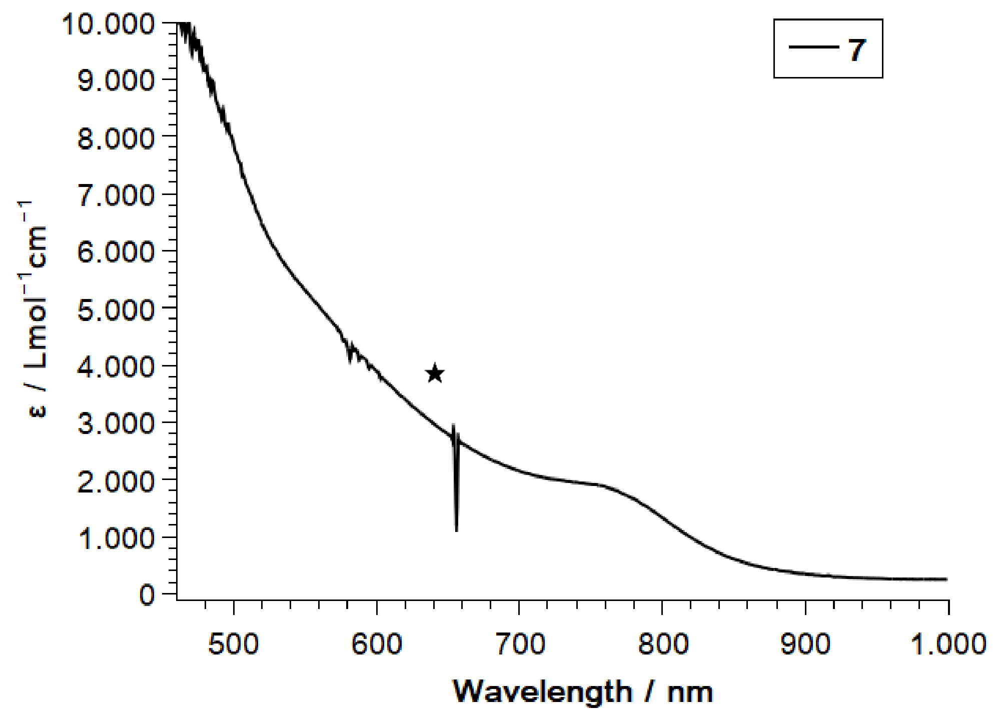

Figure S 19. UV-Vis-NIR spectrum of [Fe(NTol)L_2], **7**, in *n*-pentane. The ★ symbol corresponds to an artifact caused by the UV-Vis device.

Compound [Fe(NXyl)L_2], 8

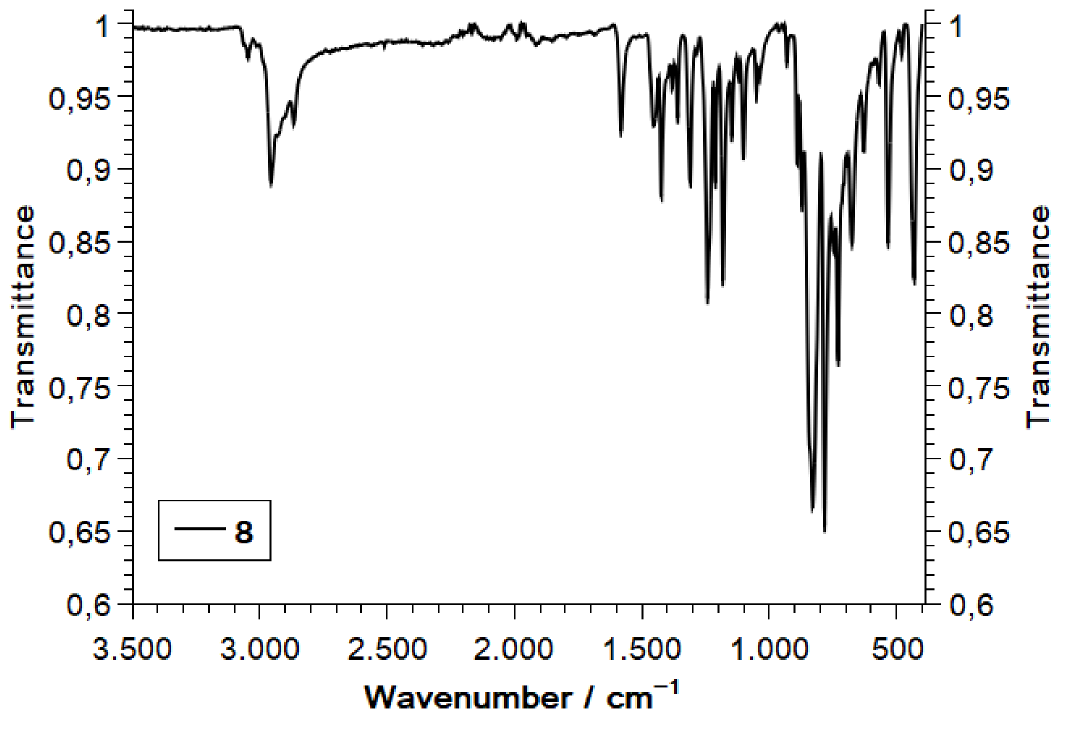

Figure S 20. ATR IR spectrum of solid [Fe(NXyl)L_2], **8**.

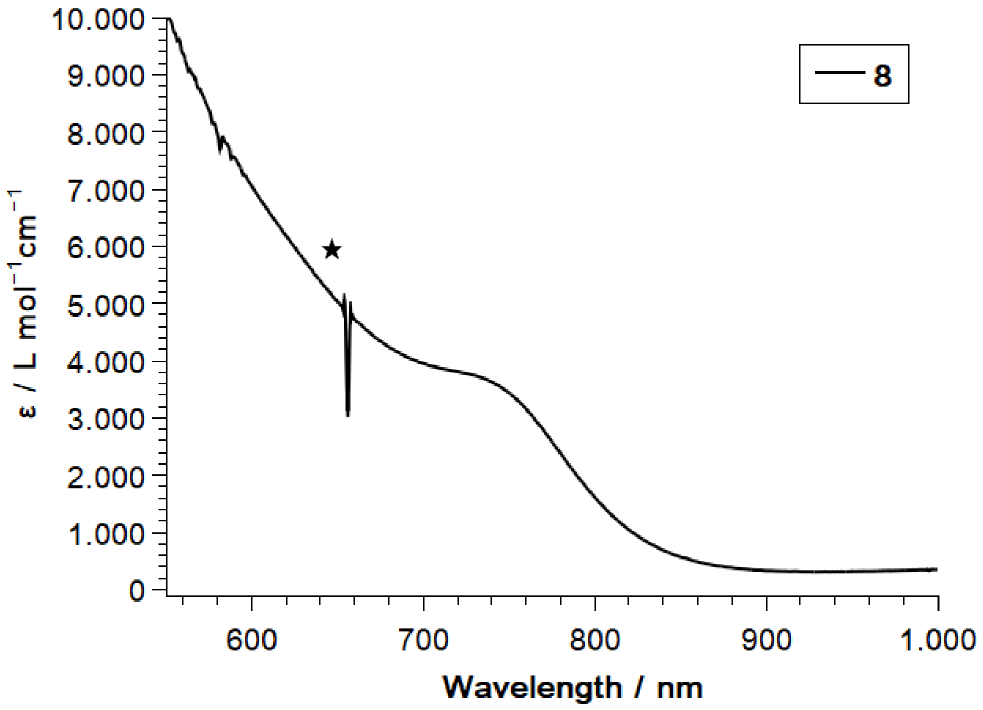

Figure S 21. UV-Vis-NIR spectrum of [Fe(NXyl)L_2], **8**, in *n*-pentane. The ★ symbol corresponds to an artifact caused by the UV-Vis device.

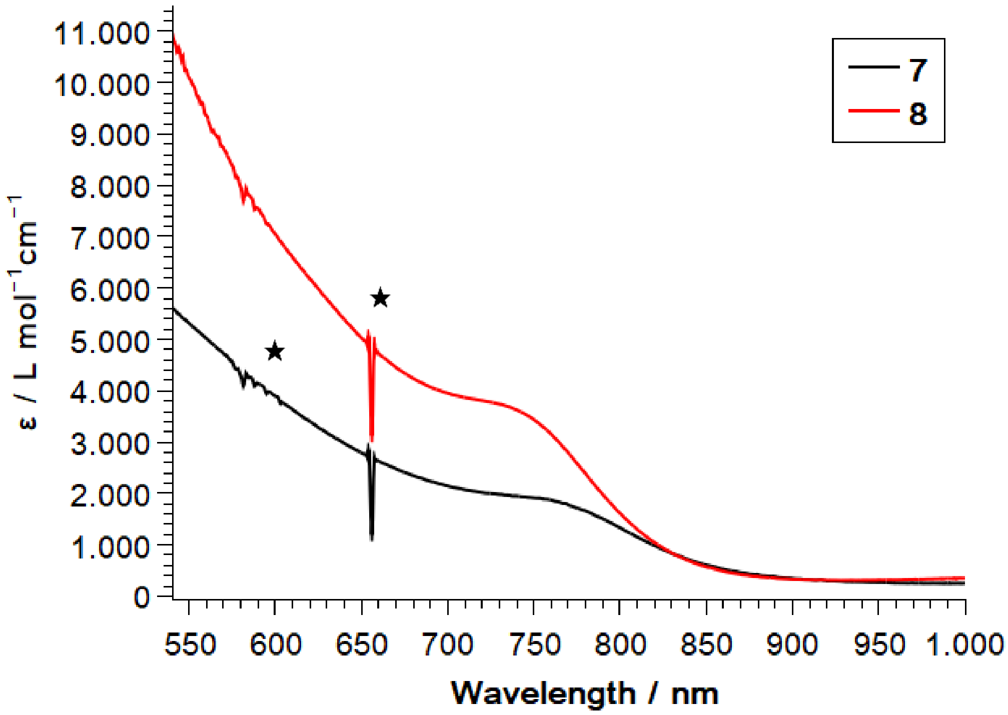

Figure S 22. Overlay of UV-Vis-NIR spectra of complexes **7** and **8**, measured in *n*-pentane. The ★ symbol corresponds to an artifact caused by the UV-Vis device.

Compound [Fe(κ^2-*N*-N(SiMe$_3$)-2-(CH$_3$)$_2$CNH{Ph}-6-^{i}Pr-phenyl)L], 9

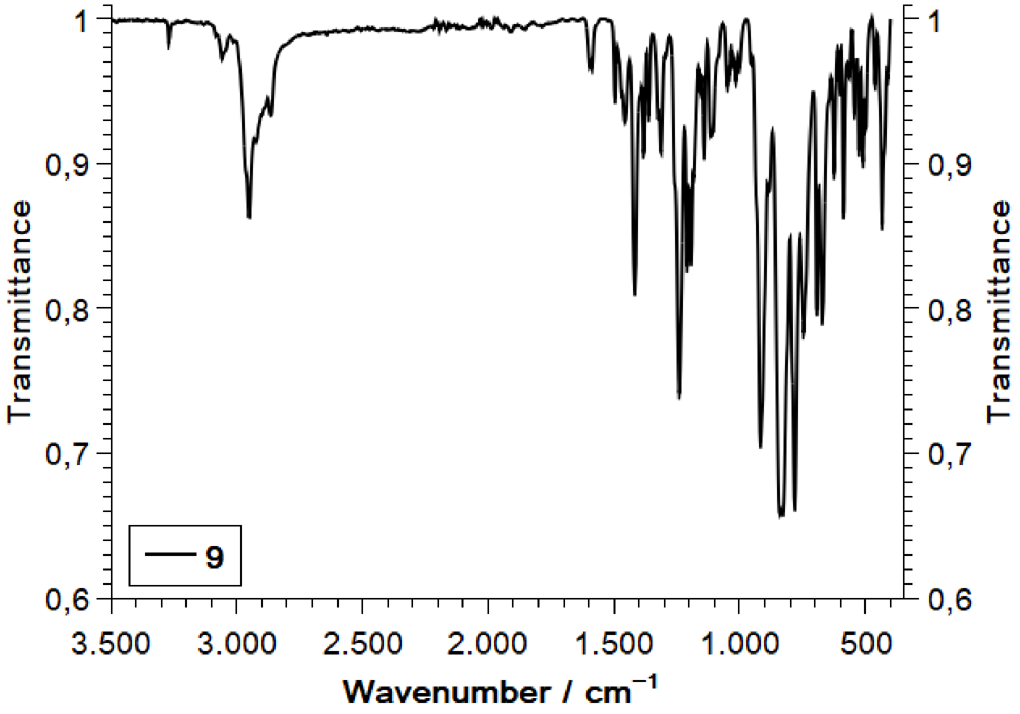

Figure S 23. ATR IR spectrum of solid [Fe(κ^2-*N*-N(SiMe$_3$)-2-(CH$_3$)$_2$CNH{Ph}-6-^{i}Pr-phenyl)L], **8.**

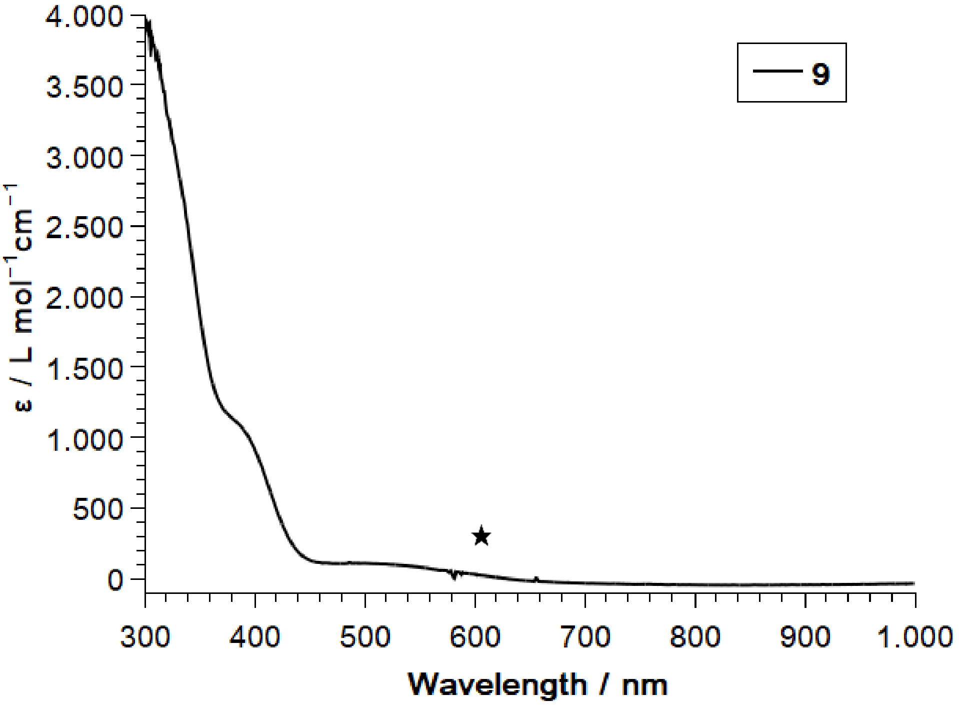

Figure S 24. UV-Vis-NIR spectrum of Fe(κ^2-*N*-N(SiMe$_3$)-2-(CH$_3$)$_2$CNH{Ph}-6-^{i}Pr-phenyl)L], **9**, in *n*-pentane. The ★ symbol corresponds to an artifact caused by the UV-Vis device.

Compound [Fe(κ^2-*N*-N(SiMe$_3$)-2-(CH$_3$)$_2$CNH{Tol}-6-^{i}Pr-phenyl)L], 10.

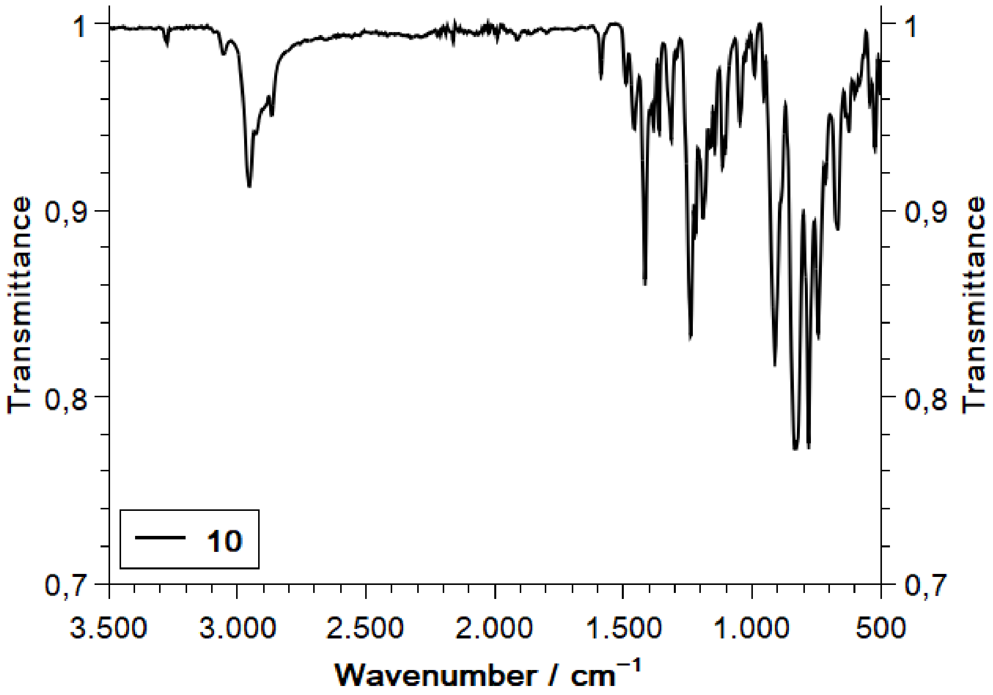

Figure S 25. ATR IR spectrum of solid [Fe(κ^2-*N*-N(SiMe$_3$)-2-(CH$_3$)$_2$CNH{Tol}-6-^{i}Pr-phenyl)L], **10**.

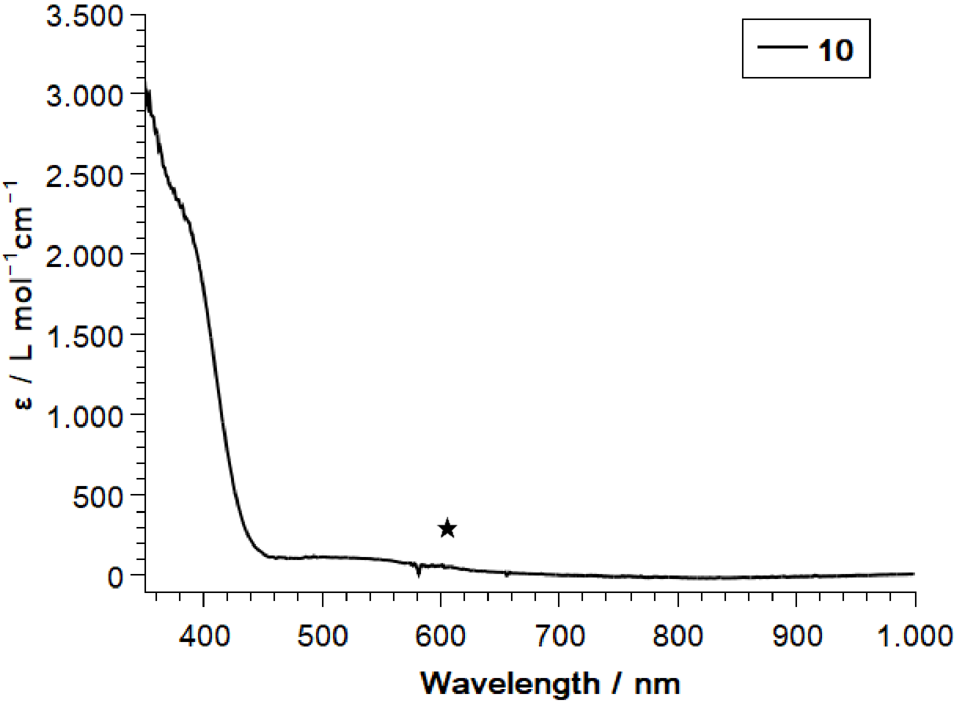

Figure S 26. UV-Vis-NIR spectrum of [Fe(κ^2-*N*-N(SiMe$_3$)-2-(CH$_3$)$_2$CNH{Tol}-6-^{i}Pr-phenyl)L], **10**, in *n*-pentane. The ★ symbol corresponds to an artifact caused by the UV-Vis device.

Compound [Fe(κ²-*N*-N(SiMe₃)-2-(CH₃)₂CNH{Tol}-6-ⁱPr-phenyl)L], 11.

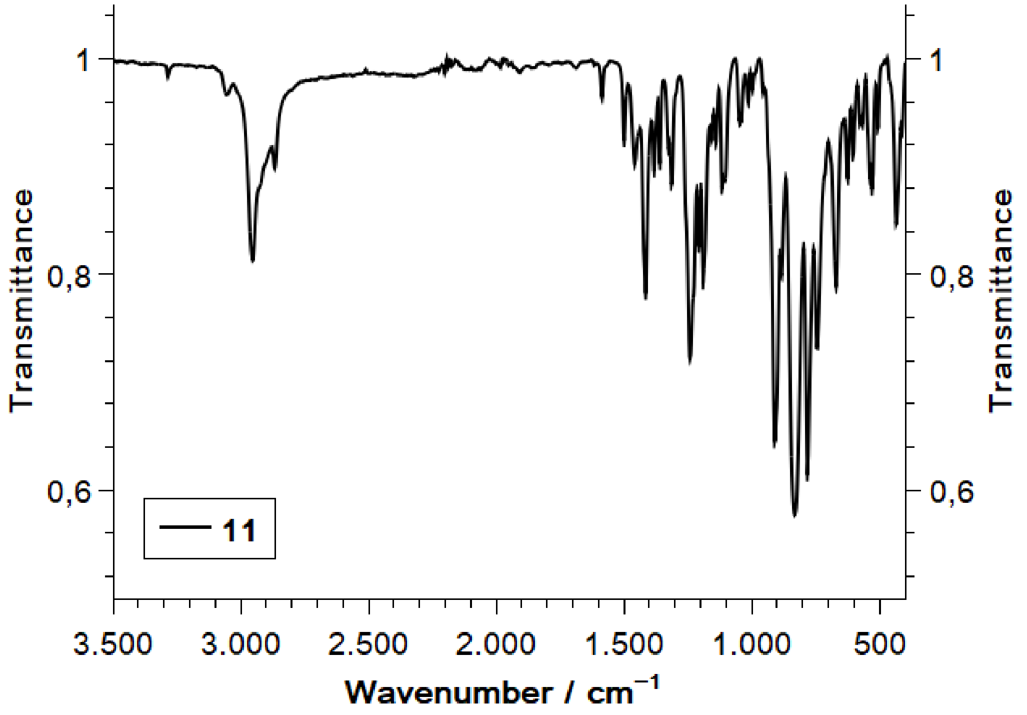

Figure S 27. ATR IR spectrum of solid [Fe(κ²-*N*-N(SiMe₃)-2-(CH₃)₂CNH{Xyl}-6-ⁱPr-phenyl)L], **11**.

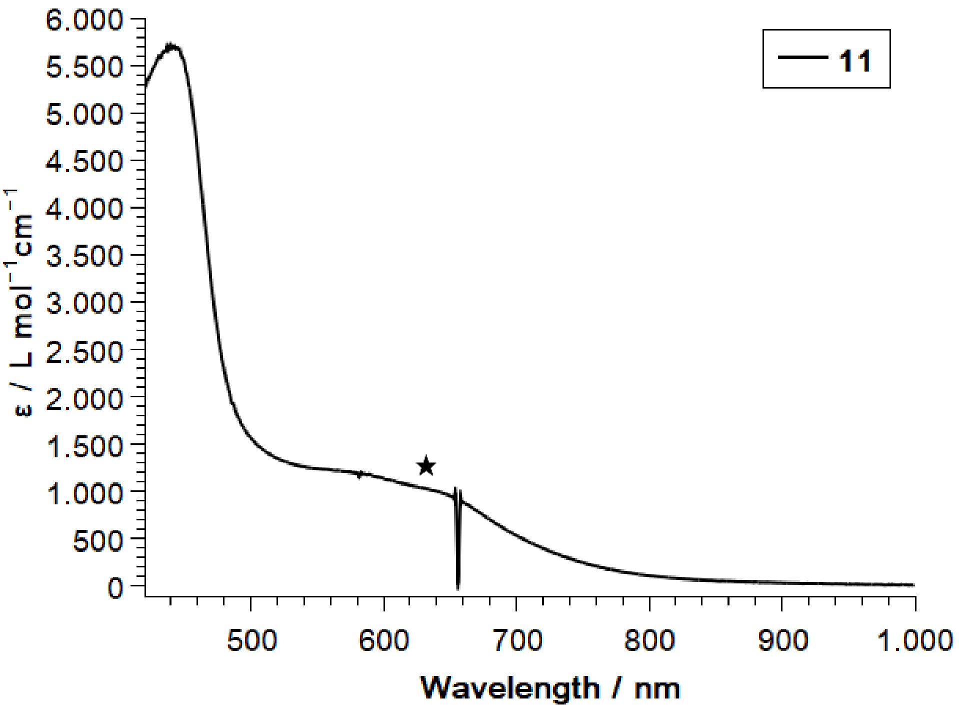

Figure S 28. UV-Vis-NIR spectrum of [Fe(κ²-*N*-N(SiMe₃)-2-(CH₃)₂CNH{Xyl}-6-ⁱPr-phenyl)L], **11**, in *n*-pentane. The ★ symbol corresponds to an artifact caused by the UV-Vis device.

Compound K{crypt.222}[FeN{Tol}C{H}{Ph}O)L$_2$], 12

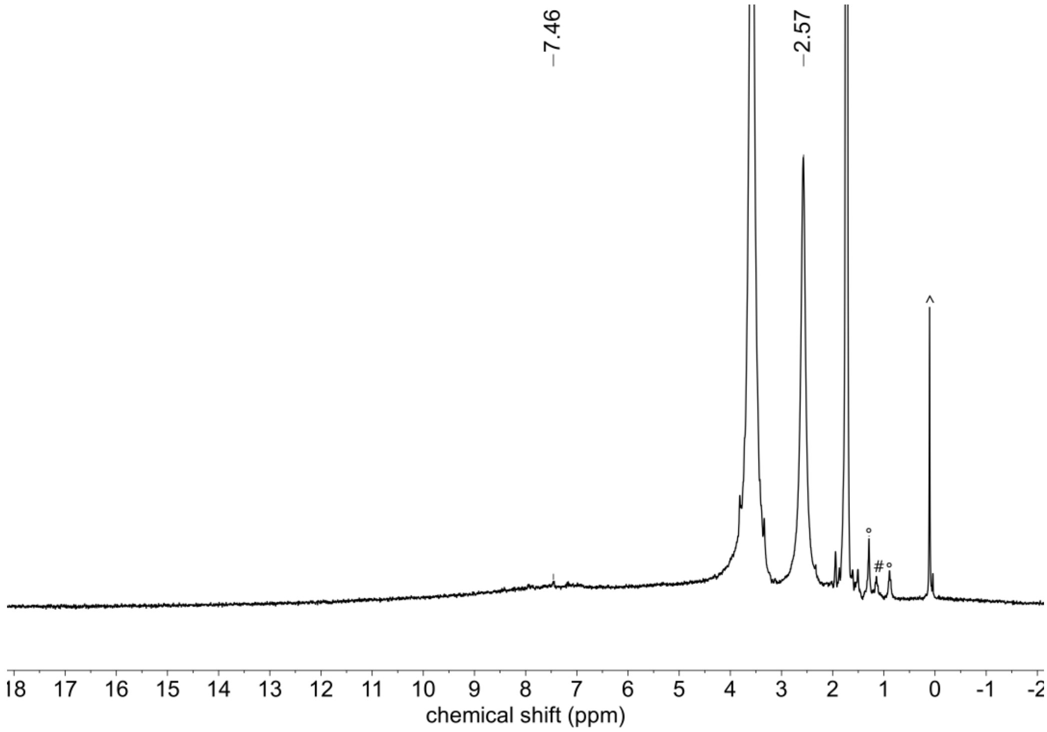

Figure S 29. ^{1}H-NMR spectrum (300 Mhz, 300 K, thf-d$_8$) of K{crypt.222}[FeN{Tol}C{H}{Ph}O)L$_2$], **12** (* thf-d$_8$, ° *n*-pentane, # diethyl ether, ^ decomposition).

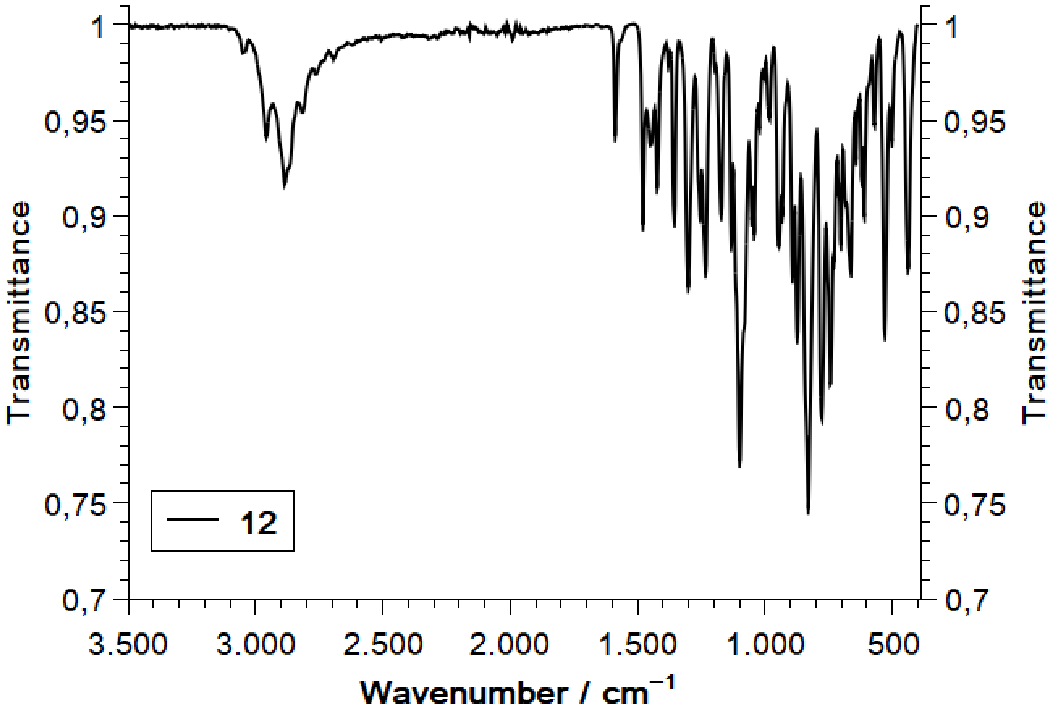

Figure S 30. ATR IR spectrum of solid K{crypt.222}[FeN{Tol}C{H}{Ph}O)L$_2$], **12**.

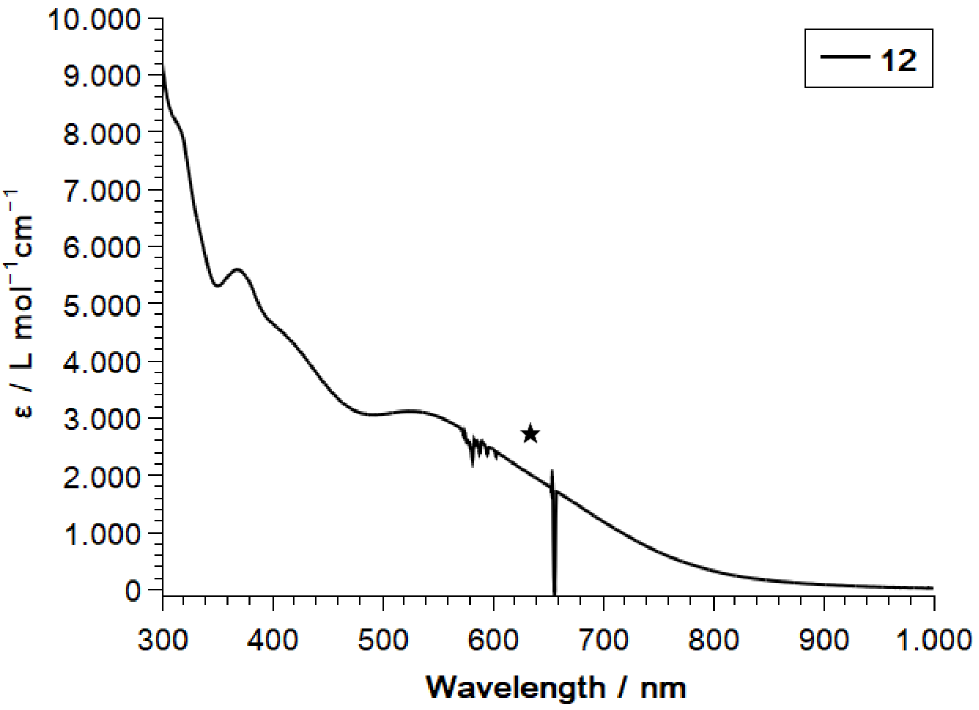

Figure S 31. UV-Vis-NIR spectrum of K{crypt.222}[FeN{Tol}C{H}{Ph}O)L$_2$], **12**, in thf. The ★ symbol corresponds to an artifact caused by the UV-Vis device.

Compound K{crypt.222}[FeN{Xyl}C{NMes}O)L$_2$], 13

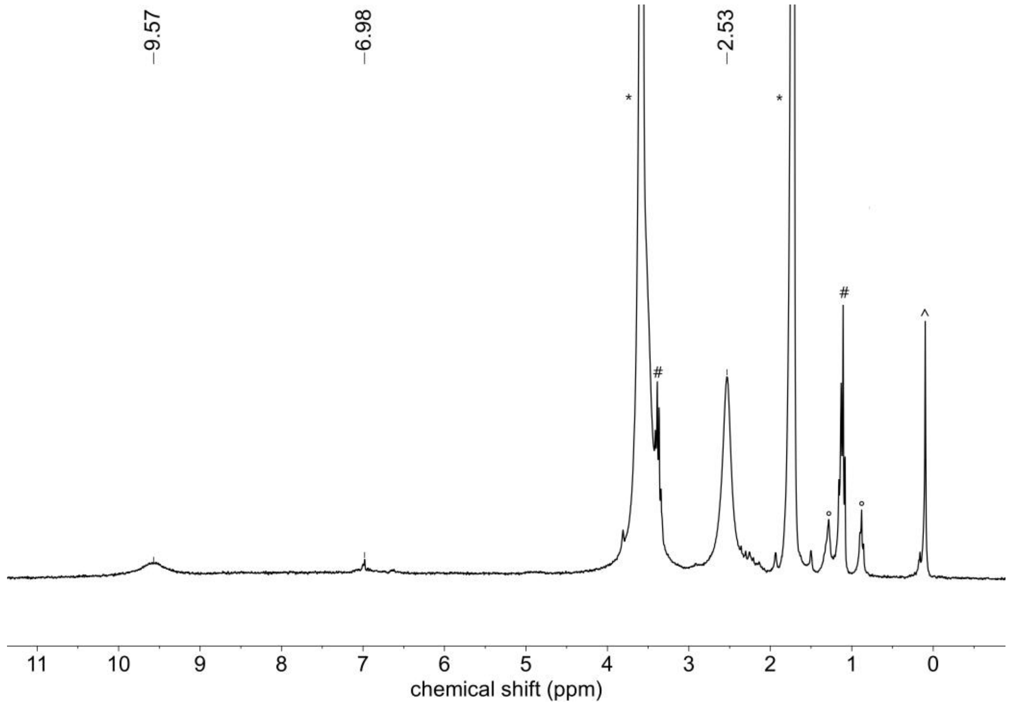

Figure S 32. ^{1}H-NMR spectrum (300 Mhz, 300 K, thf-d$_8$) of K{crypt.222}[FeN{Xyl}C{NMes}O)L$_2$], **13** (* thf-d$_8$, ° *n*-pentane, # diethyl ether, ^ decomposition).

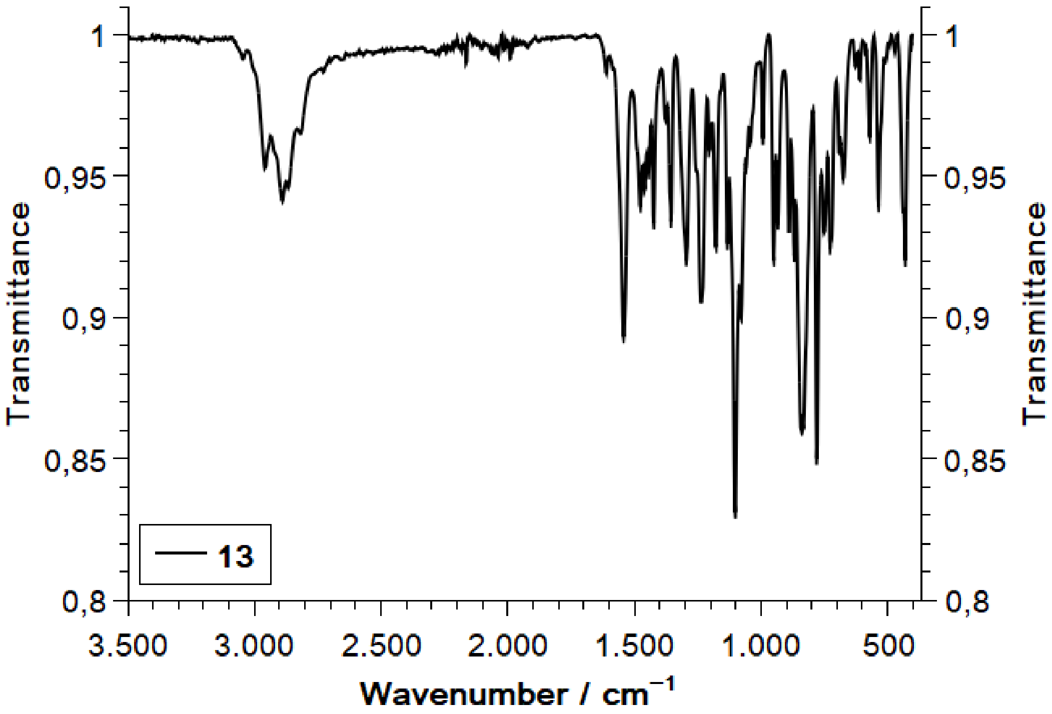

Figure S 33. ATR IR spectrum of solid K{crypt.222}[FeN{Xyl}C{NMes}O)L_2], **13**.

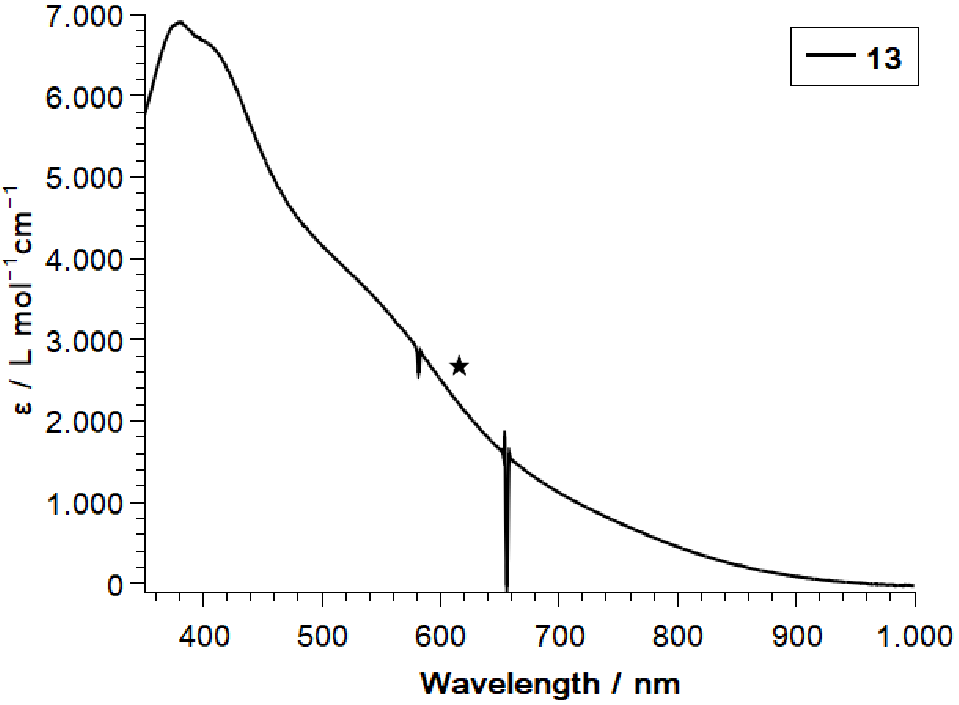

Figure S 34. UV-Vis-NIR spectrum of K{crypt.222}[FeN{Xyl}C{NMes}O)L_2], **13**, in thf. The ★ symbol corresponds to an artifact caused by the UV-Vis device.

2. Computational Details

Computational analysis of [Fe(NPh)L$_2$]$^-$

Table S1. Single point energies, geometries and Loewdin spin densities/atomic charges of **[Fe(NPh)L$_2$]$^-$**.

Spin state	$\Delta E_{sext\text{->}quar}$ (kJ/mol)	Fe–N (Å)	Fe–N–C (°)	Fe–N_{amide1} (Å)	Fe–N_{amido2} (Å)	N–C (Å)	Loewdin spin density (charges) Fe	N
**PBE**								
quartet	-2.5	1.71	175	1.89	1.91	1.34	2.56 (-0.23)	-0.01 (0.08)
sextet	0	1.77	169	1.92	1.94	1.33	3.62 (-0.08)	0.51 (0.05)
**PBE0**								
quartet	+27.7	1.79	179	1.93	1.93	1.33	3.35 (-0.05)	-0.49 (0.09)
sextet	0	1.76	171	1.94	1.93	1.33	3.87 (+0.03)	0.53 (0.02)
**TPSSh**								
quartet	+12.4	1.72	175	1.9	1.91	1.34	2.85 (-0.16)	-0.16 (0.06)
sextet	0	1.77	172	1.92	1.94	1.33	3.75 (-0.03)	0.54 (0.03)
**Comparison with experimental values of [1]$^-$ ([Fe(NTol)L$_2$]$^-$)**								
		1.765(2)	175.5(2)	1.966(3)	1.947(3)	1.3553(13)		

```
LOWEST ROOT (ROOT 0, MULT 6) =  -3426.526343171 Eh -93240.522 eV
STATE ROOT MULT  DE/a.u.     DE/eV    DE/cm**-1
   1:   1    4   0.009798     0.267   2150.5
   2:   0    4   0.013084     0.356   2871.6
   3:   3    4   0.026342     0.717   5781.3
   4:   2    4   0.028161     0.766   6180.7
   5:   4    4   0.039568     1.077   8684.3
   6:   1    6   0.043783     1.191   9609.4
   7:   2    6   0.045356     1.234   9954.6
   8:   0    2   0.060936     1.658  13373.9
```

Figure S 35. Vertical excitation energies of **[Fe(NPh)L$_2$]$^-$** obtained from CASSCF(13,10)/NEVPT2 calculations.

```
---------------------------------------------
CAS-SCF STATES FOR BLOCK  1 MULT= 6 NROOTS= 1
---------------------------------------------
ROOT   0:  E=   -3417.8910923823 Eh
      0.89531 [  1379]: 2222111110
      0.07516 [  1305]: 2211221110
      0.01583 [   890]: 2022111112
      0.00857 [   804]: 1221212110
```

Figure S 36. Weighted configurations of the sextet ground state of **[Fe(NPh)L$_2$]$^-$** from CASSCF(13,10) calculations.

	168	169	170	171	172	173
	-0.47337	-0.23116	-0.25196	-0.32300	-0.04150	-0.00127
	1.98854	1.96446	1.92044	1.91259	1.08736	1.07943
	--------	--------	--------	--------	--------	--------
0 Fe px	7.5	0.0	0.0	0.0	0.0	0.1
0 Fe dxz	0.0	0.0	0.0	72.7	30.1	0.0
0 Fe dx2y2	11.7	0.0	0.3	0.0	0.0	1.5
0 Fe dxy	0.3	0.0	18.4	0.0	0.0	78.9
2 N s	10.5	0.0	0.0	0.0	0.0	0.0
2 N pz	0.0	0.0	0.1	20.8	53.9	0.0
2 N px	42.5	0.0	1.1	0.0	0.1	0.2
2 N py	1.0	0.0	54.3	0.0	0.1	8.3
6 C px	7.9	0.0	0.0	0.0	0.0	0.0
6 C dxz	0.0	0.1	0.0	2.0	6.6	0.0
6 C dxy	0.0	0.0	6.6	0.0	0.0	1.4
14 C py	0.0	20.3	3.4	0.0	0.0	0.7
15 C py	0.0	20.2	3.3	0.0	0.0	0.7
24 C py	0.0	19.6	0.0	0.0	0.0	0.0
25 C py	0.0	19.6	0.0	0.0	0.0	0.0

	174	175	176	177	178	179
	0.03786	-0.00912	-0.00770	0.27440	0.21163	0.22045
	1.01142	1.00007	1.00006	0.03563	0.00000	0.00000
	--------	--------	--------	--------	--------	--------
0 Fe dz2	5.8	1.0	87.8	0.0	0.1	0.1
0 Fe dyz	1.0	94.4	1.7	0.0	0.0	0.1
0 Fe dx2y2	79.0	1.9	5.3	0.0	0.1	0.0
14 C py	0.0	0.0	0.0	17.3	0.0	0.0
15 C py	0.0	0.0	0.0	17.3	0.0	0.0
22 C py	0.0	0.0	0.0	0.0	0.2	5.1
24 C py	0.0	0.0	0.0	17.5	0.0	0.0
25 C py	0.0	0.0	0.0	17.5	0.0	0.0
26 C py	0.0	0.0	0.0	0.0	0.1	5.3
34 C dyz	0.0	0.0	0.0	5.1	0.0	0.0

Figure S 37. Loewdin reduced active orbitals of the sextet state of **Fe(NPh)L$_2$]$^-$** from CASSCF(13,10) calculations.

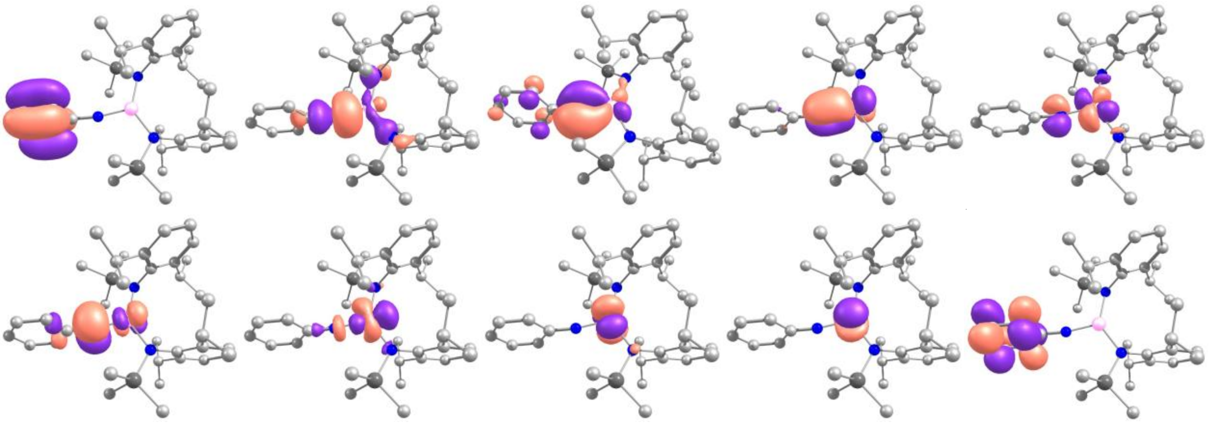

Figure S 38. Depiction of orbitals 168-177 of the sextet ground state of **Fe(NPh)L$_2$]$^-$** from CASSCF(13,10) calculations.

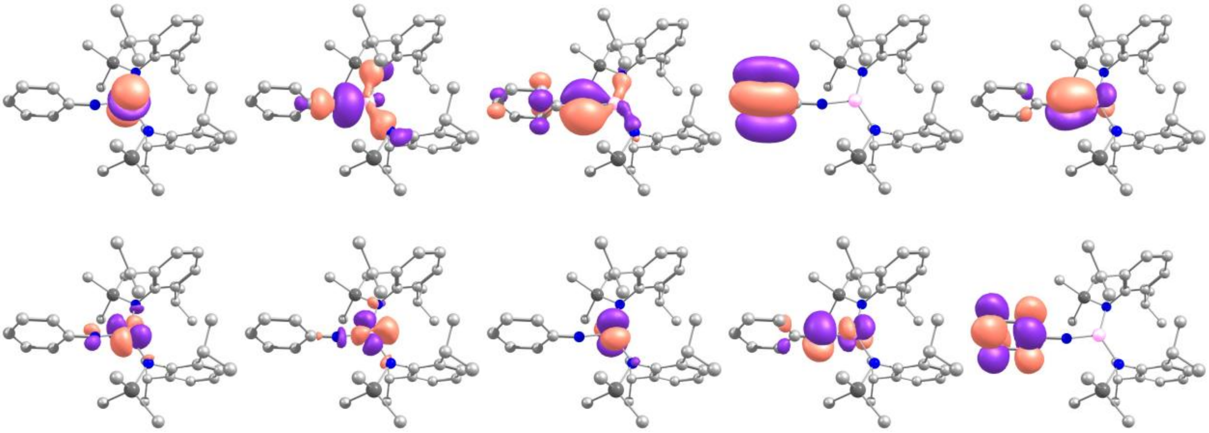

Figure S 39. Depiction of orbitals 168-177 of the quartet ground state of **$Fe(NPh)L_2]^-$** from CASSCF(13,10) calculations.

Computational analysis of [Fe(NPh)L_2]

Table S2. Single point energies, geometries and Loewdin spin densities/atomic charges of **[Fe(NPh)L_2]**.

Spin state	$\Delta E_{q\text{->}t}$ (kJ/mol)	Fe–N (Å)	Fe–N–C (°)	Fe–N_{amide1} (Å)	Fe–N_{amido2} (Å)	N–C (Å)	Loewdin spin density (charges)	
							Fe	N
**PBE**								
triplet	-21.0	1.65	143	1.83	1.81	1.37	1.81 (-0.18)	-0.01 (0.14)
quintet	0	1.72	178	1.85	1.87	1.35	3.13 (-0.09)	0.27 (0.11)
**PBE0**								
triplet	58.2	1.74	175	1.86	1.84	1.33	2.89 (-0.04)	-0.64 (0.16)
quintet	0	1.76	172	1.88	1.87	1.32	3.84 (-0.09)	-0.10 (0.10)
**TPSSh**								
triplet	+4.9	1.68	133	1.83	1.82	1.37	2.24 (-0.12)	-0.24 (0.12)
quintet	0	1.73	174	1.86	1.88	1.34	3.47 (-0.01)	0.10 (0.10)
**Comparison with experimental values of [1] ([Fe(NTol)L_2])**								
		1.747(2)	170(4)	1.899(2)	1.894(2)	1.337(3)		

```
LOWEST ROOT (ROOT 0, MULT 5) =  -3426.449968096 Eh -93238.444 eV
STATE ROOT MULT  DE/a.u.     DE/eV    DE/cm**-1
   1:   0    3   0.030787     0.838    6756.9
   2:   4    3   0.041195     1.121    9041.4
   3:   1    5   0.041264     1.123    9056.4
   4:   3    3   0.043834     1.193    9620.4
   5:   1    3   0.046870     1.275   10286.7
   6:   2    3   0.055329     1.506   12143.4
   7:   0    1   0.059289     1.613   13012.3
```

Figure S 40. Vertical excitation energies of **[Fe(NPh)L_2]** obtained from CASSCF(12,10)/NEVPT2 calculations.

```
CAS-SCF STATES FOR BLOCK  1 MULT= 5 NROOTS= 1
---------------------------------------------
ROOT   0:  E=   -3417.8161917262 Eh
      0.66090 [  4034]: 2222111100
      0.17935 [  3999]: 2221111110
      0.11301 [  3939]: 2220111120
      0.01547 [  3481]: 2121211110
      0.01240 [  3653]: 2202111102
      0.00379 [  2408]: 1221121110
      0.00338 [  3593]: 2201111112
      0.00257 [  3796]: 2211111111
```

Figure S 41. Weighted configurations of the quintet ground state of **[Fe(NPh)L$_2$]** from CASSCF(12,10) calculations.

	168	169	170	171	172	173
	-0.63644	-0.45466	-0.35014	-0.36250	-0.26167	-0.22016
	1.99453	1.97853	1.96016	1.55861	1.02044	1.00524
0 Fe px	7.3	0.0	0.0	0.0	0.0	3.0
0 Fe dxz	0.0	16.3	0.0	0.7	86.8	0.1
0 Fe dx2y2	16.1	0.0	0.0	0.1	0.1	82.4
0 Fe dxy	0.3	0.3	0.0	49.8	0.9	0.1
1 N px	8.4	0.0	0.0	0.1	0.0	1.8
2 N s	10.0	0.0	0.0	0.0	0.0	1.1
2 N pz	0.0	64.0	0.0	0.3	7.6	0.0
2 N px	33.3	0.1	0.0	0.0	0.0	1.8
2 N py	0.0	0.7	0.0	29.6	0.0	0.0
6 C px	5.1	0.0	0.0	0.0	0.0	0.5
6 C dxz	0.0	6.7	0.0	0.0	1.1	0.0
14 C py	0.0	0.0	19.8	2.2	0.0	0.0
15 C py	0.0	0.0	20.0	2.2	0.0	0.0
24 C py	0.0	0.0	20.3	0.0	0.0	0.0
25 C py	0.0	0.0	20.4	0.0	0.0	0.0

	174	175	176	177	178	179
	-0.27109	-0.27757	-0.03287	0.14940	0.12217	0.12346
	1.00016	0.99989	0.44261	0.03984	0.00000	0.00000
0 Fe dz2	3.2	89.5	0.0	0.0	0.4	0.1
0 Fe dyz	89.5	4.0	0.0	0.0	0.0	0.0
0 Fe dxy	0.0	0.0	46.4	0.0	0.0	0.8
2 N py	0.0	0.0	32.8	0.0	0.1	1.3
6 C py	0.0	0.0	0.3	0.0	1.1	15.0
14 C py	0.0	0.0	2.5	17.8	0.3	3.7
15 C py	0.0	0.0	2.5	18.1	0.4	3.7
24 C py	0.0	0.0	0.0	17.3	0.3	4.5
25 C py	0.0	0.0	0.0	17.2	0.3	4.6
34 C py	0.0	0.0	1.7	0.0	1.2	17.7

Figure S 42. Loewdin reduced active orbitals of the quintet state of **[Fe(NPh)L$_2$]** from CASSCF(12,10) calculations.

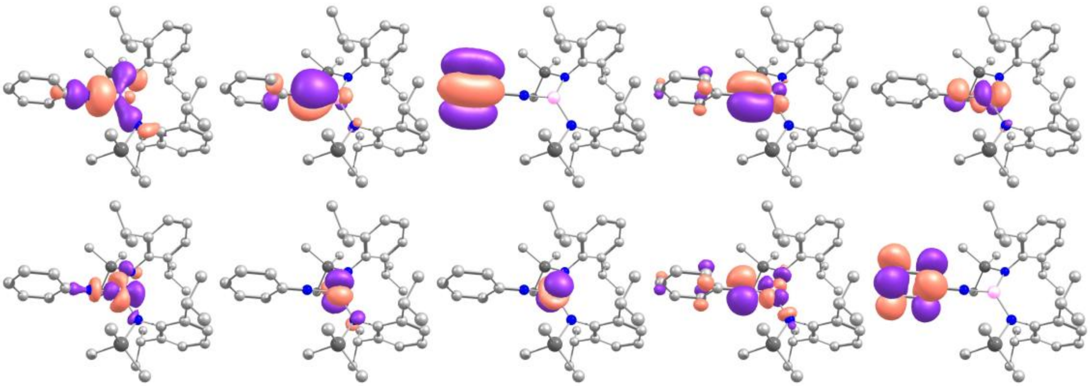

Figure S 43. Depiction of the active orbitals 168-177 of the quintet ground state of **Fe(NPh)L$_2$]** from CASSCF(12,10) calculations.

Computational analysis of $[Fe(NXyl)L_2]^-$

Table S3. Relative single point energies, geometries and Loewdin spin densities/atomic charges of $[Fe(NXyl)L_2]^-$.

Spin state	$\Delta E_{sext\text{->}quar}$ (kJ/mol)	Fe–N (Å)	Fe–N–C (°)	Fe–N_{amide1} (Å)	Fe–N_{amido2} (Å)	N–C (Å)	Loewdin spin density (charges)	
							Fe	N
**PBE**								
quartet	+3	1.71	161	1.91	1.89	1.35	2.60 (-0.25)	-0.09 (0.07)
sextet	0	1.77	179	1.93	1.94	1.35	3.62 (-0.07)	0.51 (0.06)
**PBE0**								
quartet	-17.0	1.80	161	1.95	1.94	1.33	3.42 (-0.07)	-0.31 (0.05)
sextet	0	1.76	178	1.95	1.95	1.33	3.87 (0.04)	0.53 (0.03)
**TPSSh**								
quartet	+9.0	1.73	163	1.91	1.91	1.34	2.97 (-0.15)	-0.06 (0.03)
sextet	0	1.77	177	1.86	1.94	1.33	3.62 (-0.07)	0.51 (0.06)
**Comparison with experimental values of [2] ($[Fe(NXyl)L_2]^-$)**								
		1.767(8)	174.8(7)	1.947(8)	1.947(8)	1.338(7)		

Computational analysis of $[Fe(NXyl)L_2]$

Table S4. Relative single point energies, geometries and Loewdin spin densities/atomic charges of $[Fe(NXyl)L_2]$.

Spin state	$\Delta E_{q\text{->}t}$ (kJ/mol)	Fe–N (Å)	Fe–N–C (°)	Fe–N_{amide1} (Å)	Fe–N_{amido2} (Å)	N–C (Å)	Loewdin spin density (charges)	
							Fe	N
**PBE**								
triplet	-14.5	1.66	148	1.83	1.82	1.36	1.79 (-0.20)	-0.02 (0.15)
quintet	0	1.72	173	1.87	1.87	1.34	3.19 (-0.07)	0.24 (0.12)
**PBE0**								
triplet	+37.3	1.73	140	1.85	1.85	1.33	2.75 (-0.04)	-0.44 (-0.05)
quintet	0	1.76	173	1.88	1.88	1.32	3.84 (0.08)	-0.06 (0.12)
**TPSSh**								
triplet	+5.6	1.68	142	1,83	1,84	1,35	2.28 (-0.13)	-0.21 (0.13)
quintet	0	1.73	173	1.87	1.87	1.33	3.52 (0.03)	0.09 (0.11)
**Comparison with experimental values of [7] ($[Fe(NXyl)L_2]$)**								
		1.749(2)	176.1(2)	1.896(2)	1.904(2)	1.332(2)		

Computational analysis of $[Fe(NTripp)L_2]^-$

Table S5. Single point energies, geometries and Loewdin spin densities/atomic charges of **$[Fe(NTripp)L_2]^-$**.

Spin state	$\Delta E_{sext\text{->}quar}$ (kJ/mol)	Fe–N (Å)	Fe–N–C (°)	$Fe–N_{amide1}$ (Å)	$Fe–N_{amido2}$ (Å)	N–C (Å)	Loewdin spin density (charges)	
							Fe	N
PBE								
quartet	-6	1.71	179	1.90	1.90	1.35	2.65 (-0.24)	-0.10 (0.06)
sextet	0	1.77	180	1.94	1.94	1.33	3.62 (-0.08)	0.50 (0.06)
PBE0								
quartet	-9.8	1.80	179	1.95	1.95	1.32	3.43 (-0.07)	-0.30 (0.05)
sextet	0	1.76	178	1.94	1.94	1.33	3.87 (0.05)	0.52 (0.03)
TPSSh								
quartet	+3.2	1,73	178	1,92	1,92	1,34	3.00 (-0.16)	-0.08 (0.04)
sextet	0	1.76	179	1.94	1.94	1.33	3.75 (0.53)	-0.02 (0.04)
Comparison with experimental values of [4] ($[Fe(NTripp)L_2]^-$)								
		1.775(2)	172.4(2)	1.953(2)	1.972(1)	1.343(2)		

Computational analysis of $[Fe(NTrippp)L_2]$

Table S6. Single point energies, geometries and Loewdin spin densities/atomic charges of **$[Fe(NTripp)L_2]$**.

Spin state	$\Delta E_{q\text{->}t}$ (kJ/mol)	Fe–N (Å)	Fe–N–C (°)	$Fe–N_{amide1}$ (Å)	$Fe–N_{amido2}$ (Å)	N–C (Å)	Loewdin spin density (charges)	
							Fe	N
PBE								
triplet	-9.4	1.66	156	1.83	1.82	1.36	1.84 (-0.21)	+0.03 (0.12)
quintet	0	1.72	180	1.88	1.88	1.34	3.31 (-0.07)	0.22 (0.11)
PBE0								
triplet	+52.0	1.72	150	1,85	1.85	1,32	2.70 (-0.03)	-0.40 (0.14)
quintet	0	1.76	179	1.88	1.88	1.32	3.85 (0.11)	-0.06 (0.08)
TPSSh								
triplet	+17.9	1.67	153	1.84	1.84	1.35	2.21 (0.13)	-0.17 (0.13)
quintet	0	1.74	179	1.88	1.88	1.33	3.55 (0.01)	0.07 (0.11)
Comparison with experimental values of [7] ($[Fe(NXyl)L_2]$)								
		1.749(2)	176.1(2)	1.896(2)	1.904(2)	1.332(2)		

Computational analysis of $[Fe(N_4Ph_2)L_2]^-$ (**[6]$^-$**)

Table S7. Single point energies, geometric features and Loewdin spin densities/atomic charges of **[6]$^-$**.

Spin state	$\Delta E_{sext\text{->}quar}$ (kJ/mol)	$Fe–N_1$ (Å)	$Fe–N_2$ (Å)	$N_1–N_2$ (Å)	$N_2–N_3$ (Å)	$N_3–N_4$ (Å)	Loewdin spin density (charges)	
							Fe	N
**PBE**								
quartet	+5.5	1.91	1.92	1.36	1.27	1.36	2.54 (-0.30)	N_1: 0.02 (0.12)<br>N_2: 0.01 (0.05)<br>N_3: 0.01 (0.05)<br>N_4: 0.02 (0.12)
sextet	0	2.02	2.02	1.36	1.28	1.36	3.77 (-0.11)	N_1: 0.16 (0.09)<br>N_2: 0.04 (0.06)<br>N_3: 0.04 (0.06)<br>N_4: 0.16 (0.09)
**PBE0**								
quartet	+23.7	2.06	2.06	1.31	1.29	1.31	1.40 (-0.12)	N_1: 0.16 (0.09)<br>N_2: 0.04 (0.06)<br>N_3: 0.04 (0.06)<br>N_4: 0.16 (0.09)
sextet	0	2.00	2.00	1.34	1.26	1.35	3.52 (-0.15)	N_1: -0.16 (0.12)<br>N_2: -0.06 (-0.05)<br>N_3: -0.06 (-0.05)<br>N_4: -0.19 (0.12)
**TPSSh**								
quartet	+16.7	1.96	1.96	1.34	1.27	1.34	3.06 (-0.24)	N_1: -0.07 (0.11)<br>N_2: -0.03 (0.05)<br>N_3: -0.03 (0.05)<br>N_4: -0.07 (0.11)
sextet	0	2.00	2.00	1.36	1.27	1.36	3.93 (-0.04)	N_1: 0.16 (0.07)<br>N_2: 0.03 (-0.06)<br>N_3: 0.03 (-0.06)<br>N_4: 0.16 (0.07)
**Experimental values**								
		1.944(2)	1.946(3)	1.357(3)	1.276(3)	1.372(3)		

```
LOWEST ROOT (ROOT 1, MULT 6) =  -3822.152614893 Eh -104006.060 eV
STATE ROOT MULT  DE/a.u.     DE/eV    DE/cm**-1
   1:   0    4   0.001369     0.037    300.4
   2:   0    6   0.019436     0.529   4265.7
   3:   1    4   0.023728     0.646   5207.6
   4:   2    4   0.040917     1.113   8980.2
   5:   4    4   0.042573     1.158   9343.8
   6:   2    6   0.042880     1.167   9411.1
   7:   3    4   0.043856     1.193   9625.4
   8:   0    2   0.054083     1.472  11869.9
```

Figure S 44. Vertical excitation energies of **[6]$^-$** obtained from CASSCF(9,7)/NEVPT2 calculations.

```
ROOT   0:  E=   -3811.6676773053 Eh
      0.52644 [   160]: 2221110
      0.27721 [   150]: 2211111
      0.19370 [   140]: 2201112
```

Figure S 45. Weighted configurations of the quartet ground state of **[6]$^-$** from CASSCF(9,7) calculations.

	198	199	200	201	202	203
	-0.20442	-0.18903	-0.18333	-0.37198	-0.35869	-0.09586
	2.00000	2.00000	2.00000	1.99894	1.99791	1.33328
0 Fe dxz	0.0	0.0	0.6	0.0	0.3	45.7
0 Fe dx2y2	0.0	2.0	0.0	85.7	0.1	0.0
0 Fe dxy	0.0	0.0	0.0	0.0	9.0	1.9
1 N pz	6.4	0.0	0.1	0.1	0.3	11.3
1 N px	0.0	0.1	0.0	0.5	16.9	0.1
3 N pz	6.3	0.0	0.1	0.1	0.4	11.0
3 N px	0.1	0.1	0.0	0.7	19.3	0.0
3 N py	0.0	0.0	0.0	0.3	5.9	0.0
5 N pz	3.8	0.0	0.0	0.0	0.1	5.6
6 C pz	6.2	0.0	0.1	0.0	0.0	0.0
8 C pz	0.0	5.5	8.4	0.0	0.0	0.0
9 N pz	4.0	0.0	0.0	0.0	0.1	5.6
10 C pz	6.9	0.0	0.0	0.0	0.0	0.0
11 C pz	0.0	8.3	5.7	0.0	0.0	0.0
21 C pz	0.0	5.1	3.2	0.1	0.0	0.0
51 C pz	8.1	0.0	0.1	0.0	0.0	0.7
56 C pz	0.0	6.0	8.8	0.0	0.0	0.0
63 C pz	9.2	0.0	0.1	0.0	0.0	0.8
66 C pz	0.0	9.1	6.0	0.0	0.0	0.0

	204	205	206	207	208	209
	0.07278	0.03017	0.03490	0.07930	0.18911	0.20535
	1.00182	1.00021	1.00008	0.66777	0.00000	0.00000
0 Fe dz2	0.5	0.8	89.8	0.0	0.0	0.0
0 Fe dxz	3.8	0.0	0.1	47.4	0.0	0.0
0 Fe dyz	0.0	95.1	0.5	0.0	0.5	0.0
0 Fe dxy	89.7	0.0	0.4	2.0	0.0	0.1
1 N pz	0.0	0.0	0.0	11.7	0.4	0.0
3 N pz	0.0	0.0	0.0	11.5	0.4	0.0
5 N pz	0.0	0.1	0.0	4.0	8.9	0.1
9 N pz	0.0	0.1	0.0	4.0	8.8	0.1
12 C pz	0.0	0.0	0.0	0.8	4.9	6.8
13 C pz	0.0	0.0	0.0	0.8	0.0	8.1
19 C pz	0.0	0.0	0.0	0.9	0.0	5.3
20 C pz	0.0	0.0	0.0	0.9	5.9	4.7
25 C pz	0.0	0.0	0.0	0.0	0.0	9.2
27 C pz	0.0	0.0	0.0	0.0	4.5	6.2
42 C pz	0.0	0.0	0.0	0.0	5.2	4.3
44 C pz	0.0	0.0	0.0	0.0	0.1	5.9

Figure S 46. Loewdin reduced active orbitals of the quartet state of **[6]$^{-}$** from CASSCF(9,7) calculations.

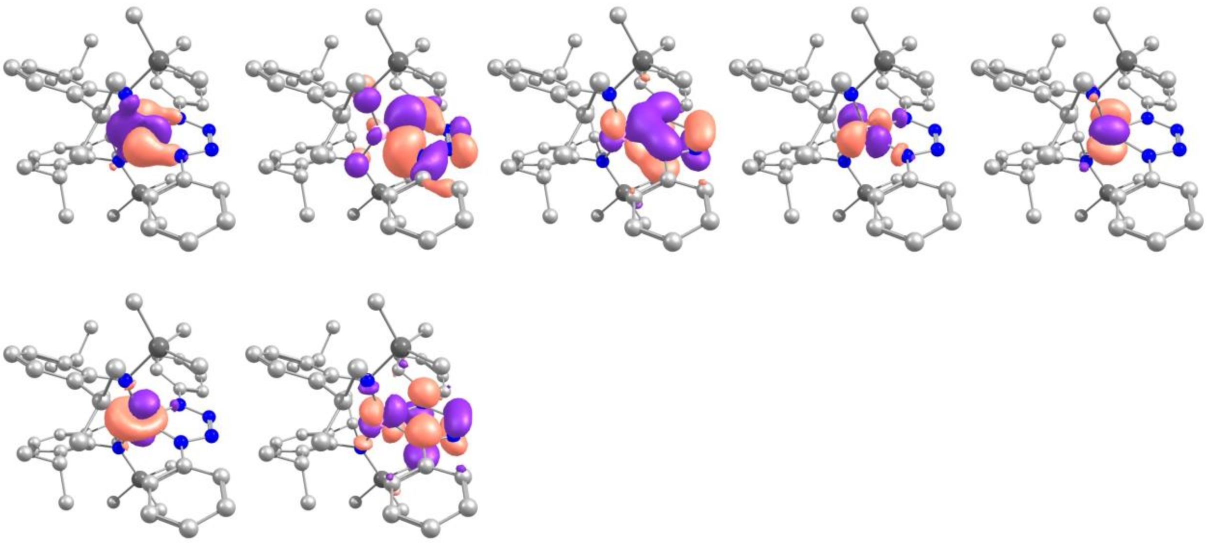

Figure S 47. Depiction of orbitals 168-177 of the quartet ground state o **[6]$^{-}$** from CASSCF(9,7) calculations.

```
---------------------------------------------
CAS-SCF STATES FOR BLOCK  1 MULT= 6 NROOTS= 2
---------------------------------------------

ROOT   0:  E=   -3811.6505856219 Eh
      0.99210 [    20]: 2211111
      0.00744 [    13]: 1212111
ROOT   1:  E=   -3811.6492257427 Eh  0.037 eV    298.5 cm**-1
      0.95119 [    19]: 2121111
      0.03962 [     5]: 1112211
      0.00845 [     9]: 1122111
```

Figure S 48. Weighted configurations of the two lowest configurations of the sextet ground state of **[6]**$^{-}$ from state-averaged CASSCF(9,7) calculations (nroots = 2).

	198	199	200	201	202	203
	-0.20406	-0.18920	-0.18679	-0.34302	-0.19970	-0.16488
	2.00000	2.00000	2.00000	1.97217	1.49996	1.48004
0 Fe dxz	0.0	0.0	0.7	0.2	0.0	61.9
0 Fe dx2y2	0.0	0.2	0.0	0.0	95.8	0.0
0 Fe dxy	0.0	0.0	0.0	11.2	0.0	1.8
1 N pz	5.9	0.1	0.1	0.3	0.0	7.7
1 N px	0.0	0.0	0.1	18.8	0.1	0.0
3 N pz	6.4	0.1	0.0	0.3	0.0	6.6
3 N px	0.1	0.1	0.1	16.1	0.1	0.0
5 N px	0.0	0.0	0.1	6.2	0.1	0.0
6 C pz	5.9	0.0	0.1	0.0	0.0	0.0
8 C pz	0.1	7.7	6.6	0.0	0.0	0.0
9 N px	0.0	0.0	0.1	5.3	0.1	0.0
10 C pz	7.2	0.0	0.0	0.0	0.0	0.0
11 C pz	0.1	6.3	7.8	0.0	0.0	0.0
51 C pz	7.8	0.0	0.1	0.0	0.0	0.5
56 C pz	0.0	8.2	6.8	0.0	0.0	0.0
63 C pz	9.9	0.0	0.1	0.0	0.0	0.5
66 C pz	0.1	6.9	8.2	0.0	0.0	0.0

	204	205	206	207	208	209
	0.02167	-0.02140	0.00115	0.02232	0.19046	0.20606
	1.02777	1.01999	1.00007	1.00001	0.00000	0.00000
0 Fe dz2	0.1	0.0	2.2	88.7	0.0	0.0
0 Fe dxz	1.1	33.1	0.0	0.0	0.0	0.0
0 Fe dyz	0.0	0.0	93.8	1.8	0.5	0.0
0 Fe dxy	89.2	0.0	0.0	0.2	0.0	0.1
1 N pz	0.1	17.0	0.1	0.0	0.3	0.0
3 N pz	0.1	14.8	0.1	0.0	0.4	0.0
5 N pz	0.0	5.3	0.1	0.0	8.9	0.2
9 N pz	0.0	5.4	0.1	0.0	9.1	0.1
12 C pz	0.1	1.2	0.0	0.0	5.0	7.4
13 C pz	0.0	1.2	0.0	0.0	0.0	9.9
20 C pz	0.0	1.2	0.0	0.0	5.7	3.5
25 C pz	0.0	0.0	0.0	0.0	0.0	11.4
27 C pz	0.0	0.0	0.0	0.0	4.8	6.8
42 C pz	0.0	0.0	0.0	0.0	5.2	3.4

Figure S 49. Loewdin reduced active orbitals of the two lowest configurations of the sextet ground state of **[6]**$^{-}$ from state-averaged CASSCF(9,7) calculations (nroots = 2).

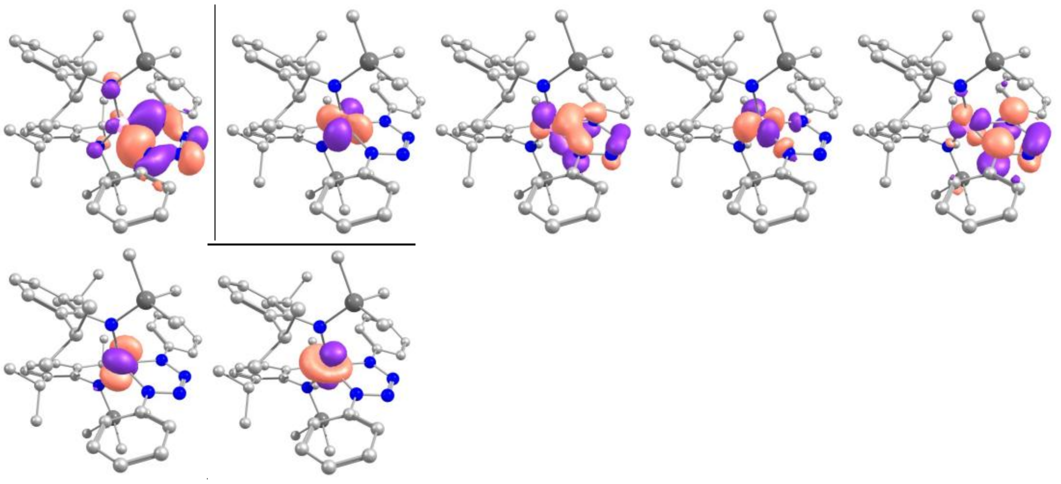

Figure S 50. Depiction of orbitals 168-177 of the two lowest configurations of the sextet ground state of **[6]⁻** from state-averaged CASSCF(9,7) calculations (nroots = 2).

Transition states for Intramolcular C–H amination of $[6]^{0,-}$

Intramolecular HAA by $[6]^{-}$ and subsequent C–N bond formation

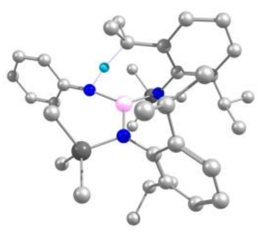	Sextet:<br>5: 0.00 cm**-1<br>***6: -1441.02 cm**-1***<br>7: 21.67 cm**-1<br><br>Quartet:<br>5: 0.00 cm**-1<br>***6: -1378.91 cm**-1***<br>7: 25.75 cm**-1	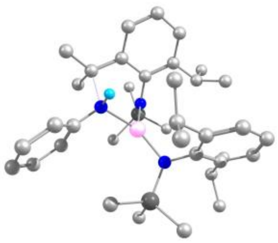	Sextet:<br>No meaningful TS found<br><br>Quartet:<br>5: 0.00 cm**-1<br>***6: -377.61 cm**-1***<br>7: 12.08 cm**-1

Intramolecular HAA by [6] (barrierless C–N bond formation)

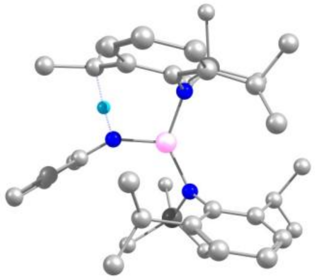	quintet:<br>5: 0.00 cm**-1<br>***6: -1232.35 cm**-1***<br>7: 21.24 cm**-1<br>triplet:<br>5: 0.00 cm**-1<br>***6: -608.83 cm**-1***<br>7: 17.14 cm**-1

Energies of initial H atom transfer for intramolecular C–H amination by $[Fe(NXyl)L_2]^{0,-}$

Intramolecular HAA by $[6]^{-}$

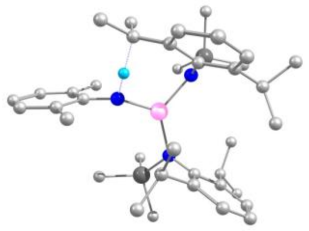	Sextet:<br>5: 0.00 cm**-1<br>***6: -1321.07 cm**-1***<br>7: 23.23 cm**-1<br><br>Quartet:<br>5: 0.00 cm**-1<br>***6: -1004.78 **-1***<br>7: 23.66 cm**-1	***Relativ ground state Energies versus sextet*** ***$[6]^{-}$*** **(kJ/mol)**<br>$\Delta E_{,sext} = 0$<br>$\Delta E_{quar} = +22$	**ΔE_{TS} (vs sextet $[6]^{-}$)**<br>Sextet: 166 kJ/mol<br>Quartet: 126 kJ/mol

Intramolecular HAA by [6]

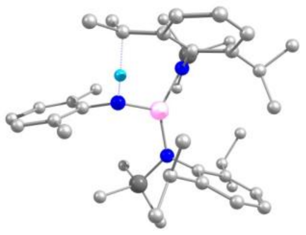	quintet:<br>5: 0.00 cm**-1<br>***6: -1173.31 cm**-1***<br>7: 24.49 cm**-1<br><br>triplet:<br>5: 0.00 cm**-1<br>***6: -277.82 cm**-1***<br>7: 30.35 cm**-1	***Relativ ground state Energies versus quintet [6]*** **(kJ/mol)**<br>$\Delta E_{q} = 0$<br>$\Delta E_{t} = +21$	**ΔE_{TS} (vs. quintet [6])**<br>quintet: 117 kJ/mol<br>triplet: 126 kJ/mol

Selected XYZ–coordinates obtained from geometry optimisation

Imido Complexes:

$[Fe(NPh)L_2]^-$, sextet, PBE functional:

26	2.963583000	5.364531000	13.630092000
7	1.831000000	5.126004000	12.073495000
7	4.618890000	4.766961000	13.440084000
7	2.734668000	6.178812000	15.355225000
14	2.668237000	5.155931000	10.517491000
6	0.603376000	4.455379000	12.230983000
6	5.771884000	4.190193000	13.097113000
14	4.100603000	6.054934000	16.482462000
6	1.643771000	7.011252000	15.662095000
6	1.413808000	5.362216000	9.096756000
6	3.657529000	3.580235000	10.125883000
6	3.862029000	6.627732000	10.510858000
6	0.564130000	3.068222000	12.606787000
6	-0.642061000	5.140541000	12.035298000
6	6.890725000	4.941506000	12.580532000
6	5.979900000	2.766358000	13.221339000
6	5.672264000	6.906004000	15.840655000
6	3.622574000	6.922618000	18.111743000
6	4.503068000	4.238405000	16.849495000
6	1.652541000	8.389810000	15.268462000
6	-0.673590000	2.418760000	12.754271000
6	1.846446000	2.307595000	12.920075000
6	-1.853867000	4.446790000	12.196630000
6	-0.669824000	6.629633000	11.722869000
6	8.089795000	4.321107000	12.225129000
6	7.188061000	2.166133000	12.861706000
6	-0.543785000	7.384177000	16.710135000
6	0.579520000	9.223937000	15.625150000
6	2.796169000	8.948052000	14.432930000
6	-1.884542000	3.090850000	12.546857000
6	2.030216000	2.162250000	14.442112000
6	1.932521000	0.943017000	12.220984000
6	-1.093899000	7.431140000	12.965731000
6	-1.560236000	6.978786000	10.519036000
6	8.262331000	2.928031000	12.358488000
6	0.418815000	5.042905000	16.761286000
6	0.510582000	6.514224000	16.383721000
6	-0.516868000	8.737670000	16.349084000
6	2.326568000	9.264900000	13.002005000
6	3.471100000	10.161970000	15.090844000
6	0.177449000	4.822677000	18.263609000
6	-0.653121000	4.330273000	15.917985000
1	0.586468000	4.636053000	9.198477000
1	1.909555000	5.183204000	8.124397000
1	0.977408000	6.376186000	9.078112000
1	4.402218000	3.367244000	10.912535000
1	4.199021000	3.711002000	9.169460000
1	2.994882000	2.702532000	10.019143000
1	3.317981000	7.583719000	10.609887000
1	4.447143000	6.651762000	9.572940000
1	4.569382000	6.535434000	11.355109000
1	5.934706000	6.491816000	14.852340000
1	6.516536000	6.725611000	16.532530000
1	5.534659000	7.998421000	15.746409000
1	3.365371000	7.983074000	17.935935000
1	4.469808000	6.886081000	18.822006000
1	2.749318000	6.447226000	18.591732000
1	3.629345000	3.712439000	17.275825000
1	5.339704000	4.159566000	17.568762000
1	4.796811000	3.732061000	15.913298000
1	-0.687094000	1.360849000	13.050676000
1	2.687216000	2.925852000	12.556276000
1	-2.799921000	4.986825000	12.052556000
1	0.372368000	6.911268000	11.483386000
1	8.913193000	4.935962000	11.833613000
1	7.297715000	1.077629000	12.973121000
1	-1.409455000	6.989057000	17.259897000
1	0.599171000	10.279384000	15.318855000
1	3.551447000	8.143916000	14.360423000
1	-2.841682000	2.567663000	12.669091000
1	2.015470000	3.148258000	14.942136000
1	2.996910000	1.684655000	14.685662000
1	1.217864000	1.555512000	14.886274000
1	1.175256000	0.232202000	12.604207000
1	2.927135000	0.488121000	12.384109000
1	1.778142000	1.039929000	11.131549000
1	-2.128474000	7.175625000	13.265247000
1	-1.055136000	8.519519000	12.772403000
1	-0.438952000	7.222848000	13.826698000
1	-1.288702000	6.391188000	9.624915000
1	-1.467937000	8.052236000	10.267219000
1	-2.628584000	6.786707000	10.735435000
1	1.399192000	4.600543000	16.505334000
1	-1.347510000	9.403067000	16.617112000
1	1.919720000	8.360288000	12.516594000
1	3.164761000	9.635779000	12.382371000
1	1.532057000	10.035206000	13.001265000
1	2.778228000	11.021005000	15.176396000
1	4.342685000	10.495744000	14.496872000
1	3.824525000	9.917564000	16.108867000
1	-0.805266000	5.220519000	18.581264000
1	0.950796000	5.318900000	18.876621000
1	0.190158000	3.743324000	18.505995000
1	-0.454982000	4.430516000	14.837990000
1	-1.656494000	4.754246000	16.113636000
1	-0.690380000	3.249940000	16.151819000
1	6.769618000	6.026859000	12.469393000
1	5.148704000	2.164221000	13.610167000
1	9.207980000	2.448910000	12.076237000

[Fe(NPh)L$_2$]$^{-}$, quartet, PBE functional:

26	3.184766000	5.171006000	13.619874000
7	1.886471000	4.922179000	12.254410000
7	4.806062000	4.709849000	13.363090000
7	2.722081000	5.980627000	15.264006000
14	2.614839000	4.745418000	10.641125000
6	0.595160000	4.388384000	12.445598000
6	6.106924000	4.452695000	13.149368000
14	4.008259000	5.939846000	16.488228000
6	1.599075000	6.787814000	15.521409000
6	1.256781000	4.437280000	9.335078000
6	3.774537000	3.245335000	10.430504000
6	3.589430000	6.322913000	10.233112000
6	0.394893000	2.982935000	12.649078000
6	-0.553863000	5.243754000	12.378654000
6	6.703298000	4.579589000	11.846061000
6	6.985501000	4.042065000	14.211974000
6	5.616401000	6.791301000	15.939188000
6	3.451164000	6.911040000	18.032936000
6	4.349620000	4.135455000	16.973439000
6	1.616030000	8.182092000	15.188227000
6	-0.911489000	2.462093000	12.661621000
6	1.573636000	2.049021000	12.888823000
6	-1.839216000	4.672203000	12.377422000
6	-0.388095000	6.758162000	12.347726000
6	8.054285000	4.297777000	11.633395000
6	8.339142000	3.789180000	13.980367000
6	-0.591215000	7.112841000	16.574091000
6	0.541945000	9.000036000	15.580157000
6	2.795188000	8.790418000	14.438179000
6	-2.028761000	3.288777000	12.495093000
6	1.682318000	1.680847000	14.379563000
6	1.533250000	0.789922000	12.009250000
6	-1.526615000	7.499929000	13.062034000
6	-0.214752000	7.325417000	10.928201000
6	8.896295000	3.903359000	12.691654000
6	0.358982000	4.763821000	16.505145000
6	0.452126000	6.251267000	16.191098000
6	-0.550615000	8.483681000	16.288963000
6	2.407583000	9.204391000	13.007749000
6	3.431853000	9.962775000	15.202887000
6	0.489013000	4.466343000	18.009616000
6	-0.931232000	4.139655000	15.950822000
1	0.651041000	3.559064000	9.625024000
1	1.741407000	4.212477000	8.366122000
1	0.566268000	5.283419000	9.188571000
1	4.441721000	3.112827000	11.297796000
1	4.404343000	3.382845000	9.530947000
1	3.191353000	2.318263000	10.290855000
1	2.921274000	7.196390000	10.128311000
1	4.165050000	6.207865000	9.295549000
1	4.293796000	6.534925000	11.057708000
1	5.930870000	6.464150000	14.935120000
1	6.433626000	6.562402000	16.649079000
1	5.480376000	7.887885000	15.922595000
1	3.262121000	7.968755000	17.775946000
1	4.256659000	6.880445000	18.790815000
1	2.528966000	6.516334000	18.490136000
1	3.484801000	3.711731000	17.515473000
1	5.245665000	4.037909000	17.614659000
1	4.499338000	3.536139000	16.057986000
1	-1.054534000	1.382269000	12.805121000
1	2.487620000	2.617779000	12.642669000
1	-2.715219000	5.328350000	12.300551000
1	0.547020000	6.961669000	12.900618000
1	8.464260000	4.402509000	10.618852000
1	8.973892000	3.485331000	14.824838000
1	-1.455772000	6.697670000	17.110849000
1	0.569091000	10.071267000	15.335151000
1	3.549991000	7.988088000	14.345310000
1	-3.041037000	2.864220000	12.489988000
1	1.820586000	2.589383000	14.991324000
1	2.552470000	1.021710000	14.558357000
1	0.772516000	1.159086000	14.733762000
1	0.696567000	0.120302000	12.286722000
1	2.469316000	0.211506000	12.118458000
1	1.411973000	1.046592000	10.941495000
1	-2.469762000	7.464854000	12.482381000
1	-1.258998000	8.563598000	13.192505000
1	-1.715985000	7.082777000	14.064824000
1	0.698202000	6.942693000	10.448699000
1	-0.141411000	8.428916000	10.959787000
1	-1.078396000	7.058982000	10.288794000
1	1.218681000	4.295829000	15.990993000
1	-1.373496000	9.140999000	16.598410000
1	2.086756000	8.326184000	12.423353000
1	3.269731000	9.658469000	12.483902000
1	1.580452000	9.940431000	13.007352000
1	2.739427000	10.822432000	15.288546000
1	4.340513000	10.319059000	14.682280000
1	3.719921000	9.667082000	16.227666000
1	-0.320388000	4.956933000	18.584176000
1	1.450483000	4.818052000	18.419313000
1	0.420991000	3.377825000	18.196973000
1	-1.045259000	4.336496000	14.873522000
1	-1.827456000	4.535150000	16.467362000
1	-0.924029000	3.043263000	16.093003000
1	6.068182000	4.908841000	11.017192000
1	6.564981000	3.936301000	15.217414000
1	9.958773000	3.693591000	12.517197000

[Fe(NPh)L$_2$]$^{-}$, sextet, TPSSh functional (used for CASSCF calculations):

26	3.184766000	5.171006000	13.619874000
7	1.886471000	4.922179000	12.254410000
7	4.806062000	4.709849000	13.363090000
7	2.722081000	5.980627000	15.264006000
14	2.614839000	4.745418000	10.641125000
6	0.595160000	4.388384000	12.445598000
6	6.106924000	4.452695000	13.149368000
14	4.008259000	5.939846000	16.488228000
6	1.599075000	6.787814000	15.521409000
6	1.256781000	4.437280000	9.335078000
6	3.774537000	3.245335000	10.430504000
6	3.589430000	6.322913000	10.233112000
6	0.394893000	2.982935000	12.649078000
6	-0.553863000	5.243754000	12.378654000
6	6.703298000	4.579589000	11.846061000
6	6.985501000	4.042065000	14.211974000
6	5.616401000	6.791301000	15.939188000
6	3.451164000	6.911040000	18.032936000
6	4.349620000	4.135455000	16.973439000
6	1.616030000	8.182092000	15.188227000
6	-0.911489000	2.462093000	12.661621000
6	1.573636000	2.049021000	12.888823000
6	-1.839216000	4.672203000	12.377422000
6	-0.388095000	6.758162000	12.347726000
6	8.054285000	4.297777000	11.633395000
6	8.339142000	3.789180000	13.980367000
6	-0.591215000	7.112841000	16.574091000
6	0.541945000	9.000036000	15.580157000
6	2.795188000	8.790418000	14.438179000
6	-2.028761000	3.288777000	12.495093000
6	1.682318000	1.680847000	14.379563000
6	1.533250000	0.789922000	12.009250000
6	-1.526615000	7.499929000	13.062034000
6	-0.214752000	7.325417000	10.928201000
6	8.896295000	3.903359000	12.691654000
6	0.358982000	4.763821000	16.505145000
6	0.452126000	6.251267000	16.191098000
6	-0.550615000	8.483681000	16.288963000
6	2.407583000	9.204391000	13.007749000
6	3.431853000	9.962775000	15.202887000
6	0.489013000	4.466343000	18.009616000
6	-0.931232000	4.139655000	15.950822000
1	0.651041000	3.559064000	9.625024000
1	1.741407000	4.212477000	8.366122000
1	0.566268000	5.283419000	9.188571000
1	4.441721000	3.112827000	11.297796000
1	4.404343000	3.382845000	9.530947000
1	3.191353000	2.318263000	10.290855000
1	2.921274000	7.196390000	10.128311000
1	4.165050000	6.207865000	9.295549000
1	4.293796000	6.534925000	11.057708000
1	5.930870000	6.464150000	14.935120000
1	6.433626000	6.562402000	16.649079000
1	5.480376000	7.887885000	15.922595000
1	3.262121000	7.968755000	17.775946000
1	4.256659000	6.880445000	18.790815000
1	2.528966000	6.516334000	18.490136000
1	3.484801000	3.711731000	17.515473000
1	5.245665000	4.037909000	17.614659000
1	4.499338000	3.536139000	16.057986000
1	-1.054534000	1.382269000	12.805121000
1	2.487620000	2.617779000	12.642669000
1	-2.715219000	5.328350000	12.300551000
1	0.547020000	6.961669000	12.900618000
1	8.464260000	4.402509000	10.618852000
1	8.973892000	3.485331000	14.824838000
1	-1.455772000	6.697670000	17.110849000
1	0.569091000	10.071267000	15.335151000
1	3.549991000	7.988088000	14.345310000
1	-3.041037000	2.864220000	12.489988000
1	1.820586000	2.589383000	14.991324000
1	2.552470000	1.021710000	14.558357000
1	0.772516000	1.159086000	14.733762000
1	0.696567000	0.120302000	12.286722000
1	2.469316000	0.211506000	12.118458000
1	1.411973000	1.046592000	10.941495000
1	-2.469762000	7.464854000	12.482381000
1	-1.258998000	8.563598000	13.192505000
1	-1.715985000	7.082777000	14.064824000
1	0.698202000	6.942693000	10.448699000
1	-0.141411000	8.428916000	10.959787000
1	-1.078396000	7.058982000	10.288794000
1	1.218681000	4.295829000	15.990993000
1	-1.373496000	9.140999000	16.598410000
1	2.086756000	8.326184000	12.423353000
1	3.269731000	9.658469000	12.483902000
1	1.580452000	9.940431000	13.007352000
1	2.739427000	10.822432000	15.288546000
1	4.340513000	10.319059000	14.682280000
1	3.719921000	9.667082000	16.227666000
1	-0.320388000	4.956933000	18.584176000
1	1.450483000	4.818052000	18.419313000
1	0.420991000	3.377825000	18.196973000
1	-1.045259000	4.336496000	14.873522000
1	-1.827456000	4.535150000	16.467362000
1	-0.924029000	3.043263000	16.093003000
1	6.068182000	4.908841000	11.017192000
1	6.564981000	3.936301000	15.217414000
1	9.958773000	3.693591000	12.517197000

[Fe(NPh)L$_2$], quintet, PBE functional (used for casscf calculations):

26	3.037889000	5.214860000	13.592075000
7	1.816093000	5.160134000	12.169260000
7	4.577797000	4.470030000	13.424157000
7	2.768385000	6.182470000	15.149602000
14	2.651527000	5.356745000	10.594726000
6	0.583771000	4.476309000	12.289326000
6	5.791058000	3.906196000	13.264315000
14	4.144209000	6.169877000	16.329003000
6	1.637034000	6.963104000	15.482301000
6	1.417093000	5.712715000	9.206270000
6	3.617387000	3.802087000	10.100378000
6	3.846789000	6.813276000	10.785414000
6	0.555423000	3.071401000	12.586334000
6	-0.656693000	5.170241000	12.100408000
6	6.854515000	4.598309000	12.598921000
6	6.055659000	2.580994000	13.739479000
6	5.706559000	6.896024000	15.547446000
6	3.656498000	7.230171000	17.821245000
6	4.464315000	4.397230000	16.906062000
6	1.595235000	8.342917000	15.110845000
6	-0.682117000	2.413686000	12.692932000
6	1.828760000	2.268117000	12.819270000
6	-1.864952000	4.460631000	12.211464000
6	-0.711980000	6.667409000	11.834256000
6	8.101136000	3.994773000	12.423031000
6	7.308919000	1.992629000	13.559777000
6	-0.455206000	7.248972000	16.706786000
6	0.547177000	9.145725000	15.591751000
6	2.657374000	8.955181000	14.207428000
6	-1.890515000	3.092914000	12.506270000
6	2.022552000	1.964017000	14.316691000
6	1.880188000	0.976514000	11.986871000
6	-1.228398000	7.411851000	13.077182000
6	-1.564970000	7.029284000	10.605598000
6	8.338800000	2.692588000	12.902784000
6	0.544942000	4.924964000	16.611252000
6	0.578270000	6.405861000	16.262432000
6	-0.468486000	8.614114000	16.396005000
6	2.037067000	9.493296000	12.905675000
6	3.470932000	10.047901000	14.923265000
6	0.553538000	4.676937000	18.129277000
6	-0.652388000	4.227906000	15.941545000
1	0.594525000	4.975471000	9.204817000
1	1.941188000	5.637275000	8.235747000
1	0.980945000	6.722485000	9.281433000
1	4.368578000	3.527664000	10.859972000
1	4.145694000	3.985014000	9.145964000
1	2.940731000	2.942637000	9.949932000
1	3.299371000	7.754529000	10.964634000
1	4.450519000	6.932710000	9.867094000
1	4.545329000	6.650284000	11.626415000
1	6.048008000	6.286024000	14.695549000
1	6.518138000	6.923619000	16.297965000
1	5.533693000	7.927585000	15.194423000
1	3.410467000	8.265832000	17.530265000
1	4.501848000	7.261592000	18.533101000
1	2.779333000	6.816308000	18.346883000
1	3.575640000	3.979346000	17.411802000
1	5.306924000	4.375972000	17.621517000
1	4.720758000	3.748080000	16.052323000
1	-0.697640000	1.341896000	12.928797000
1	2.681730000	2.896069000	12.504826000
1	-2.811896000	4.997263000	12.070454000
1	0.327872000	6.997097000	11.653127000
1	8.900582000	4.541563000	11.907228000
1	7.489926000	0.976345000	13.931941000
1	-1.266472000	6.825844000	17.313576000
1	0.522294000	10.210537000	15.325013000
1	3.355141000	8.141346000	13.934239000
1	-2.846045000	2.561499000	12.593780000
1	2.094216000	2.891065000	14.915127000
1	2.950337000	1.387193000	14.485622000
1	1.176795000	1.376679000	14.719705000
1	1.120187000	0.243227000	12.314581000
1	2.868977000	0.493032000	12.086950000
1	1.706404000	1.178739000	10.915363000
1	-2.275790000	7.130981000	13.295303000
1	-1.196811000	8.506055000	12.924944000
1	-0.625669000	7.177007000	13.966693000
1	-1.262273000	6.468044000	9.706126000
1	-1.478819000	8.108841000	10.383334000
1	-2.636167000	6.821227000	10.784062000
1	1.467916000	4.480927000	16.195083000
1	-1.277214000	9.257383000	16.763557000
1	1.476386000	8.703185000	12.377874000
1	2.822174000	9.874645000	12.227196000
1	1.336884000	10.325574000	13.105026000
1	2.830377000	10.900963000	15.214764000
1	4.268009000	10.439288000	14.264499000
1	3.947436000	9.663152000	15.841656000
1	-0.353081000	5.087817000	18.610980000
1	1.428183000	5.142625000	18.616234000
1	0.580891000	3.593252000	18.346114000
1	-0.642886000	4.368552000	14.848379000
1	-1.610579000	4.626209000	16.323654000
1	-0.636008000	3.141377000	16.142680000
1	6.659446000	5.610070000	12.224169000
1	5.243984000	2.043380000	14.243320000
1	9.320744000	2.225062000	12.763145000

[Fe(NPh)L$_2$], triplet, PBE functional:

26	3.008057000	5.006635000	13.654776000
7	1.932207000	4.544082000	12.246875000
7	4.593609000	4.763221000	13.243712000
7	2.763526000	5.775208000	15.274583000
14	2.603060000	4.172334000	10.620711000
6	0.557998000	4.308013000	12.488718000
6	5.724890000	5.383996000	12.794963000
14	3.944614000	5.362302000	16.588008000
6	1.820166000	6.805629000	15.525918000
6	1.185559000	3.560043000	9.519280000
6	3.895042000	2.791853000	10.707820000
6	3.385293000	5.722799000	9.870594000
6	0.115116000	3.014584000	12.913285000
6	-0.400303000	5.329761000	12.194879000
6	5.761341000	6.743107000	12.371305000
6	6.932405000	4.626820000	12.749465000
6	5.729427000	5.798666000	16.129670000
6	3.524103000	6.413326000	18.109253000
6	3.849463000	3.505080000	16.924604000
6	2.144541000	8.173611000	15.271555000
6	-1.263896000	2.745933000	12.939934000
6	1.106991000	1.922811000	13.294185000
6	-1.767360000	5.003593000	12.224934000
6	0.034428000	6.743683000	11.833188000
6	6.957931000	7.318238000	11.937143000
6	8.121134000	5.212780000	12.305120000
6	-0.287973000	7.515443000	16.532302000
6	1.244908000	9.174852000	15.673881000
6	3.433637000	8.565917000	14.566924000
6	-2.203163000	3.718891000	12.574833000
6	0.964225000	1.521606000	14.773386000
6	0.994242000	0.691284000	12.378723000
6	-0.617256000	7.790342000	12.752647000
6	-0.239013000	7.079383000	10.355752000
6	8.143292000	6.559865000	11.901101000
6	0.142296000	5.027890000	16.360487000
6	0.567238000	6.472635000	16.133398000
6	0.042994000	8.858479000	16.317817000
6	3.134726000	9.163090000	13.179697000
6	4.304664000	9.513274000	15.408509000
6	0.263960000	4.599554000	17.834895000
6	-1.285067000	4.766182000	15.856353000
1	0.733831000	2.637658000	9.923788000
1	1.588719000	3.329265000	8.515764000
1	0.375010000	4.298424000	9.401865000
1	4.707886000	3.044982000	11.407631000
1	4.333440000	2.633790000	9.704904000
1	3.439921000	1.841092000	11.036146000
1	2.697859000	6.584790000	9.923750000
1	3.633980000	5.544835000	8.808011000
1	4.317343000	5.990956000	10.395525000
1	6.176697000	5.070079000	15.435654000
1	6.330384000	5.801086000	17.058441000
1	5.809058000	6.800391000	15.673552000
1	3.741354000	7.476118000	17.900704000
1	4.164968000	6.095266000	18.952246000
1	2.472291000	6.349638000	18.429426000
1	2.848945000	3.190053000	17.266444000
1	4.587701000	3.214014000	17.694452000
1	4.084873000	2.954586000	15.995821000
1	-1.610595000	1.748128000	13.239903000
1	2.117890000	2.353363000	13.166527000
1	-2.506964000	5.773703000	11.969611000
1	1.128481000	6.783246000	11.994559000
1	6.968643000	8.367614000	11.616364000
1	9.041220000	4.616046000	12.275340000
1	-1.242279000	7.266786000	17.013321000
1	1.495116000	10.226791000	15.483578000
1	4.005838000	7.634108000	14.412896000
1	-3.274442000	3.483295000	12.584645000
1	1.122776000	2.388812000	15.436493000
1	1.709605000	0.750914000	15.042000000
1	-0.039303000	1.109588000	14.988989000
1	0.011861000	0.194556000	12.485832000
1	1.771899000	-0.052913000	12.630622000
1	1.114730000	0.962063000	11.315466000
1	-1.713291000	7.829509000	12.609572000
1	-0.219703000	8.797794000	12.532965000
1	-0.421287000	7.574665000	13.814763000
1	0.262943000	6.375790000	9.670919000
1	0.119025000	8.097984000	10.117435000
1	-1.322090000	7.045734000	10.133489000
1	0.831918000	4.402318000	15.764494000
1	-0.640777000	9.654508000	16.636944000
1	2.599070000	8.437041000	12.541976000
1	4.068324000	9.455167000	12.663400000
1	2.504185000	10.067652000	13.259600000
1	3.810424000	10.488604000	15.573289000
1	5.266872000	9.709570000	14.900608000
1	4.527035000	9.081583000	16.400296000
1	-0.378186000	5.227757000	18.480439000
1	1.295953000	4.677918000	18.212465000
1	-0.061479000	3.549996000	17.958558000
1	-1.412407000	5.096884000	14.813886000
1	-2.037999000	5.286835000	16.477031000
1	-1.515438000	3.686929000	15.897611000
1	4.830470000	7.317041000	12.391557000
1	6.898203000	3.580439000	13.074839000
1	9.078871000	7.016149000	11.556472000

Amido Complexes/Insertion products

[Fe(N{$SiMe_3$})(6-{CMe_2(NHPh)}-2-^{i}Pr-Ph)L]$^-$,
quartet:

26	3.795580000	2.196726000	-0.690486000
7	3.024149000	0.508754000	-0.994627000
7	0.993683000	-0.835298000	2.757193000
7	4.284870000	3.992241000	-0.424213000
14	3.891900000	-0.732140000	-1.881151000
6	1.627407000	0.526041000	-0.787562000
6	0.625900000	-2.163617000	2.697903000
14	5.534582000	4.414518000	0.723987000
6	3.502481000	4.940588000	-1.113592000
6	4.365563000	-0.299527000	-3.682152000
6	2.825451000	-2.310936000	-1.966459000
6	5.553840000	-1.118563000	-1.036270000
6	1.029209000	0.153286000	0.468265000
6	0.746192000	0.942719000	-1.849916000
6	0.559057000	-2.893113000	1.479273000
6	0.249434000	-2.840187000	3.891117000
6	7.106263000	3.386869000	0.416009000
6	5.953079000	6.265973000	0.547085000
6	5.029843000	4.114073000	2.538891000
6	3.950911000	5.473460000	-2.368459000
6	-0.372771000	0.187684000	0.601982000
6	1.832910000	-0.116365000	1.770031000
6	-0.644582000	0.943237000	-1.665558000
6	1.301074000	1.458692000	-3.171695000
6	0.144889000	-4.230692000	1.474101000
6	-0.169128000	-4.174379000	3.868017000
6	1.486382000	6.327676000	-1.315415000
6	3.156720000	6.404679000	-3.056384000
6	5.241006000	4.949863000	-2.981935000
6	-1.216985000	0.558550000	-0.448474000
6	2.131558000	1.264093000	2.390575000
6	3.167903000	-0.875058000	1.621578000
6	1.015071000	2.959743000	-3.354649000
6	0.801626000	0.639966000	-4.373030000
6	-0.223554000	-4.890363000	2.659013000
6	1.716215000	4.855200000	0.729396000
6	2.247481000	5.393059000	-0.591126000
6	1.926801000	6.840364000	-2.541784000
6	4.934127000	3.720513000	-3.855885000
6	6.046938000	6.005289000	-3.749906000
6	1.575048000	5.967201000	1.782556000
6	0.402277000	4.078701000	0.540947000
1	3.489258000	-0.217146000	-4.348033000
1	5.027976000	-1.088533000	-4.088322000
1	4.913644000	0.659071000	-3.717756000
1	2.680592000	-2.765615000	-0.970366000
1	3.294976000	-3.066449000	-2.623297000
1	1.825568000	-2.073466000	-2.374741000
1	6.131003000	-0.182256000	-0.919624000
1	6.148783000	-1.816737000	-1.655243000
1	5.424046000	-1.565185000	-0.035621000
1	6.867788000	2.310501000	0.500501000
1	7.902939000	3.629993000	1.145056000
1	7.496018000	3.562143000	-0.603071000
1	6.342697000	6.494822000	-0.461364000
1	6.712426000	6.572250000	1.290451000
1	5.049693000	6.884663000	0.699311000
1	4.154301000	4.726560000	2.820308000
1	5.860001000	4.360741000	3.228558000
1	4.761818000	3.052056000	2.686052000
1	-0.800450000	-0.088453000	1.572332000
1	1.092920000	-0.470961000	3.702369000
1	-1.291587000	1.268900000	-2.492470000
1	2.397419000	1.353062000	-3.111565000
1	0.107327000	-4.763512000	0.514788000
1	-0.449815000	-4.662937000	4.810786000
1	0.519598000	6.656327000	-0.907718000
1	3.503552000	6.798824000	-4.021287000
1	5.858658000	4.586714000	-2.139701000
1	-2.305844000	0.559641000	-0.311543000
1	2.706717000	1.872402000	1.663425000
1	2.739679000	1.164557000	3.311374000
1	1.193453000	1.797241000	2.624307000
1	3.050354000	-1.828958000	1.083051000
1	3.551707000	-1.100850000	2.634404000
1	3.895853000	-0.256887000	1.079681000
1	-0.071388000	3.168764000	-3.383087000
1	1.458161000	3.332042000	-4.297318000
1	1.453197000	3.551866000	-2.532307000
1	1.024047000	-0.435818000	-4.250622000
1	1.279698000	0.985264000	-5.309584000
1	-0.293251000	0.737727000	-4.504677000
1	2.473380000	4.134278000	1.088061000
1	1.318639000	7.569075000	-3.093325000
1	4.407162000	2.954352000	-3.253238000
1	5.860086000	3.262659000	-4.253601000
1	4.284738000	3.993022000	-4.709491000
1	5.521988000	6.346055000	-4.662780000
1	7.019395000	5.588709000	-4.072979000
1	6.244273000	6.896421000	-3.125845000
1	0.817641000	6.716645000	1.481801000
1	2.530680000	6.503142000	1.929872000
1	1.261972000	5.548775000	2.758329000
1	0.544602000	3.215627000	-0.132580000
1	-0.391899000	4.718155000	0.110567000
1	0.031600000	3.685798000	1.506062000
1	0.810837000	-2.395305000	0.538254000
1	0.294498000	-2.296122000	4.845430000
1	-0.546564000	-5.938245000	2.641939000

[Fe(N{SiMe$_3$)(6-{CMe$_2$(NHPh)}-2-^{i}Pr-Ph)L], quintet:

26	-0.289556000	-0.158864000	-0.097018000
7	-0.785422000	-1.981945000	-0.117641000
7	0.626178000	-0.337376000	1.905498000
7	-0.585562000	1.620579000	-0.647144000
14	-0.323499000	-3.223552000	-1.299602000
6	-1.526834000	-2.243444000	1.056959000
6	1.894120000	0.335921000	1.956185000
14	0.636339000	2.770127000	-1.235542000
6	-1.951321000	2.005286000	-0.572008000
6	-1.490295000	-3.513207000	-2.773760000
6	-0.185680000	-4.899097000	-0.418389000
6	1.334301000	-2.672178000	-2.048288000
6	-0.898699000	-2.204592000	2.352843000
6	-2.933293000	-2.510930000	0.990822000
6	2.871851000	0.071018000	0.976408000
6	2.161470000	1.290809000	2.957329000
6	1.891499000	1.830935000	-2.303682000
6	-0.163640000	4.171825000	-2.237460000
6	1.553856000	3.614499000	0.192703000
6	-2.768802000	2.014571000	-1.748939000
6	-1.653594000	-2.544794000	3.490547000
6	0.559639000	-1.743072000	2.524720000
6	-3.638627000	-2.843007000	2.158706000
6	-3.703163000	-2.336469000	-0.309285000
6	4.104556000	0.737919000	1.014069000
6	3.393614000	1.959342000	2.987690000
6	-3.887146000	2.764338000	0.726764000
6	-4.114234000	2.405416000	-1.650832000
6	-2.204494000	1.526300000	-3.075822000
6	-3.007146000	-2.890221000	3.404413000
6	0.974284000	-1.615730000	4.002798000
6	1.558604000	-2.692345000	1.849313000
6	-4.552908000	-1.053020000	-0.244229000
6	-4.556587000	-3.561265000	-0.673074000
6	4.372742000	1.679526000	2.020895000
6	-1.709943000	2.388399000	1.957354000
6	-2.532461000	2.389860000	0.676640000
6	-4.680789000	2.780250000	-0.425028000
6	-2.388461000	0.004477000	-3.193458000
6	-2.769675000	2.250368000	-4.304888000
6	-1.650196000	3.777011000	2.614675000
6	-2.224702000	1.322282000	2.943651000
1	-2.445624000	-3.974914000	-2.472983000
1	-0.985590000	-4.214455000	-3.465505000
1	-1.711220000	-2.590265000	-3.334147000
1	0.611349000	-4.921018000	0.342596000
1	0.016692000	-5.698299000	-1.154486000
1	-1.139792000	-5.137520000	0.086746000
1	1.236169000	-1.661507000	-2.487589000
1	1.647510000	-3.360832000	-2.854524000
1	2.146231000	-2.638181000	-1.300005000
1	2.405739000	1.048049000	-1.718592000
1	2.662864000	2.518487000	-2.697355000
1	1.394155000	1.343101000	-3.161461000
1	-0.512230000	3.853767000	-3.234206000
1	0.578399000	4.978951000	-2.379867000
1	-1.027578000	4.597990000	-1.696310000
1	0.851005000	4.174505000	0.835176000
1	2.292528000	4.334403000	-0.206880000
1	2.093242000	2.892007000	0.824157000
1	-1.182841000	-2.542144000	4.477999000
1	-0.049811000	0.215247000	2.448011000
1	-4.713692000	-3.055311000	2.089500000
1	-2.958948000	-2.189086000	-1.108386000
1	4.854147000	0.529396000	0.241343000
1	3.586189000	2.705535000	3.767694000
1	-4.328235000	3.053199000	1.690282000
1	-4.739416000	2.410195000	-2.552212000
1	-1.114938000	1.704609000	-3.045765000
1	-3.565982000	-3.163705000	4.307271000
1	0.313018000	-0.933568000	4.566547000
1	2.005059000	-1.228506000	4.069060000
1	0.957240000	-2.599938000	4.499927000
1	1.409188000	-3.704819000	2.259882000
1	2.600414000	-2.391618000	2.054431000
1	1.398815000	-2.735005000	0.764672000
1	-5.310299000	-1.116066000	0.559315000
1	-5.084194000	-0.882199000	-1.198393000
1	-3.927467000	-0.165243000	-0.047025000
1	-3.950447000	-4.484630000	-0.707442000
1	-5.031083000	-3.425235000	-1.662201000
1	-5.369351000	-3.726540000	0.058494000
1	-0.678768000	2.125322000	1.652681000
1	-5.734729000	3.078971000	-0.370026000
1	-1.925491000	-0.510130000	-2.332491000
1	-1.920525000	-0.382424000	-4.118092000
1	-3.459890000	-0.267905000	-3.208775000
1	-3.837152000	2.013785000	-4.470004000
1	-2.226969000	1.940060000	-5.216268000
1	-2.680976000	3.347147000	-4.208589000
1	-2.648497000	4.108121000	2.955953000
1	-1.272855000	4.536479000	1.907452000
1	-0.982924000	3.769521000	3.497023000
1	-2.276135000	0.319426000	2.480405000
1	-3.245664000	1.562729000	3.293330000
1	-1.584823000	1.260763000	3.846474000
1	2.650306000	-0.645210000	0.176380000
1	1.395009000	1.510512000	3.711525000
1	5.335259000	2.203662000	2.043402000

Tetrazenide Complex [6]$^-$, sextet, PBE functional (used for CASSCF calculations):

26	6.767446000	8.540192000	23.716489000
7	4.908562000	7.776420000	23.949083000
7	7.081379000	9.694209000	25.243862000
7	7.080365000	6.610675000	24.245146000
7	7.234098000	8.655888000	21.833115000
7	4.848512000	6.477256000	24.332932000
6	3.667560000	8.404734000	23.888657000
14	6.853321000	9.001566000	26.883592000
6	7.376494000	11.076091000	25.168574000
7	5.963857000	5.870667000	24.458615000
6	8.265065000	5.886074000	24.342539000
6	8.195700000	9.534124000	21.278896000
6	2.506563000	7.881552000	24.520455000
6	3.538057000	9.602689000	23.145073000
6	7.447441000	10.288365000	28.167763000
6	5.067203000	8.448174000	27.234600000
6	7.883965000	7.454481000	27.277810000
6	8.702104000	11.521141000	24.846128000
6	6.383515000	12.071486000	25.468126000
6	9.493907000	6.583152000	24.441004000
6	8.299839000	4.464995000	24.378229000
6	9.478754000	9.058028000	20.837334000
6	7.891721000	10.924074000	21.088286000
6	6.928516000	5.682115000	21.037943000
1	2.604649000	6.952000000	25.088968000
6	1.277181000	8.537743000	24.398631000
1	4.423766000	9.995941000	22.635440000
6	2.300993000	10.242733000	23.019433000
1	8.552290000	10.296711000	28.197984000
1	7.087047000	9.996556000	29.172184000
1	7.119229000	11.319594000	27.961534000
1	4.293281000	9.171384000	26.932889000
1	4.959563000	8.256575000	28.319538000
1	4.865322000	7.501053000	26.704200000
1	7.462049000	6.550117000	26.811979000
1	7.851812000	7.322583000	28.376903000
1	8.941910000	7.528614000	26.976421000
6	9.785170000	10.526304000	24.454437000
6	9.025506000	12.885065000	24.952428000
6	4.909416000	11.726719000	25.665055000
6	6.764010000	13.421705000	25.577008000
1	9.474707000	7.678311000	24.429289000
6	10.702084000	5.895538000	24.595227000
1	7.350751000	3.925540000	24.308137000
6	9.515959000	3.788353000	24.517951000
6	10.330361000	9.918013000	20.119686000
6	9.973287000	7.642050000	21.120642000
6	8.786463000	11.744333000	20.378946000
1	6.643070000	5.067025000	20.163258000
1	7.997014000	5.515732000	21.246155000
1	6.358918000	5.315631000	21.909987000
6	1.155674000	9.720961000	23.646344000
1	2.230937000	11.159723000	22.419804000
1	9.268849000	9.691322000	23.938972000
6	10.817417000	11.106433000	23.479164000
6	10.481330000	9.949632000	25.700492000
6	8.077892000	13.838080000	25.345586000
1	10.049303000	13.208704000	24.729255000
1	4.785955000	10.661562000	25.399266000
6	4.427274000	11.930257000	27.114326000
6	4.011213000	12.547999000	24.723153000
1	6.002034000	14.167473000	25.841997000
1	11.634120000	6.466867000	24.692680000
6	10.729985000	4.490271000	24.633422000
1	9.515938000	2.689928000	24.540837000
1	11.292648000	9.530508000	19.758180000
6	9.990928000	11.249281000	19.864874000
1	9.248915000	7.176633000	21.812627000
6	11.341236000	7.653941000	21.825229000
6	10.071966000	6.769541000	19.854767000
1	8.529395000	12.798176000	20.217736000
1	0.186103000	10.224732000	23.547678000
1	10.331271000	11.564176000	22.601783000
1	11.488047000	10.311727000	23.110852000
1	11.454707000	11.868602000	23.966448000
1	10.984899000	10.755923000	26.266675000
1	11.246033000	9.200978000	25.419399000
1	9.759701000	9.460919000	26.373679000
1	8.356096000	14.895362000	25.441587000
1	4.983534000	11.311315000	27.835385000
1	3.355754000	11.670793000	27.203566000
1	4.543654000	12.988009000	27.419596000
1	4.063843000	13.628611000	24.955257000
1	2.959206000	12.227847000	24.819808000
1	4.303549000	12.413958000	23.669378000
1	11.678965000	3.953220000	24.754107000
1	10.666786000	11.901887000	19.297700000
1	12.126028000	8.094810000	21.182120000
1	11.648518000	6.624830000	22.081191000
1	11.308230000	8.234607000	22.761479000
1	9.103520000	6.655813000	19.343297000
1	10.436943000	5.757865000	20.112952000
1	10.784039000	7.209238000	19.130058000
14	6.451883000	7.495436000	20.709417000
1	0.394758000	8.116267000	24.899317000
6	4.552945000	7.463343000	20.746661000
1	4.229242000	6.884255000	19.859747000
1	4.166720000	6.957638000	21.645539000
1	4.084462000	8.459790000	20.688206000
6	6.859018000	8.022310000	18.916640000
1	7.905235000	8.328123000	18.756764000
1	6.623361000	7.190145000	18.226898000
1	6.221928000	8.880675000	18.635212000
6	6.576762000	11.514037000	21.578595000
1	6.285855000	10.934199000	22.477749000
6	6.691535000	12.986801000	21.993645000
6	5.468459000	11.335811000	20.524481000
1	5.739448000	11.866056000	19.591853000
1	5.312777000	10.273689000	20.276974000
1	4.506053000	11.748012000	20.882701000
1	7.520839000	13.145104000	22.702741000
1	5.763926000	13.321983000	22.487615000
1	6.847888000	13.645681000	21.118346000

Tetrazenide Complex $[6]^-$, quartet, PBE functional:

26	6.738393000	8.447362000	23.750352000
7	4.971428000	7.756240000	24.005786000
7	6.983530000	9.709566000	25.197187000
7	7.072475000	6.611801000	24.193431000
7	7.288833000	8.612752000	21.898302000
7	4.875817000	6.451298000	24.381320000
6	3.743315000	8.425351000	23.967163000
14	6.749094000	9.033741000	26.845470000
6	7.309229000	11.085113000	25.139983000
7	5.978084000	5.830838000	24.409260000
6	8.283468000	5.913477000	24.244053000
6	8.231154000	9.496442000	21.319993000
6	2.577293000	7.916335000	24.597521000
6	3.631537000	9.616490000	23.214466000
6	7.408986000	10.294057000	28.124800000
6	4.957136000	8.535462000	27.255527000
6	7.728334000	7.447865000	27.220300000
6	8.648974000	11.505378000	24.839086000
6	6.341245000	12.101597000	25.453717000
6	9.491081000	6.633325000	24.393548000
6	8.346552000	4.494645000	24.219730000
6	9.509222000	9.032378000	20.849416000
6	7.906823000	10.881159000	21.117374000
6	7.008771000	5.634222000	21.011517000
1	2.659714000	6.986424000	25.167537000
6	1.354119000	8.582463000	24.466996000
1	4.525182000	9.991066000	22.707436000
6	2.400153000	10.265982000	23.078467000
1	8.513640000	10.269328000	28.136021000
1	7.056448000	10.006226000	29.133192000
1	7.108779000	11.336050000	27.929910000
1	4.189940000	9.270663000	26.967110000
1	4.879089000	8.361747000	28.346000000
1	4.720990000	7.584964000	26.746467000
1	7.281094000	6.563899000	26.738909000
1	7.689538000	7.299066000	28.317027000
1	8.788605000	7.497291000	26.921918000
6	9.720618000	10.496064000	24.454868000
6	9.002791000	12.860133000	24.967183000
6	4.860934000	11.791230000	25.660172000
6	6.750585000	13.441622000	25.578511000
1	9.441214000	7.725585000	24.428768000
6	10.711347000	5.964723000	24.539381000
1	7.411975000	3.935821000	24.116776000
6	9.574448000	3.837729000	24.350510000
6	10.340059000	9.897181000	20.114037000
6	10.021374000	7.618537000	21.111358000
6	8.779495000	11.705194000	20.384571000
1	6.725212000	5.060467000	20.108486000
1	8.079549000	5.467408000	21.205812000
1	6.446122000	5.217839000	21.865359000
6	1.248185000	9.760455000	23.705085000
1	2.339619000	11.175658000	22.467383000
1	9.194506000	9.669944000	23.936255000
6	10.770992000	11.064782000	23.491976000
6	10.403241000	9.912193000	25.705628000
6	8.074534000	13.831637000	25.359517000
1	10.036921000	13.161575000	24.760871000
1	4.711052000	10.731560000	25.387913000
6	4.398941000	11.999787000	27.115393000
6	3.973844000	12.639908000	24.732318000
1	6.004094000	14.200548000	25.850306000
1	11.628282000	6.550817000	24.680307000
6	10.769703000	4.560782000	24.516826000
1	9.597643000	2.739757000	24.327366000
1	11.298378000	9.516496000	19.735040000
6	9.983025000	11.223302000	19.857493000
1	9.314450000	7.141838000	21.813302000
6	11.404337000	7.635354000	21.785810000
6	10.103306000	6.760550000	19.834091000
1	8.504874000	12.753364000	20.215810000
1	0.283162000	10.270645000	23.595204000
1	10.302542000	11.541961000	22.615315000
1	11.428114000	10.260049000	23.121444000
1	11.420453000	11.808748000	23.991573000
1	10.893459000	10.716451000	26.286354000
1	11.177734000	9.172271000	25.428120000
1	9.677735000	9.410929000	26.365291000
1	8.376193000	14.881224000	25.468653000
1	4.954444000	11.371207000	27.828644000
1	3.324593000	11.756649000	27.217313000
1	4.535322000	13.054946000	27.421334000
1	4.057662000	13.717815000	24.967963000
1	2.914178000	12.349093000	24.841118000
1	4.250610000	12.503743000	23.674665000
1	11.727817000	4.039398000	24.634168000
1	10.642023000	11.880117000	19.275435000
1	12.171785000	8.082133000	21.125957000
1	11.724050000	6.607300000	22.031416000
1	11.390272000	8.214031000	22.723601000
1	9.126940000	6.647192000	19.337989000
1	10.478414000	5.748052000	20.074424000
1	10.800493000	7.212645000	19.102608000
14	6.507909000	7.457034000	20.766157000
1	0.465884000	8.171624000	24.965778000
6	4.609320000	7.389374000	20.843755000
1	4.275910000	6.828573000	19.948694000
1	4.247782000	6.854193000	21.736062000
1	4.124577000	8.379621000	20.821937000
6	6.863361000	8.023895000	18.973571000
1	7.909205000	8.316552000	18.788593000
1	6.592714000	7.210772000	18.273885000
1	6.233123000	8.898382000	18.730469000
6	6.591095000	11.465599000	21.612221000
1	6.313961000	10.892151000	22.518508000
6	6.696969000	12.944874000	22.006714000
6	5.476363000	11.271896000	20.567064000
1	5.748111000	11.775064000	19.619687000
1	5.306353000	10.205883000	20.347970000
1	4.520327000	11.704329000	20.918459000
1	7.530422000	13.120923000	22.706591000
1	5.770233000	13.278588000	22.503105000
1	6.839947000	13.594114000	21.121784000

Intramolcular cyclisation reactions(all PBE functional geometries)

[Fe(NPh)L_2]$^-$:

HAT transition state

[Fe(NPh)L_2]$^-$: Sextet (*-1441.02 cm^{-1}*)

26	0.038316000	-0.223287000	-0.121054000
7	-0.908002000	0.065602000	-1.789489000
7	1.816336000	-0.568965000	-0.306476000
7	-0.678622000	0.170769000	1.609650000
14	-0.053852000	0.996353000	-3.008902000
6	-1.795170000	-1.012568000	-1.942776000
6	3.155417000	-0.726554000	-0.251779000
14	0.503993000	0.192290000	2.930983000
6	-2.007464000	0.564056000	1.857387000
6	-1.172992000	1.707560000	-4.380171000
6	1.314773000	0.046291000	-3.928686000
6	0.798991000	2.434588000	-2.115501000
6	-1.360196000	-2.340835000	-1.590213000
6	-3.162031000	-0.819393000	-2.329160000
6	4.030598000	0.410832000	-0.341906000
6	3.799406000	-2.000239000	-0.082031000
6	1.893036000	1.448603000	2.623252000
6	-0.365377000	0.680854000	4.554679000
6	1.309372000	-1.513403000	3.128262000
6	-2.396260000	1.940414000	1.785402000
6	-2.244383000	-3.420669000	-1.777375000
6	0.044466000	-2.628672000	-1.075054000
6	-4.002142000	-1.934955000	-2.499271000
6	-3.754357000	0.580128000	-2.417503000
6	5.418860000	0.277879000	-0.265901000
6	5.190737000	-2.116277000	-0.014182000
6	-4.289278000	0.018667000	2.579882000
6	-3.695024000	2.318858000	2.168589000
6	-1.436661000	2.999829000	1.261621000
6	-3.549359000	-3.237176000	-2.254444000
6	0.095138000	-3.650477000	0.059480000
6	0.934148000	-3.081690000	-2.242245000
6	-4.596954000	0.871699000	-1.161104000
6	-4.569797000	0.826825000	-3.695713000
6	6.023251000	-0.984313000	-0.102440000
6	-2.678948000	-1.889719000	2.157576000
6	-3.002528000	-0.405564000	2.206898000
6	-4.639977000	1.374759000	2.588773000
6	-2.015820000	3.691239000	0.015121000
6	-1.049124000	4.026949000	2.338339000
6	-2.509921000	-2.498884000	3.560004000
6	-3.711698000	-2.675879000	1.335860000
1	-1.747111000	0.907771000	-4.881642000
1	-0.538258000	2.196281000	-5.143455000
1	-1.889994000	2.456428000	-4.000252000
1	2.051238000	-0.359351000	-3.213359000
1	1.840678000	0.736806000	-4.615565000
1	0.909512000	-0.785686000	-4.532456000
1	0.073185000	3.126871000	-1.657793000
1	1.428619000	3.005291000	-2.823303000
1	1.451775000	2.033532000	-1.319105000
1	2.471472000	1.166411000	1.726838000
1	2.586839000	1.473265000	3.484839000
1	1.492626000	2.467221000	2.474377000
1	-0.776814000	1.704016000	4.490706000
1	0.358461000	0.652596000	5.390600000
1	-1.205366000	0.007905000	4.799857000
1	0.562260000	-2.295774000	3.350341000
1	2.058401000	-1.503226000	3.942075000
1	1.826794000	-1.781342000	2.190351000
1	-1.898061000	-4.435100000	-1.538639000
1	1.074827000	-1.604523000	-0.649700000
1	-5.045706000	-1.774841000	-2.804250000
1	-2.903580000	1.282301000	-2.406127000
1	6.045799000	1.177819000	-0.339865000
1	5.636201000	-3.112452000	0.118130000
1	-5.036778000	-0.735333000	2.862196000
1	-3.976082000	3.380650000	2.126567000
1	-0.518449000	2.466090000	0.952025000
1	-4.217598000	-4.096754000	-2.395800000
1	-0.604899000	-3.402127000	0.875585000
1	1.109748000	-3.693784000	0.500206000
1	-0.142747000	-4.683501000	-0.281152000
1	0.571817000	-4.056115000	-2.640201000
1	1.990897000	-3.214367000	-1.940956000
1	0.911241000	-2.359842000	-3.072300000
1	-5.462786000	0.185293000	-1.098252000
1	-4.983347000	1.908849000	-1.170796000
1	-4.004696000	0.743035000	-0.240226000
1	-3.981106000	0.610027000	-4.604220000
1	-4.904045000	1.880317000	-3.748511000
1	-5.478164000	0.195194000	-3.727131000
1	-1.705908000	-1.960041000	1.638560000
1	-5.646919000	1.689267000	2.891729000
1	-2.233284000	2.948852000	-0.772288000
1	-1.303467000	4.432448000	-0.393893000
1	-2.956620000	4.225877000	0.246184000
1	-1.932707000	4.596050000	2.685988000
1	-0.314020000	4.754986000	1.945830000
1	-0.602539000	3.536151000	3.220659000
1	-3.452560000	-2.429156000	4.136476000
1	-1.725589000	-1.979923000	4.136785000
1	-2.231838000	-3.568071000	3.493705000
1	-3.851325000	-2.232379000	0.335969000
1	-4.696511000	-2.707503000	1.841060000
1	-3.378967000	-3.720008000	1.189974000
1	3.573269000	1.397962000	-0.484085000
1	3.170585000	-2.894972000	0.008169000
1	7.114319000	-1.082712000	-0.045979000

HAT transition state

$[Fe(NPh)L_2]^{-}$: quartet (*-1378.91 cm^{-1}*)

26	0.081069000	-0.214054000	0.036703000
7	-0.680498000	-0.196942000	-1.728018000
7	1.297942000	-1.559566000	0.157929000
7	-0.625590000	0.786778000	1.499733000
14	-0.097127000	1.000522000	-2.873934000
6	-1.518962000	-1.293814000	-1.959855000
6	2.639640000	-1.768943000	0.325665000
14	0.484927000	1.142170000	2.830096000
6	-2.008491000	0.852304000	1.764515000
6	-0.941916000	2.707478000	-2.809888000
6	-0.280718000	0.331391000	-4.649916000
6	1.726327000	1.413788000	-2.506205000
6	-1.007308000	-2.661985000	-1.972978000
6	-2.937872000	-1.089747000	-2.105646000
6	3.555219000	-0.675077000	0.449462000
6	3.203877000	-3.080557000	0.420627000
6	1.695491000	2.520638000	2.320209000
6	-0.502459000	1.693571000	4.366990000
6	1.527438000	-0.339297000	3.401414000
6	-2.727744000	2.083197000	1.612206000
6	-1.957207000	-3.719397000	-2.056791000
6	0.428672000	-3.015783000	-1.888247000
6	-3.804348000	-2.180533000	-2.252893000
6	-3.534137000	0.311035000	-2.052093000
6	4.920295000	-0.879894000	0.668356000
6	4.575366000	-3.276144000	0.614226000
6	-4.055422000	-0.162577000	2.664564000
6	-4.061113000	2.169782000	2.050943000
6	-2.070452000	3.297973000	0.967544000
6	-3.322167000	-3.498155000	-2.218770000
6	0.755448000	-4.494493000	-1.738457000
6	1.391121000	-2.348616000	-2.859943000
6	-4.690439000	0.418401000	-1.045483000
6	-3.985125000	0.786705000	-3.445305000
6	5.451007000	-2.182010000	0.745684000
6	-2.044963000	-1.657742000	2.354486000
6	-2.716755000	-0.295052000	2.255075000
6	-4.727382000	1.064468000	2.593981000
6	-2.954443000	3.894086000	-0.142673000
6	-1.703935000	4.393811000	1.984757000
6	-1.934311000	-2.142332000	3.810497000
6	-2.751109000	-2.700601000	1.472040000
1	-2.005759000	2.693306000	-3.098559000
1	-0.409162000	3.389884000	-3.499908000
1	-0.864856000	3.130474000	-1.793602000
1	0.516492000	-0.390317000	-4.897410000
1	-0.242806000	1.158358000	-5.382889000
1	-1.246612000	-0.192483000	-4.764933000
1	1.799752000	1.905731000	-1.518204000
1	2.117302000	2.117075000	-3.266889000
1	2.377211000	0.523442000	-2.481016000
1	2.258845000	2.204934000	1.423364000
1	2.424829000	2.734024000	3.124372000
1	1.164137000	3.456098000	2.070671000
1	-1.190385000	2.532693000	4.171159000
1	0.199031000	1.994258000	5.167809000
1	-1.113511000	0.854250000	4.745324000
1	0.897996000	-1.232998000	3.553426000
1	2.017353000	-0.091769000	4.362894000
1	2.312843000	-0.601117000	2.673836000
1	-1.597836000	-4.753197000	-2.039146000
1	0.827880000	-2.398258000	-0.660837000
1	-4.882851000	-2.001025000	-2.358643000
1	-2.725735000	0.975321000	-1.697588000
1	5.585360000	-0.010541000	0.764389000
1	4.967361000	-4.300588000	0.682699000
1	-4.581458000	-1.045200000	3.054715000
1	-4.593924000	3.126358000	1.958129000
1	-1.130814000	2.924711000	0.515014000
1	-4.011578000	-4.347004000	-2.318322000
1	0.244233000	-4.960710000	-0.875430000
1	1.842867000	-4.623653000	-1.594087000
1	0.485869000	-5.084615000	-2.643296000
1	1.279449000	-2.772025000	-3.882861000
1	2.440072000	-2.508509000	-2.546280000
1	1.224402000	-1.267427000	-2.917794000
1	-5.563119000	-0.185223000	-1.360143000
1	-5.031915000	1.465890000	-0.952225000
1	-4.384399000	0.079104000	-0.042571000
1	-3.158483000	0.760578000	-4.176135000
1	-4.373887000	1.822866000	-3.405713000
1	-4.793871000	0.139565000	-3.835651000
1	-1.018135000	-1.541023000	1.957564000
1	-5.766910000	1.152239000	2.935299000
1	-3.258243000	3.122682000	-0.869304000
1	-2.416294000	4.687766000	-0.693498000
1	-3.874497000	4.347538000	0.272613000
1	-2.607103000	4.761020000	2.509511000
1	-1.234131000	5.259023000	1.478732000
1	-0.999033000	4.028706000	2.749281000
1	-2.933728000	-2.309120000	4.257075000
1	-1.405131000	-1.408111000	4.443608000
1	-1.379841000	-3.098271000	3.862613000
1	-2.796385000	-2.371715000	0.420707000
1	-3.786978000	-2.890477000	1.814018000
1	-2.206239000	-3.662633000	1.495756000
1	3.143743000	0.337677000	0.356836000
1	2.525666000	-3.939599000	0.367081000
1	6.524699000	-2.340549000	0.906695000

$[Fe(NPh)L_2]^-$: $[Fe^{II}]$-organo radical, sextet

26	-0.041895000	-0.342206000	0.015850000
7	-0.591490000	-0.228975000	-1.844995000
7	1.079184000	-1.867949000	0.536018000
7	-0.663009000	0.758690000	1.495695000
14	-0.028392000	1.039404000	-2.909280000
6	-1.482205000	-1.283082000	-2.134436000
6	2.420996000	-2.085991000	0.554638000
14	0.502351000	1.295856000	2.706850000
6	-2.033842000	0.821761000	1.812732000
6	-0.857816000	2.742667000	-2.693166000
6	-0.248680000	0.526123000	-4.735343000
6	1.807013000	1.410036000	-2.556762000
6	-0.990673000	-2.655066000	-2.254936000
6	-2.894640000	-1.062167000	-2.194978000
6	3.341215000	-1.056540000	0.169528000
6	3.001781000	-3.334442000	0.949728000
6	1.538757000	2.753214000	2.045641000
6	-0.416589000	1.774560000	4.309741000
6	1.749504000	-0.031906000	3.242966000
6	-2.784996000	2.034619000	1.657426000
6	-1.951367000	-3.706595000	-2.361972000
6	0.404063000	-3.023076000	-2.250157000
6	-3.784584000	-2.141622000	-2.369396000
6	-3.477414000	0.333267000	-2.020319000
6	4.722264000	-1.268372000	0.177060000
6	4.386037000	-3.532394000	0.951401000
6	-4.054825000	-0.232464000	2.739230000
6	-4.121762000	2.088835000	2.091692000
6	-2.159691000	3.269759000	1.019479000
6	-3.321253000	-3.462206000	-2.437949000
6	0.820055000	-4.404017000	-1.823200000
6	1.514102000	-2.200952000	-2.824807000
6	-4.622521000	0.372017000	-0.996115000
6	-3.940933000	0.917756000	-3.367907000
6	5.270068000	-2.506984000	0.564661000
6	-1.980159000	-1.657171000	2.489878000
6	-2.709641000	-0.328565000	2.344908000
6	-4.764576000	0.971436000	2.637568000
6	-3.034750000	3.833293000	-0.114367000
6	-1.862975000	4.381152000	2.043624000
6	-2.047727000	-2.219670000	3.918998000
6	-2.477696000	-2.680736000	1.452837000
1	-1.919799000	2.761571000	-2.988628000
1	-0.317853000	3.487940000	-3.308418000
1	-0.788318000	3.061796000	-1.638785000
1	0.524837000	-0.191850000	-5.060739000
1	-0.195151000	1.410568000	-5.396779000
1	-1.230919000	0.041915000	-4.883708000
1	1.919230000	1.784098000	-1.521489000
1	2.180079000	2.196817000	-3.239986000
1	2.447768000	0.518720000	-2.662237000
1	2.057506000	2.433795000	1.122400000
1	2.310134000	3.059315000	2.777430000
1	0.922692000	3.635186000	1.798703000
1	-1.307817000	2.401453000	4.141419000
1	0.265336000	2.304455000	5.000874000
1	-0.760082000	0.851604000	4.812625000
1	1.239604000	-0.974008000	3.511411000
1	2.292031000	0.335020000	4.135982000
1	2.493944000	-0.266369000	2.463701000
1	-1.591273000	-4.738969000	-2.444860000
1	0.555933000	-2.708754000	0.795714000
1	-4.863601000	-1.943912000	-2.414169000
1	-2.656169000	0.960937000	-1.628044000
1	5.386353000	-0.448076000	-0.128859000
1	4.784323000	-4.508357000	1.263122000
1	-4.555806000	-1.121640000	3.147025000
1	-4.678343000	3.030708000	1.987114000
1	-1.198143000	2.930497000	0.587462000
1	-4.026544000	-4.295693000	-2.554635000
1	0.140327000	-4.841325000	-1.069811000
1	1.837275000	-4.374444000	-1.387747000
1	0.866628000	-5.119426000	-2.677226000
1	2.121563000	-2.826835000	-3.514415000
1	2.213308000	-1.841408000	-2.042616000
1	1.151190000	-1.331946000	-3.384785000
1	-5.506529000	-0.191559000	-1.350570000
1	-4.949144000	1.411498000	-0.812771000
1	-4.313312000	-0.051627000	-0.026357000
1	-3.125909000	0.929829000	-4.111983000
1	-4.312203000	1.954183000	-3.248271000
1	-4.765549000	0.311398000	-3.789591000
1	-0.915918000	-1.450099000	2.271338000
1	-5.810313000	1.033204000	2.965069000
1	-3.287185000	3.054487000	-0.852239000
1	-2.511762000	4.648604000	-0.647746000
1	-3.983322000	4.248623000	0.276038000
1	-2.796065000	4.719097000	2.534490000
1	-1.406369000	5.260286000	1.549593000
1	-1.173873000	4.042544000	2.834173000
1	-3.081354000	-2.487789000	4.210564000
1	-1.672562000	-1.484994000	4.654289000
1	-1.431853000	-3.134699000	4.005599000
1	-2.369958000	-2.299689000	0.421292000
1	-3.547163000	-2.920159000	1.607181000
1	-1.908699000	-3.627859000	1.522972000
1	2.927429000	-0.087926000	-0.141566000
1	2.329980000	-4.146415000	1.262541000
1	6.354960000	-2.667649000	0.567705000

[Fe(NPh)L$_2$]$^-$: [Fe^{II}]-organo radical, quartet

26	-0.120088000	-0.332466000	0.026928000
7	-0.533515000	-0.208692000	-1.858003000
7	0.906185000	-1.939519000	0.431618000
7	-0.708955000	0.811748000	1.476351000
14	0.016816000	1.095200000	-2.879560000
6	-1.380022000	-1.299426000	-2.104908000
6	2.239782000	-2.207273000	0.481061000
14	0.484492000	1.339893000	2.660681000
6	-2.074666000	0.867233000	1.818004000
6	-0.855492000	2.769780000	-2.625074000
6	-0.171307000	0.623597000	-4.723101000
6	1.840781000	1.492378000	-2.500908000
6	-0.845355000	-2.662216000	-2.257427000
6	-2.809336000	-1.127599000	-2.081619000
6	3.201653000	-1.198722000	0.150616000
6	2.763887000	-3.487306000	0.846784000
6	1.522083000	2.783876000	1.974419000
6	-0.395701000	1.841832000	4.278093000
6	1.717174000	-0.011889000	3.171671000
6	-2.837067000	2.071238000	1.648376000
6	-1.790370000	-3.743405000	-2.281673000
6	0.541721000	-2.993351000	-2.369854000
6	-3.669206000	-2.230776000	-2.184337000
6	-3.417184000	0.260657000	-1.931183000
6	4.575035000	-1.456265000	0.193699000
6	4.140116000	-3.732490000	0.881913000
6	-4.074216000	-0.179914000	2.796922000
6	-4.168912000	2.125042000	2.097323000
6	-2.224600000	3.298322000	0.984136000
6	-3.162374000	-3.541854000	-2.266094000
6	1.026500000	-4.394098000	-2.102522000
6	1.603378000	-2.081699000	-2.899963000
6	-4.605797000	0.301740000	-0.959736000
6	-3.825859000	0.825429000	-3.305061000
6	5.067686000	-2.723911000	0.557287000
6	-1.993319000	-1.594865000	2.559370000
6	-2.733276000	-0.275064000	2.387705000
6	-4.796021000	1.014923000	2.675367000
6	-3.115707000	3.849275000	-0.143038000
6	-1.908853000	4.420043000	1.991038000
6	-2.020990000	-2.104725000	4.009610000
6	-2.511827000	-2.659251000	1.575394000
1	-1.918695000	2.761326000	-2.915778000
1	-0.340131000	3.541006000	-3.229342000
1	-0.790177000	3.071614000	-1.565554000
1	0.710079000	0.074590000	-5.099854000
1	-0.301075000	1.527922000	-5.346000000
1	-1.053256000	-0.026044000	-4.869390000
1	1.939860000	1.829906000	-1.452053000
1	2.202615000	2.309802000	-3.153429000
1	2.495999000	0.615808000	-2.636107000
1	2.019240000	2.458398000	1.041619000
1	2.309257000	3.089515000	2.689385000
1	0.904617000	3.667515000	1.736165000
1	-1.283761000	2.475629000	4.118043000
1	0.303703000	2.373682000	4.949909000
1	-0.739277000	0.928837000	4.798472000
1	1.191073000	-0.938399000	3.462942000
1	2.298476000	0.343860000	4.044560000
1	2.430865000	-0.274036000	2.372412000
1	-1.410753000	-4.766688000	-2.381001000
1	0.343611000	-2.776080000	0.610086000
1	-4.754899000	-2.071632000	-2.168735000
1	-2.620067000	0.903156000	-1.512314000
1	5.275193000	-0.650693000	-0.067069000
1	4.497180000	-4.730995000	1.170806000
1	-4.562246000	-1.062980000	3.232856000
1	-4.733477000	3.060644000	1.980122000
1	-1.269821000	2.953041000	0.542279000
1	-3.846888000	-4.398519000	-2.326607000
1	0.408433000	-4.935028000	-1.364098000
1	2.059440000	-4.362655000	-1.705510000
1	1.062196000	-5.019976000	-3.024091000
1	2.287522000	-2.652736000	-3.562034000
1	2.239471000	-1.659523000	-2.094120000
1	1.190903000	-1.244432000	-3.475624000
1	-5.475392000	-0.261094000	-1.349388000
1	-4.938403000	1.342402000	-0.795842000
1	-4.339583000	-0.116097000	0.025312000
1	-2.979593000	0.831279000	-4.013063000
1	-4.207842000	1.861205000	-3.216223000
1	-4.627377000	0.206015000	-3.751323000
1	-0.935947000	-1.395649000	2.303592000
1	-5.838126000	1.075565000	3.014506000
1	-3.374336000	3.063844000	-0.872235000
1	-2.600894000	4.661070000	-0.689513000
1	-4.060646000	4.265720000	0.254947000
1	-2.832138000	4.764252000	2.495931000
1	-1.460111000	5.293340000	1.479691000
1	-1.205433000	4.088099000	2.772017000
1	-3.045579000	-2.365223000	4.337863000
1	-1.630356000	-1.342381000	4.707665000
1	-1.398691000	-3.013582000	4.113339000
1	-2.422903000	-2.322596000	0.526678000
1	-3.578635000	-2.890445000	1.758471000
1	-1.942930000	-3.603522000	1.676592000
1	2.825596000	-0.208387000	-0.139878000
1	2.055577000	-4.285487000	1.109623000
1	6.146116000	-2.921621000	0.586856000

Transition state for C–N bond formation

$[Fe(NPh)L_2]^-$: Quartet (*-377.61 cm^{-1}*)

26	-0.079818000	-0.227328000	-0.107142000
7	-0.603045000	-0.071043000	-1.973416000
7	0.768858000	-2.052747000	-0.247539000
7	-0.541071000	0.782825000	1.473563000
14	-0.142474000	1.215323000	-3.047358000
6	-1.408020000	-1.192243000	-2.171929000
6	2.034363000	-2.231133000	0.322331000
14	0.675195000	1.291144000	2.630098000
6	-1.899717000	0.792417000	1.834918000
6	-1.085144000	2.866138000	-2.887325000
6	-0.282097000	0.623597000	-4.854159000
6	1.664452000	1.711034000	-2.681022000
6	-0.855964000	-2.546889000	-2.228155000
6	-2.846643000	-1.063176000	-2.255902000
6	3.116137000	-1.383648000	-0.048718000
6	2.296499000	-3.260451000	1.268649000
6	1.703059000	2.744307000	1.944671000
6	-0.153875000	1.778234000	4.280420000
6	1.922221000	-0.069921000	3.083876000
6	-2.690308000	1.987436000	1.720803000
6	-1.742455000	-3.642730000	-2.390827000
6	0.573937000	-2.816118000	-2.006475000
6	-3.658397000	-2.183830000	-2.457342000
6	-3.508128000	0.296548000	-2.081635000
6	4.386966000	-1.560213000	0.506558000
6	3.567977000	-3.417147000	1.831999000
6	-3.871828000	-0.332904000	2.787686000
6	-4.015680000	1.998260000	2.190907000
6	-2.117954000	3.239302000	1.065774000
6	-3.117532000	-3.481581000	-2.538968000
6	0.992254000	-4.260021000	-1.794972000
6	1.586258000	-2.076379000	-2.853872000
6	-4.576671000	0.281691000	-0.975984000
6	-4.091005000	0.821631000	-3.405282000
6	4.630822000	-2.576601000	1.453069000
6	-1.777299000	-1.703530000	2.434968000
6	-2.537982000	-0.387096000	2.351916000
6	-4.614786000	0.855447000	2.735037000
6	-3.053765000	3.794985000	-0.022696000
6	-1.786538000	4.348663000	2.081012000
6	-1.659107000	-2.222524000	3.877287000
6	-2.384613000	-2.761764000	1.496533000
1	-2.155597000	2.791330000	-3.141052000
1	-0.624869000	3.617736000	-3.557223000
1	-1.004181000	3.241329000	-1.852141000
1	0.417663000	-0.208589000	-5.048181000
1	-0.058891000	1.441847000	-5.563768000
1	-1.302062000	0.252877000	-5.062802000
1	1.762975000	1.960607000	-1.607420000
1	1.961703000	2.599194000	-3.270527000
1	2.376530000	0.896046000	-2.901216000
1	2.176384000	2.432569000	0.994778000
1	2.506540000	3.043089000	2.644604000
1	1.076523000	3.628772000	1.733027000
1	-1.062526000	2.388199000	4.144481000
1	0.554797000	2.335290000	4.921560000
1	-0.455487000	0.863323000	4.822776000
1	1.408895000	-1.002333000	3.380039000
1	2.540469000	0.265931000	3.939263000
1	2.602004000	-0.315948000	2.251149000
1	-1.327003000	-4.655929000	-2.442880000
1	0.107154000	-2.699058000	0.199445000
1	-4.746761000	-2.048464000	-2.530529000
1	-2.706653000	0.984694000	-1.756386000
1	5.198594000	-0.882800000	0.209682000
1	3.732512000	-4.208288000	2.575819000
1	-4.339506000	-1.243825000	3.188292000
1	-4.599662000	2.926401000	2.114953000
1	-1.172080000	2.917280000	0.587270000
1	-3.766440000	-4.350993000	-2.705913000
1	0.341399000	-4.794118000	-1.079499000
1	2.025860000	-4.299216000	-1.406051000
1	0.979117000	-4.828756000	-2.747964000
1	1.528092000	-2.448420000	-3.897258000
1	2.616470000	-2.249198000	-2.495188000
1	1.378856000	-0.999910000	-2.859096000
1	-5.422221000	-0.384557000	-1.233104000
1	-4.989431000	1.294365000	-0.812701000
1	-4.153820000	-0.058331000	-0.015825000
1	-3.323854000	0.861659000	-4.198700000
1	-4.510169000	1.839417000	-3.282693000
1	-4.905922000	0.164388000	-3.765336000
1	-0.751743000	-1.481199000	2.079230000
1	-5.650971000	0.884372000	3.095778000
1	-3.312188000	3.020042000	-0.763067000
1	-2.575715000	4.633828000	-0.562179000
1	-3.998015000	4.177096000	0.410067000
1	-2.696693000	4.665636000	2.626403000
1	-1.373798000	5.240295000	1.570429000
1	-1.048159000	4.017408000	2.829587000
1	-2.649962000	-2.470289000	4.304712000
1	-1.193151000	-1.466101000	4.534046000
1	-1.039217000	-3.138791000	3.918410000
1	-2.429293000	-2.400056000	0.453228000
1	-3.416484000	-3.024299000	1.798889000
1	-1.791288000	-3.697533000	1.510508000
1	2.918892000	-0.562981000	-0.748569000
1	1.473194000	-3.922784000	1.570095000
1	5.626966000	-2.702247000	1.894263000

[Fe(NPh)L$_2$]:

HAT transition state

[Fe(NPh)L$_2$]: quintet (-1232.35 cm^{-1})

26	0.007517000	-0.212147000	-0.137664000
7	-0.732543000	-1.961927000	-0.027610000
7	1.212212000	-0.094078000	1.205304000
7	-0.680795000	1.374640000	-0.890564000
14	-0.388853000	-3.094472000	-1.360876000
6	-1.432817000	-2.225466000	1.156795000
6	2.162565000	0.692528000	1.829787000
14	0.622243000	2.484804000	-1.402467000
6	-2.016253000	1.816863000	-0.717677000
6	-1.448809000	-2.907682000	-2.920274000
6	-0.569552000	-4.872322000	-0.728164000
6	1.381654000	-2.719543000	-1.947650000
6	-0.746729000	-2.322379000	2.438583000
6	-2.870350000	-2.309603000	1.136606000
6	3.449747000	0.881583000	1.254886000
6	1.877659000	1.353714000	3.058218000
6	1.995565000	1.358189000	-2.077548000
6	0.069184000	3.653548000	-2.783890000
6	1.266764000	3.591658000	-0.008312000
6	-2.930646000	1.865332000	-1.820400000
6	-1.534356000	-2.446345000	3.616733000
6	0.720215000	-2.249022000	2.582212000
6	-3.574884000	-2.502160000	2.329448000
6	-3.654998000	-2.112635000	-0.152568000
6	4.385830000	1.730893000	1.855086000
6	2.825575000	2.187578000	3.660405000
6	-3.780526000	2.736699000	0.719295000
6	-4.228240000	2.367805000	-1.619714000
6	-2.569971000	1.333707000	-3.201456000
6	-2.917397000	-2.572759000	3.570019000
6	1.289273000	-2.004751000	3.968387000
6	1.605313000	-3.178221000	1.768989000
6	-4.685047000	-0.976675000	-0.024490000
6	-4.325756000	-3.416158000	-0.620395000
6	4.080893000	2.390135000	3.058983000
6	-1.577576000	2.198741000	1.807236000
6	-2.468051000	2.252997000	0.573261000
6	-4.661067000	2.808698000	-0.364700000
6	-3.368341000	0.050807000	-3.502489000
6	-2.807970000	2.359791000	-4.324753000
6	-1.440955000	3.567856000	2.495819000
6	-2.079577000	1.134634000	2.800139000
1	-2.514617000	-3.145456000	-2.769519000
1	-1.052331000	-3.601955000	-3.685211000
1	-1.374357000	-1.885270000	-3.327018000
1	0.217157000	-5.153642000	-0.008475000
1	-0.527741000	-5.577003000	-1.578396000
1	-1.544093000	-5.000997000	-0.224239000
1	1.446234000	-1.701349000	-2.377509000
1	1.667642000	-3.423667000	-2.751008000
1	2.128671000	-2.793821000	-1.139874000
1	2.267925000	0.545871000	-1.378104000
1	2.916517000	1.940234000	-2.264561000
1	1.677760000	0.900492000	-3.031825000
1	-0.104141000	3.127913000	-3.737371000
1	0.859727000	4.409302000	-2.946838000
1	-0.856041000	4.188183000	-2.504327000

1	0.501192000	4.338209000	0.267644000
1	2.156842000	4.142607000	-0.365445000
1	1.552892000	3.036362000	0.898804000
1	-1.030687000	-2.497230000	4.587195000
1	0.940174000	-1.070472000	1.924323000
1	-4.670542000	-2.561556000	2.298223000
1	-2.925235000	-1.804569000	-0.921438000
1	5.367714000	1.872846000	1.386108000
1	2.580762000	2.693732000	4.602710000
1	-4.116281000	3.068625000	1.710408000
1	-4.920961000	2.405376000	-2.470495000
1	-1.494917000	1.073050000	-3.174971000
1	-3.490416000	-2.719300000	4.493721000
1	0.754532000	-1.210075000	4.515973000
1	2.348796000	-1.700043000	3.890506000
1	1.266273000	-2.922517000	4.592775000
1	1.733259000	-4.139836000	2.308541000
1	2.612577000	-2.745112000	1.626564000
1	1.187350000	-3.395264000	0.780725000
1	-5.479483000	-1.227533000	0.702653000
1	-5.174227000	-0.785268000	-0.996126000
1	-4.211784000	-0.035227000	0.301237000
1	-3.592279000	-4.229266000	-0.760961000
1	-4.854511000	-3.262976000	-1.579639000
1	-5.071118000	-3.766565000	0.117846000
1	-0.572037000	1.890580000	1.471377000
1	-5.678753000	3.195437000	-0.231097000
1	-3.240955000	-0.700822000	-2.708180000
1	-3.050968000	-0.400455000	-4.460570000
1	-4.448760000	0.274323000	-3.578938000
1	-3.887058000	2.561114000	-4.458440000
1	-2.428355000	1.974357000	-5.288997000
1	-2.315107000	3.323794000	-4.122052000
1	-2.394114000	3.891281000	2.953882000
1	-1.129888000	4.352250000	1.784582000
1	-0.684085000	3.521039000	3.300033000
1	-2.149776000	0.138244000	2.333687000
1	-3.083619000	1.390295000	3.186834000
1	-1.400442000	1.050132000	3.669016000
1	3.690184000	0.350017000	0.327108000
1	0.890114000	1.209951000	3.511140000
1	4.817949000	3.052384000	3.528528000

[Fe(NPh)L$_2$]: triplet (-608.83 cm^{-1})

26	0.004490000	-0.164012000	0.121586000
7	-0.535529000	-1.913583000	-0.072908000
7	1.154919000	-0.263101000	1.447462000
7	-0.568870000	1.316004000	-0.801599000
14	-0.240335000	-2.840555000	-1.580326000
6	-1.233990000	-2.380651000	1.061324000
6	1.394671000	0.526182000	2.577761000
14	0.724548000	2.143267000	-1.770159000
6	-1.848230000	1.940433000	-0.701687000
6	-0.656754000	-4.658547000	-1.237569000
6	1.567298000	-2.606967000	-2.107459000
6	-1.290194000	-2.293632000	-3.054408000
6	-0.522471000	-2.773273000	2.261608000
6	-2.668692000	-2.385075000	1.063930000
6	2.550578000	1.343591000	2.642802000
6	0.540649000	0.473317000	3.710456000
6	1.067764000	1.279090000	-3.425290000
6	0.231711000	3.952796000	-2.031748000
6	2.358822000	2.102929000	-0.819829000
6	-2.729683000	1.997634000	-1.828296000
6	-1.280009000	-3.110905000	3.415465000
6	0.943896000	-2.732493000	2.343234000
6	-3.354269000	-2.801225000	2.212430000
6	-3.466974000	-1.844688000	-0.114587000
6	2.827190000	2.099028000	3.789182000
6	0.833624000	1.218096000	4.859237000
6	-3.474701000	3.264509000	0.565401000
6	-3.931339000	2.723314000	-1.731392000
6	-2.454936000	1.269712000	-3.138331000
6	-2.670381000	-3.164179000	3.385599000
6	1.594886000	-2.739700000	3.712070000
6	1.803315000	-3.437355000	1.309384000
6	-4.311555000	-0.627263000	0.306705000
6	-4.339852000	-2.924598000	-0.776801000
6	1.972043000	2.041883000	4.903129000
6	-1.424748000	2.450784000	1.791123000
6	-2.264313000	2.551152000	0.527660000
6	-4.304804000	3.374585000	-0.554651000
6	-3.575056000	0.261341000	-3.457343000
6	-2.274119000	2.232695000	-4.326545000
6	-0.377555000	3.574565000	1.874011000
6	-2.266659000	2.392276000	3.073961000
1	-0.005263000	-5.122373000	-0.478089000
1	-0.560156000	-5.237316000	-2.174294000
1	-1.698790000	-4.752411000	-0.883894000
1	1.853252000	-1.542894000	-2.025548000
1	1.693076000	-2.915017000	-3.161431000
1	2.273325000	-3.186876000	-1.491189000
1	-2.368186000	-2.462531000	-2.900230000
1	-0.977102000	-2.883029000	-3.936486000
1	-1.133657000	-1.228482000	-3.289480000
1	1.273461000	0.205933000	-3.266060000
1	1.974081000	1.728735000	-3.872555000
1	0.250898000	1.368137000	-4.158414000
1	-0.776345000	4.060341000	-2.465051000
1	0.957804000	4.440086000	-2.707941000
1	0.237613000	4.497224000	-1.071492000
1	2.274123000	2.638304000	0.140887000
1	3.138199000	2.600214000	-1.426923000
1	2.687487000	1.070323000	-0.607584000
1	-0.752494000	-3.380938000	4.336598000
1	1.083348000	-1.387536000	1.844846000
1	-4.452065000	-2.801560000	2.207999000
1	-2.734834000	-1.490358000	-0.860851000
1	3.725090000	2.728913000	3.818270000
1	0.163053000	1.161748000	5.726190000
1	-3.777095000	3.744250000	1.503168000
1	-4.592522000	2.769927000	-2.606466000
1	-1.519652000	0.699463000	-3.000815000
1	-3.229490000	-3.477890000	4.275803000
1	1.084778000	-2.090018000	4.441574000
1	2.640772000	-2.390497000	3.632568000
1	1.635000000	-3.766269000	4.134731000
1	2.233221000	-4.359635000	1.751980000
1	2.652883000	-2.809509000	0.979927000
1	1.235556000	-3.725963000	0.418956000
1	-5.084874000	-0.910092000	1.044556000
1	-4.824556000	-0.180582000	-0.563803000
1	-3.684360000	0.161828000	0.755476000
1	-3.739901000	-3.789278000	-1.111668000
1	-4.866846000	-2.514129000	-1.658153000
1	-5.108995000	-3.305634000	-0.079500000
1	-0.872884000	1.493074000	1.718345000
1	-5.240619000	3.944030000	-0.502117000
1	-3.734824000	-0.439849000	-2.622574000
1	-3.327261000	-0.329400000	-4.357680000
1	-4.533832000	0.776328000	-3.650271000
1	-3.198315000	2.812251000	-4.506343000
1	-2.049492000	1.673239000	-5.253717000
1	-1.459302000	2.956549000	-4.160580000
1	-0.870483000	4.564663000	1.899644000
1	0.300612000	3.558098000	1.004672000
1	0.239419000	3.470119000	2.784368000
1	-3.027573000	1.592817000	3.021703000
1	-2.787611000	3.346206000	3.276551000
1	-1.614524000	2.192609000	3.940767000
1	3.229234000	1.357105000	1.784046000
1	-0.358227000	-0.153869000	3.664211000
1	2.194174000	2.629664000	5.801824000

[Fe(NPh)L$_2$]: [Fe^{II}]-organo radical, quintet

26	-0.254635000	-0.154116000	0.408357000
7	-0.525470000	-1.973352000	-0.072188000
7	0.832786000	0.038144000	1.961010000
7	-0.804219000	1.205179000	-0.812840000
14	-0.339311000	-2.893124000	-1.576121000
6	-1.158891000	-2.239223000	1.130405000
6	1.634550000	0.930982000	2.633616000
14	0.565533000	1.771228000	-1.795285000
6	-2.058104000	1.853199000	-0.671933000
6	-1.522800000	-2.380531000	-2.960902000
6	-0.606396000	-4.729730000	-1.177858000
6	1.391312000	-2.623642000	-2.306208000
6	-0.418677000	-2.755509000	2.320464000
6	-2.601737000	-2.027782000	1.253059000
6	1.860196000	2.260418000	2.171303000
6	2.281160000	0.550046000	3.847951000
6	2.155276000	0.990331000	-1.092574000
6	0.615372000	1.248779000	-3.627737000
6	0.633813000	3.661286000	-1.696081000
6	-3.012512000	1.901262000	-1.743226000
6	-1.175607000	-2.912306000	3.544575000
6	0.961578000	-3.052543000	2.353769000
6	-3.234889000	-2.228387000	2.470832000
6	-3.442168000	-1.636396000	0.044388000
6	2.675296000	3.145261000	2.884609000
6	3.094758000	1.440545000	4.552363000
6	-3.675722000	3.100760000	0.707964000
6	-4.243209000	2.556387000	-1.560446000
6	-2.750439000	1.274584000	-3.103722000
6	-2.523839000	-2.661756000	3.621442000
6	1.648439000	-3.425095000	3.647666000
6	1.864922000	-3.090834000	1.154661000
6	-4.504934000	-0.571285000	0.356052000
6	-4.109872000	-2.893510000	-0.548407000
6	3.301753000	2.749293000	4.079113000
6	-1.501521000	2.485121000	1.779156000
6	-2.425060000	2.473111000	0.574058000
6	-4.591110000	3.154588000	-0.346472000
6	-3.836529000	0.257976000	-3.499072000
6	-2.612160000	2.356392000	-4.191726000
6	-1.191436000	3.910107000	2.265759000
6	-2.028276000	1.602039000	2.924208000
1	-2.574381000	-2.639572000	-2.761774000
1	-1.217811000	-2.888445000	-3.894952000
1	-1.462885000	-1.293066000	-3.133042000
1	0.148999000	-5.127333000	-0.477868000
1	-0.552309000	-5.321810000	-2.109723000
1	-1.599347000	-4.901169000	-0.726009000
1	1.520052000	-1.580925000	-2.645196000
1	1.497609000	-3.269692000	-3.197546000
1	2.214857000	-2.863795000	-1.614564000
1	2.416361000	1.319176000	-0.074778000
1	2.985279000	1.283192000	-1.763616000
1	2.128705000	-0.114634000	-1.098086000
1	0.449215000	0.163079000	-3.746634000
1	1.630396000	1.470559000	-4.008479000
1	-0.102571000	1.780115000	-4.270889000
1	-0.323946000	4.096395000	-2.034873000
1	1.441120000	4.067135000	-2.332006000
1	0.803555000	4.002771000	-0.659485000
1	-0.663838000	-3.281056000	4.437550000
1	0.817529000	-0.858981000	2.468165000
1	-4.313994000	-2.050338000	2.549941000
1	-2.755994000	-1.214876000	-0.714081000
1	2.822761000	4.162337000	2.499628000
1	3.573874000	1.111533000	5.483356000
1	-3.931379000	3.565675000	1.669081000
1	-4.952977000	2.588552000	-2.397403000
1	-1.795139000	0.728448000	-3.025661000
1	-3.057249000	-2.811306000	4.568078000
1	1.239297000	-2.918865000	4.537347000
1	2.723557000	-3.181702000	3.589802000
1	1.584766000	-4.517536000	3.837722000
1	2.854162000	-3.495174000	1.428244000
1	2.016681000	-2.082521000	0.722646000
1	1.453751000	-3.710850000	0.343542000
1	-5.294796000	-0.961600000	1.024057000
1	-4.999493000	-0.241929000	-0.573706000
1	-4.066316000	0.323955000	0.826179000
1	-3.374985000	-3.680153000	-0.787217000
1	-4.665376000	-2.649525000	-1.473156000
1	-4.829648000	-3.321563000	0.173721000
1	-0.546133000	2.046425000	1.449487000
1	-5.560126000	3.653487000	-0.224188000
1	-3.960417000	-0.522167000	-2.729503000
1	-3.575520000	-0.242733000	-4.449288000
1	-4.817526000	0.747318000	-3.641139000
1	-3.568286000	2.893202000	-4.334052000
1	-2.331468000	1.912127000	-5.164873000
1	-1.852477000	3.110824000	-3.922457000
1	-2.091379000	4.411660000	2.667485000
1	-0.801470000	4.535862000	1.442842000
1	-0.431421000	3.884220000	3.066982000
1	-2.210822000	0.563653000	2.592171000
1	-2.980671000	1.993915000	3.328507000
1	-1.295320000	1.560722000	3.750080000
1	1.376259000	2.595330000	1.249083000
1	2.124226000	-0.468657000	4.227277000
1	3.939947000	3.448021000	4.632342000

[Fe(NPh)L$_2$]: [Fe^{II}]-organo radical, triplet

26	-0.099450000	-0.222680000	0.264411000
7	-0.552067000	-1.975824000	0.019367000
7	0.805426000	-0.126408000	1.838066000
7	-0.623365000	1.195562000	-0.783115000
14	-0.165077000	-2.894152000	-1.465526000
6	-1.382278000	-2.257121000	1.109329000
6	1.517734000	0.792033000	2.579614000
14	0.790957000	1.897941000	-1.668783000
6	-1.890271000	1.848802000	-0.695747000
6	-0.561712000	-4.727522000	-1.172085000
6	1.660803000	-2.639736000	-1.900120000
6	-1.116323000	-2.276084000	-2.981171000
6	-0.814801000	-2.667107000	2.410573000
6	-2.814940000	-2.101885000	0.993686000
6	1.825172000	2.098559000	2.110099000
6	1.971360000	0.445726000	3.885916000
6	0.910474000	1.551882000	-3.533609000
6	0.755564000	3.768521000	-1.385568000
6	2.393907000	1.097044000	-1.001095000
6	-2.760698000	1.954842000	-1.829314000
6	-1.721906000	-2.742373000	3.526958000
6	0.552128000	-2.993013000	2.642600000
6	-3.626625000	-2.280077000	2.113996000
6	-3.472742000	-1.712294000	-0.323994000
6	2.539376000	3.001501000	2.903975000
6	2.687662000	1.354439000	4.669621000
6	-3.592110000	3.026622000	0.629517000
6	-4.005073000	2.597699000	-1.695325000
6	-2.416509000	1.397102000	-3.203667000
6	-3.083822000	-2.580779000	3.383318000
6	1.055451000	-3.261314000	4.041897000
6	1.548540000	-3.305353000	1.561390000
6	-4.550942000	-0.630099000	-0.148266000
6	-4.066678000	-2.951156000	-1.022511000
6	2.980042000	2.643560000	4.189684000
6	-1.499557000	2.349396000	1.820086000
6	-2.334143000	2.403888000	0.552752000
6	-4.434741000	3.134505000	-0.480038000
6	-3.437558000	0.351463000	-3.687879000
6	-2.288597000	2.528056000	-4.242403000
6	-1.242673000	3.741153000	2.420933000
6	-2.101138000	1.388730000	2.860520000
1	0.255518000	-5.256650000	-0.651377000
1	-0.730499000	-5.234686000	-2.139304000
1	-1.475426000	-4.842291000	-0.562449000
1	1.864394000	-1.574952000	-2.109039000
1	1.890411000	-3.208562000	-2.820398000
1	2.353698000	-2.970711000	-1.109878000
1	-2.187735000	-2.530292000	-2.962901000
1	-0.673566000	-2.729745000	-3.887500000
1	-1.022799000	-1.180434000	-3.070160000
1	0.809742000	0.471947000	-3.746558000
1	1.931206000	1.851817000	-3.839483000
1	0.197519000	2.105094000	-4.161518000
1	-0.183677000	4.197158000	-1.779110000
1	1.602283000	4.258635000	-1.899377000
1	0.807437000	4.019281000	-0.311341000
1	3.104462000	1.888248000	-0.699677000
1	2.871937000	0.505686000	-1.801958000
1	2.268294000	0.432668000	-0.127257000
1	-1.324504000	-2.987365000	4.515848000
1	0.698206000	-1.027531000	2.349963000
1	-4.709525000	-2.140610000	2.017339000
1	-2.681622000	-1.288848000	-0.969711000
1	2.753170000	4.003226000	2.509712000
1	3.020195000	1.052362000	5.670959000
1	-3.912794000	3.441226000	1.593889000
1	-4.659409000	2.667190000	-2.573957000
1	-1.443411000	0.886518000	-3.118230000
1	-3.744748000	-2.683733000	4.252577000
1	0.602010000	-2.612547000	4.810484000
1	2.149407000	-3.118042000	4.087709000
1	0.862810000	-4.311588000	4.349664000
1	2.365969000	-3.927765000	1.966968000
1	2.009697000	-2.396755000	1.126094000
1	1.083451000	-3.849608000	0.725672000
1	-5.422837000	-1.008820000	0.415876000
1	-4.923940000	-0.293682000	-1.130711000
1	-4.157250000	0.255084000	0.378333000
1	-3.313571000	-3.740522000	-1.185759000
1	-4.498973000	-2.681494000	-2.004355000
1	-4.876013000	-3.387805000	-0.408497000
1	-0.522931000	1.934740000	1.539294000
1	-5.411378000	3.626616000	-0.398070000
1	-3.556332000	-0.466716000	-2.959618000
1	-3.113089000	-0.092013000	-4.646851000
1	-4.433033000	0.804084000	-3.849679000
1	-3.265277000	3.017189000	-4.411872000
1	-1.940279000	2.138126000	-5.216849000
1	-1.586706000	3.312738000	-3.910150000
1	-2.172529000	4.212364000	2.790277000
1	-0.796114000	4.424270000	1.675863000
1	-0.541924000	3.662832000	3.271392000
1	-2.252549000	0.379764000	2.438852000
1	-3.079401000	1.752305000	3.228111000
1	-1.423826000	1.292786000	3.728768000
1	1.484882000	2.401138000	1.116634000
1	1.745052000	-0.555563000	4.271540000
1	3.539830000	3.355805000	4.806734000

Transition state for C–N bond formation

[Fe(NPh)L_2]: no transition state found.

3. Details of X-Ray diffraction analysis, refinement and molecular structures

Crystal structure and refinements of K{crypt.222}[Fe(NTol)L_2], **1**

Empirical formula	$C_{55}H_{95}FeKN_5O_6Si_2$
Formula weight	1073.48
Temperature/K	230.00
Crystal system	monoclinic
Space group	$P2_1/c$
a/Å	18.8435(14)
b/Å	15.5864(11)
c/Å	22.6289(18)
α/°	90
β/°	110.377(2)
γ/°	90
Volume/Å^3	6230.3(8)
Z	4
ρ_{calc}g/cm^3	1.144
μ/mm^{-1}	0.395
F(000)	2324.0
Crystal size/mm^3	0.136 × 0.103 × 0.085
Radiation	MoKα (λ = 0.71073)
2Θ range for data collection/°	4.35 to 51.566
Index ranges	−23 ≤ h ≤ 22, −18 ≤ k ≤ 19, −27 ≤ l ≤ 27
Reflections collected	144646
Independent reflections	11919 [R_{int} = 0.1278, R_{sigma} = 0.1333]
Data/restraints/parameters	11919/134/717
Goodness-of-fit on F^2	1.179
Final R indexes [I>=2σ (I)]	R_1 = 0.0869, wR_2 = 0.1544
Final R indexes [all data]	R_1 = 0.1240, wR_2 = 0.1644
Largest diff. peak/hole / e Å^{-3}	0.77/−0.80

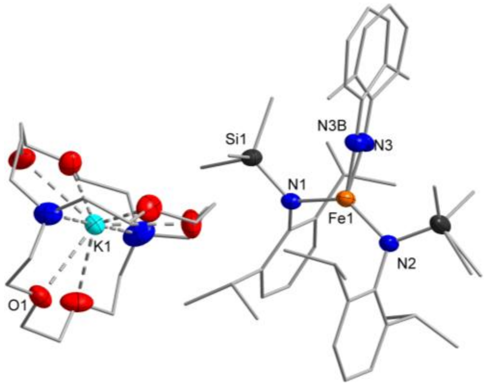

Figure S 51. Molecular structure of K{crypt.222}[Fe(NTol)L_2], **1**, depicting a disorder in the NTol group (0.6:0.4) and in one of the $SiMe_3$ groups (0.5:0.5).

Crystal structure and refinements of K{crypt.222}[Fe(NXyl)L_2], **2**

Empirical formula	$C_{56}H_{97}FeKN_5O_6Si_2$
Formula weight	1087.51
Temperature/K	100.00
Crystal system	triclinic
Space group	P-1
a/Å	12.0866(4)
b/Å	15.7429(6)
c/Å	16.4138(5)
α/°	88.8820(10)
β/°	88.2760(10)
γ/°	88.153(2)
Volume/Å^3	3119.61(18)
Z	2
ρ_{calc}g/cm^3	1.158
μ/mm^{-1}	0.395
F(000)	1178.0
Crystal size/mm^3	0.347 × 0.278 × 0.25
Radiation	MoKα (λ = 0.71073)
2Θ range for data collection/°	4.13 to 67.546
Index ranges	−18 ≤ h ≤ 18, −22 ≤ k ≤ 23, −19 ≤ l ≤ 25
Reflections collected	110726
Independent reflections	21765 [R_{int} = 0.0229, R_{sigma} = 0.0206]
Data/restraints/parameters	21765/0/666
Goodness-of-fit on F^2	1.036
Final R indexes [I>=2σ (I)]	R_1 = 0.0345, wR_2 = 0.0875
Final R indexes [all data]	R_1 = 0.0395, wR_2 = 0.0903
Largest diff. peak/hole / e Å^{-3}	0.87/−0.94

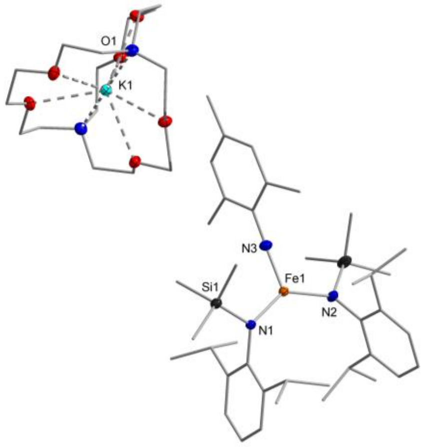

Figure S 52. Molecular structure of K{crypt.222}[Fe(NXyl)L_2], **2**, with a disorder in the *o*-CH_3 of the imido group (0.5:0.5).

Crystal structure and refinements of K{crypt.222}[Fe(NDipp)L_2], **3**

Empirical formula	$C_{60}H_{105}FeKN_5O_6Si_2$
Formula weight	1215.72
Temperature/K	100.00
Crystal system	monoclinic
Space group	$P2_1/c$
a/Å	14.6326(4)
b/Å	20.0038(7)
c/Å	24.2267(8)
α/°	90
β/°	92.0810(10)
γ/°	90
Volume/Å^3	7086.7(4)
Z	4
ρ_{calc}g/cm^3	1.139
μ/mm^{-1}	0.355
F(000)	2644.0
Crystal size/mm^3	0.316 × 0.12 × 0.1
Radiation	MoKα (λ = 0.71073)
2Θ range for data collection/°	3.882 to 58.05
Index ranges	−18 ≤ h ≤ 19, -27 ≤ k ≤ 27, −33 ≤ l ≤ 32
Reflections collected	218784
Independent reflections	18840 [R_{int} = 0.0386, R_{sigma} = 0.0189]
Data/restraints/parameters	18840/91/920
Goodness-of-fit on F^2	1.040
Final R indexes [I>=2σ (I)]	R_1 = 0.0609, wR_2 = 0.1441
Final R indexes [all data]	R_1 = 0.0733, wR_2 = 0.1525
Largest diff. peak/hole / e Å^{-3}	0.85/−0.95

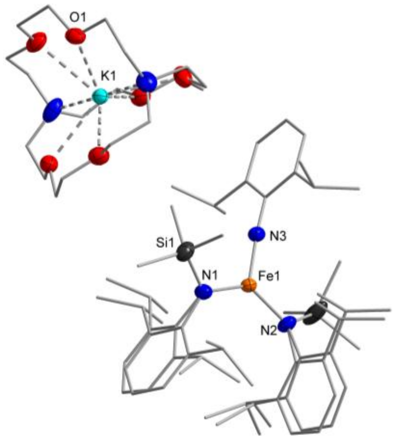

Figure S 53. Molecular structure of K{crypt.222}[Fe(NDipp)L_2], **3**, depicting disorders in both amido Dipp groups (0.51:0.49, left, 0.62:0.38, right)

Crystal structure and refinements of K{crypt.222}[Fe(NTripp)L_2], **4**

Empirical formula	$C_{69}H_{115}F_2FeKN_5O_6Si_2$
Formula weight	1299.78
Temperature/K	100.00
Crystal system	triclinic
Space group	P-1
a/Å	13.0324(4)
b/Å	15.6982(5)
c/Å	19.3384(6)
α/°	107.8460(10)
β/°	97.7920(10)
γ/°	96.7600(10)
Volume/Å^3	3677.6(2)
Z	2
ρ_{calc}g/cm^3	1.174
μ/mm^{-1}	0.349
F(000)	1406.0
Crystal size/mm^3	0.225 × 0.224 × 0.145
Radiation	MoKα (λ = 0.71073)
2Θ range for data collection/°	4.098 to 53.436
Index ranges	−16 ≤ h ≤ 16, −19 ≤ k ≤ 19, −24 ≤ l ≤ 24
Reflections collected	104519
Independent reflections	15541 [R_{int} = 0.0281, R_{sigma} = 0.0173]
Data/restraints/parameters	15541/0/795
Goodness-of-fit on F^2	1.032
Final R indexes [I>=2σ (I)]	R_1 = 0.0363, wR_2 = 0.0901
Final R indexes [all data]	R_1 = 0.0396, wR_2 = 0.0921
Largest diff. peak/hole / e Å^{-3}	0.57/−0.65

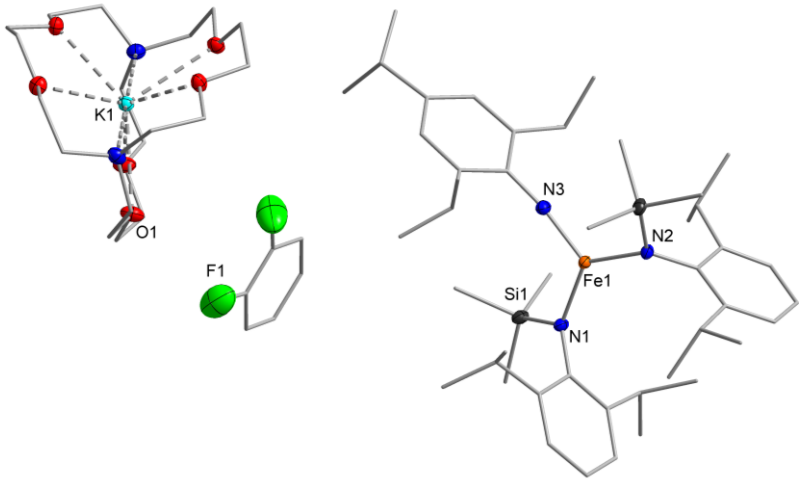

Figure S 54. Molecular structure of K{crypt.222}[Fe(NTripp)L_2], **4**.

Crystal data and structure refinements for K{crypt.222}[Fe(NDipp)(N{Tripp}SiMe$_3$)L], **5**

Empirical formula	$C_{63}H_{111}FeKN_5O_6Si_2$
Formula weight	1185.69
Temperature/K	100
Crystal system	monoclinic
Space group	$P2_1/c$
a/Å	14.5777(3)
b/Å	22.4019(7)
c/Å	23.3657(5)
α/°	90
β/°	92.243(2)
γ/°	90
Volume/Å^3	7624.6(3)
Z	4
ρ_{calc}g/cm^3	1.033
μ/mm^{-1}	0.328
F(000)	2580.0
Crystal size/mm^3	0.254 × 0.075 × 0.068
Radiation	Mo Kα (λ = 0.71073)
2Θ range for data collection/°	2.796 to 50.054
Index ranges	$-17 \le h \le 17$, $-26 \le k \le 26$, $-27 \le l \le 24$
Reflections collected	51117
Independent reflections	13458 [R_{int} = 0.0270, R_{sigma} = 0.0302]
Data/restraints/parameters	13458/0/723
Goodness-of-fit on F^2	1.046
Final R indexes [I>=2σ (I)]	R_1 = 0.0399, wR_2 = 0.1065
Final R indexes [all data]	R_1 = 0.0582, wR_2 = 0.1133
Largest diff. peak/hole / e Å^{-3}	0.80/−0.29

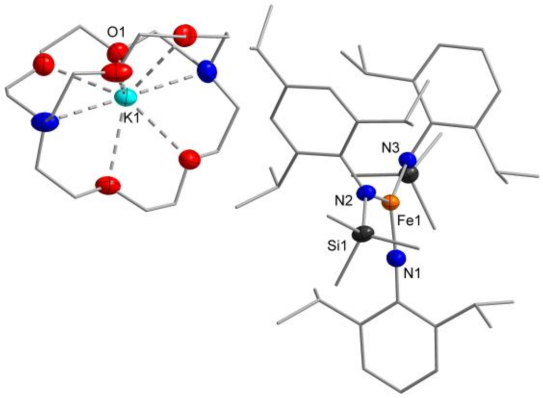

Figure S 55. Molecular structure of K{crypt.222}[Fe(NDipp)(N{Tripp}SiMe$_3$)L], **5.**

Crystal structure and refinements of K{18c6}[Fe(η^2-κN^1:κN^4-N_4Ph_2)L_2], **6**

Empirical formula	$C_{62}H_{102}FeKN_6O_8Si_2$
Formula weight	1210.651
Temperature/K	273.15
Crystal system	orthorhombic
Space group	$P2_12_12_1$
a/Å	22.4597(11)
b/Å	13.4554(7)
c/Å	21.8824(10)
α/°	90
β/°	90
γ/°	90
Volume/Å^3	6613.0(6)
Z	4
ρ_{calc}g/cm^3	1.216
μ/mm^{-1}	0.382
F(000)	2616.4
Crystal size/mm^3	0.437 × 0.114 × 0.064
Radiation	Mo Kα (λ = 0.71073)
2Θ range for data collection/°	3.98 to 53.58
Index ranges	$-28 \le h \le 28$, $-17 \le k \le 17$, $-27 \le l \le 27$
Reflections collected	167719
Independent reflections	14090 [R_{int} = 0.0499, R_{sigma} = 0.0264]
Data/restraints/parameters	14090/0/737
Goodness-of-fit on F^2	1.042
Final R indexes [I>=2σ (I)]	R_1 = 0.0460, wR_2 = 0.0957
Final R indexes [all data]	R_1 = 0.0485, wR_2 = 0.0968
Largest diff. peak/hole / e Å^{-3}	0.59/−0.54
Flack parameter	−0.028(3)

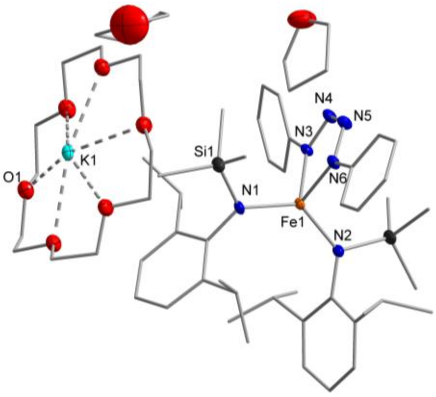

Figure S 56. Molecular structure of K{18c6}[Fe(η^2-κN^1:κN^4-N_4Ph_2)L_2], **6**, depicting two thf molecules in the unit cell.

Crystal data and structure refinements for [Fe(NTol)L_2], **7**

Empirical formula	$C_{74}H_{118}Fe_2N_6Si_4$
Formula weight	1315.834
Temperature/K	100.00
Crystal system	orthorhombic
Space group	$Pca2_1$
a/Å	20.1024(7)
b/Å	12.4529(4)
c/Å	30.5519(11)
α/°	90
β/°	90
γ/°	90
Volume/Å^3	7648.2(5)
Z	4
ρ_{calc}g/cm^3	1.143
μ/mm^{-1}	0.484
F(000)	2853.5
Crystal size/mm^3	0.291 × 0.14 × 0.122
Radiation	Mo Kα (λ = 0.71073)
2Θ range for data collection/°	3.84 to 53.52
Index ranges	−25 ≤ h ≤ 24, −15 ≤ k ≤ 15, −34 ≤ l ≤ 38
Reflections collected	108309
Independent reflections	15706 [R_{int} = 0.0471, R_{sigma} = 0.0342]
Data/restraints/parameters	15706/1/806
Goodness-of-fit on F^2	1.041
Final R indexes [I>=2σ (I)]	R_1 = 0.0345, wR_2 = 0.0769
Final R indexes [all data]	R_1 = 0.0369, wR_2 = 0.0779
Largest diff. peak/hole / e Å^{-3}	0.42/−0.28
Flack parameter	−0.012(4)

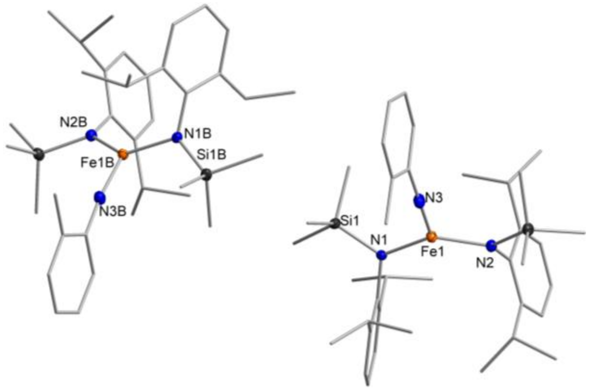

Figure S 57. Molecular structure of [Fe(NTol)L_2], **7**, with the depiction of a second molecule in the unit cell.

Crystal data and structure refinements for [Fe(NXyl)L_2], **8**.

Empirical formula	$C_{38}H_{61}FeN_3Si_2$
Formula weight	744.095
Temperature/K	100.00
Crystal system	triclinic
Space group	P-1
a/Å	11.4085(3)
b/Å	12.0707(4)
c/Å	17.7167(5)
α/°	82.812(1)
β/°	72.081(1)
γ/°	65.257(1)
Volume/Å^3	2108.26(11)
Z	2
ρ_{calc}g/cm^3	1.172
μ/mm^{-1}	3.640
F(000)	813.0
Crystal size/mm^3	0.259 × 0.14 × 0.116
Radiation	Cu Kα (λ = 1.54178)
2Θ range for data collection/°	5.24 to 144.88
Index ranges	−14 ≤ h ≤ 14, −14 ≤ k ≤ 14, −21 ≤ l ≤ 21
Reflections collected	51482
Independent reflections	8287 [R_{int} = 0.0366, R_{sigma} = 0.0228]
Data/restraints/parameters	8287/0/413
Goodness-of-fit on F^2	1.043
Final R indexes [I>=2σ (I)]	R_1 = 0.0422, wR_2 = 0.1234
Final R indexes [all data]	R_1 = 0.0436, wR_2 = 0.1259
Largest diff. peak/hole / e Å^{-3}	0.59/−0.34

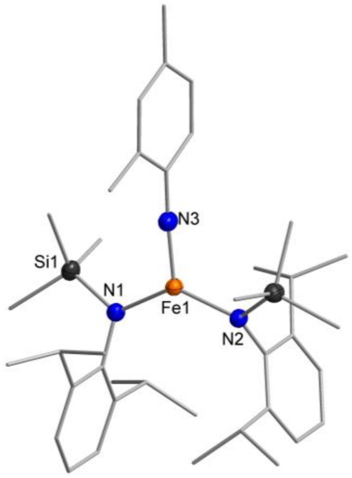

Figure S 58. Molecular structure of [Fe(NXyl)L_2], **7**.

Crystal structure and refinements for [Fe(κ^2-*N*-N(SiMe$_3$)-2-(CH$_3$)$_2$CNH{Ph}-6-^{i}Pr-phenyl)L], **9**.

Empirical formula	$C_{36}H_{57}FeN_3Si_2$
Formula weight	643.87
Temperature/K	100.00
Crystal system	monoclinic
Space group	$P2_1/c$
a/Å	10.3333(4)
b/Å	18.9504(7)
c/Å	19.4645(8)
α/°	90
β/°	103.6070(10)
γ/°	90
Volume/Å^3	3704.6(3)
Z	4
ρ_{calc}g/cm^3	1.154
μ/mm^{-1}	0.499
F(000)	1392.0
Crystal size/mm^3	0.278 × 0.125 × 0.086
Radiation	MoKα (λ = 0.71073)
2Θ range for data collection/°	4.056 to 53.512
Index ranges	−13 ≤ h ≤ 13, −23 ≤ k ≤ 23, −24 ≤ l ≤ 24
Reflections collected	67376
Independent reflections	7869 [R_{int} = 0.0394, R_{sigma} = 0.0211]
Data/restraints/parameters	7869/0/397
Goodness-of-fit on F^2	1.057
Final R indexes [I>=2σ (I)]	R_1 = 0.0297, wR_2 = 0.0705
Final R indexes [all data]	R_1 = 0.0336, wR_2 = 0.0724
Largest diff. peak/hole / e Å^{-3}	0.37/−0.25

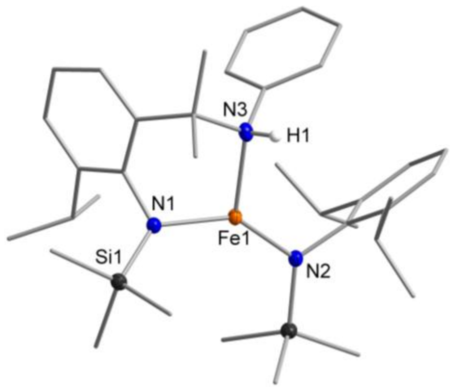

Figure S 59. Molecular structure of [Fe(κ^2-*N*-N(SiMe$_3$)-2-(CH$_3$)$_2$CNH{Ph}-6-^{i}Pr-phenyl)L], **9**.

Crystal structure and refinements for [Fe(κ²-*N*-N(SiMe$_3$)-2-(CH$_3$)$_2$CNH{Tol}-6-^{i}Pr-phenyl)L], **10**.

Empirical formula	$C_{74}H_{118}Fe_2N_6Si_4$
Formula weight	1315.834
Temperature/K	100.00
Crystal system	triclinic
Space group	P-1
a/Å	12.8329(4)
b/Å	13.0002(4)
c/Å	23.5373(8)
α/°	80.293(1)
β/°	88.262(2)
γ/°	76.526(1)
Volume/Å^3	3763.8(2)
Z	2
ρ_{calc}g/cm^3	1.161
μ/mm^{-1}	4.019
F(000)	1425.5
Crystal size/mm^3	0.233 × 0.126 × 0.077
Radiation	Cu Kα (λ = 1.54178)
2Θ range for data collection/°	3.8 to 156.38
Index ranges	−16 ≤ h ≤ 16, −16 ≤ k ≤ 15, −29 ≤ l ≤ 28
Reflections collected	69939
Independent reflections	15725 [R_{int} = 0.0614, R_{sigma} = 0.0474]
Data/restraints/parameters	15725/0/805
Goodness-of-fit on F^2	1.028
Final R indexes [I>=2σ (I)]	R_1 = 0.0447, wR_2 = 0.1169
Final R indexes [all data]	R_1 = 0.0515, wR_2 = 0.1231
Largest diff. peak/hole / e Å^{-3}	1.00/−0.54

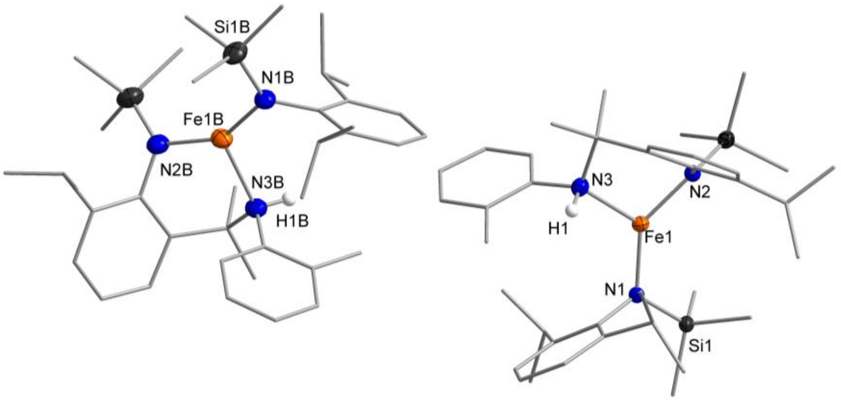

Figure S 60. Molecular structure of [Fe(κ²-*N*-N(SiMe$_3$)-2-(CH$_3$)$_2$CNH{Tol}-6-^{i}Pr-phenyl)L], **10**, depicting 2 molecules in the cell.

Crystal structure and refinements for [Fe(κ^2-*N*-N(SiMe$_3$)2-(CH$_3$)$_2$CNH{Xyl}-6-^{i}Pr-phenyl)L], **11**

Empirical formula	$C_{76}H_{122}Fe_2N_6Si_4$
Formula weight	1343.85
Temperature/K	273.15
Crystal system	triclinic
Space group	P-1
a/Å	12.0081(4)
b/Å	17.5397(6)
c/Å	19.5124(7)
α/°	88.8520(10)
β/°	72.8480(10)
γ/°	85.6290(10)
Volume/Å^3	3915.5(2)
Z	2
ρ_{calc}g/cm^3	1.140
μ/mm^{-1}	0.474
F(000)	1456.0
Crystal size/mm^3	0.068 × 0.06 × 0.04
Radiation	MoKα (λ = 0.71073)
2Θ range for data collection/°	4.394 to 53.49
Index ranges	$-15 \le h \le 15$, $-22 \le k \le 21$, $-24 \le l \le 24$
Reflections collected	125549
Independent reflections	16614 [R_{int} = 0.0796, R_{sigma} = 0.0460]
Data/restraints/parameters	16614/0/825
Goodness-of-fit on F^2	1.057
Final R indexes [I>=2σ (I)]	R_1 = 0.0497, wR_2 = 0.1011
Final R indexes [all data]	R_1 = 0.0663, wR_2 = 0.1075
Largest diff. peak/hole / e Å^{-3}	1.02/−0.53

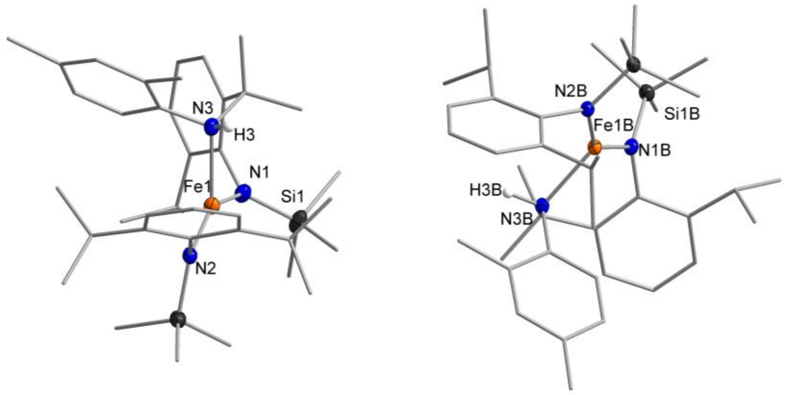

Figure S 61. Molecular structure of [Fe(κ^2-*N*-N(SiMe$_3$)-2-(CH$_3$)$_2$CNH{Xyl}-6-^{i}Pr-phenyl)L], **11**, depicting 2 molecules in the unit cell.

Crystal structure and refinements of K{crypt.222}[Fe(N{Tol}C{H}{Ph}O)L_2], **12**

Empirical formula	$C_{62}H_{101}FeKN_5O_7Si_2$
Formula weight	1179.637
Temperature/K	273.15
Crystal system	triclinic
Space group	P-1
a/Å	13.7022(8)
b/Å	14.0618(8)
c/Å	18.9131(11)
α/°	103.211(2)
β/°	99.162(2)
γ/°	108.526(2)
Volume/Å^3	3256.0(3)
Z	2
ρ_{calc}g/cm^3	1.203
μ/mm^{-1}	0.385
F(000)	1276.2
Crystal size/mm^3	0.43 × 0.216 × 0.216
Radiation	Mo Kα (λ = 0.71073)
2Θ range for data collection/°	4.2 to 53.6
Index ranges	−17 ≤ h ≤ 17, −17 ≤ k ≤ 17, −23 ≤ l ≤ 23
Reflections collected	61042
Independent reflections	13799 [R_{int} = 0.0781, R_{sigma} = 0.0693]
Data/restraints/parameters	13799/0/718
Goodness-of-fit on F^2	1.150
Final R indexes [I>=2σ (I)]	R_1 = 0.0780, wR_2 = 0.1612
Final R indexes [all data]	R_1 = 0.1115, wR_2 = 0.1899
Largest diff. peak/hole / e Å^{-3}	0.95/−0.93

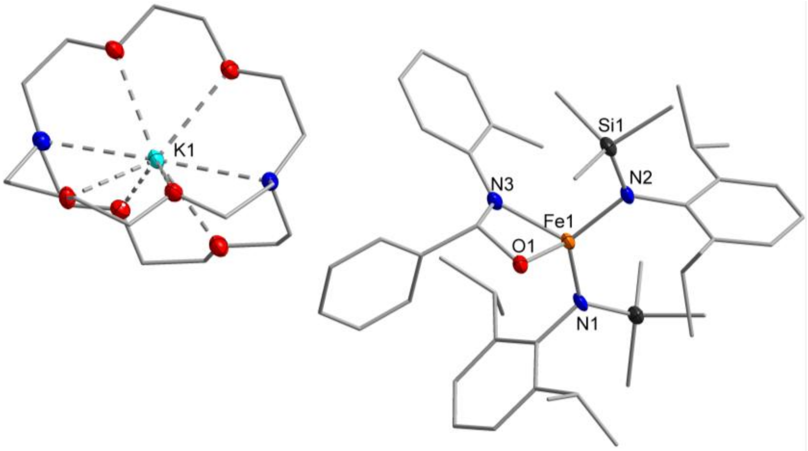

Figure S 62. Molecular structure of K{crypt.222}[Fe(N{Tol}C{H}{Ph}O)L_2] **12**.

Crystal structure and refinements of K{crypt.222}[Fe(N{Xyl}C{NMes}O)L_2], **13**

Empirical formula	$C_{66}H_{108}FeKN_6O_7Si_2$
Formula weight	1320.895
Temperature/K	129.00
Crystal system	triclinic
Space group	P-1
a/Å	14.2103(6)
b/Å	16.3146(7)
c/Å	18.4229(8)
α/°	98.721(2)
β/°	104.432(1)
γ/°	107.593(1)
Volume/Å^3	3821.8(3)
Z	2
ρ_{calc}g/cm^3	1.148
μ/mm^{-1}	0.335
F(000)	1436.2
Crystal size/mm^3	0.154 × 0.137 × 0.096
Radiation	Mo Kα (λ = 0.71073)
2Θ range for data collection/°	3.9 to 53.58
Index ranges	−17 ≤ h ≤ 18, −20 ≤ k ≤ 20, −23 ≤ l ≤ 23
Reflections collected	174060
Independent reflections	16235 [R_{int} = 0.0569, R_{sigma} = 0.0294]
Data/restraints/parameters	16235/0/767
Goodness-of-fit on F^2	1.050
Final R indexes [I>=2σ (I)]	R_1 = 0.0499, wR_2 = 0.1073
Final R indexes [all data]	R_1 = 0.0588, wR_2 = 0.1114
Largest diff. peak/hole / e Å^{-3}	0.69/−0.53

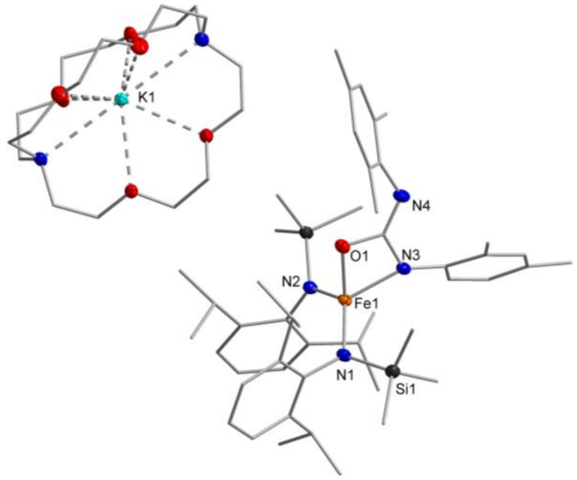

Figure S 63. Molecular structure of K{crypt.222}[Fe(N{Xyl}C{NMes}O)L_2], **13**.
